# Effect of Clinical Decision Support on Diagnostic Imaging for Pediatric Appendicitis: A Cluster Randomized Trial

**DOI:** 10.1001/jamanetworkopen.2020.36344

**Published:** 2021-02-09

**Authors:** Anupam B. Kharbanda, Gabriela Vazquez-Benitez, Dustin W. Ballard, David R. Vinson, Uli K. Chettipally, Steven P. Dehmer, Heidi Ekstrom, Adina S. Rauchwerger, Brianna McMichael, Dale M. Cotton, Mamata V. Kene, Laura E. Simon, Jingyi Zhu, E. Margaret Warton, Patrick J. O’Connor, Elyse O. Kharbanda

**Affiliations:** 1 Department of Pediatric Emergency Medicine, Children’s Minnesota, Minneapolis; 2 Division of Research, HealthPartners Institute, Minneapolis, Minnesota; 3 The Permanente Medical Group, Oakland, California; 4 The Kaiser Permanente Northern California Division of Research, Oakland, California

## Abstract

**Importance:**

Appendicitis is the most common pediatric surgical emergency. Efforts to improve efficiency and quality of care have increased reliance on computed tomography (CT) and ultrasonography (US) in children with suspected appendicitis.

**Objective:**

To evaluate the effectiveness of an electronic health record–linked clinical decision support intervention, AppyCDS, on diagnostic imaging, health care costs, and safety outcomes for patients with suspected appendicitis.

**Design, Setting, and Participants:**

In this parallel, cluster randomized trial, 17 community-based general emergency departments (EDs) in California, Minnesota, and Wisconsin were randomized to the AppyCDS intervention group or usual care (UC) group. Patients were aged 5 to 20 years, presenting for an ED visit with right-sided or diffuse abdominal pain lasting 5 days or less. We excluded pregnant patients, those with a prior appendectomy, those with selected comorbidities, and those with traumatic injuries. The trial was conducted from October 2016 to July 2019.

**Interventions:**

AppyCDS prompted data entry at the point of care to estimate appendicitis risk using the pediatric appendicitis risk calculator (pARC). Based on pARC estimates, AppyCDS recommended next steps in care.

**Main Outcomes and Measures:**

Primary outcomes were CT, US, or any imaging (CT or US) during the index ED visit. Safety outcomes were perforations, negative appendectomies, and missed appendicitis. Costs were a secondary outcome. Ratio of ratios (RORs) for primary and safety outcomes and differences by group in cost were used to evaluate effectiveness of the clinical decision support tool.

**Results:**

We enrolled 3161 patients at intervention EDs and 2779 patients at UC EDs. The mean age of patients was 11.9 (4.6) years and 2614 (44.0%) were boys or young men. RORs for CT (0.94; 95% CI, 0.75-1.19), US (0.98; 95% CI, 0.84-1.14), and any imaging (0.96; 95% CI, 0.86-1.07) did not differ by study group. In an exploratory analysis conducted in 1 health system, AppyCDS was associated with a reduction in any imaging (ROR, 0.82; 95% CI, 0.73- 0.93) for patients with pARC score of 15% or less and a reduction in CT (ROR, 0.58; 95% CI, 0.45-0.74) for patients with a pARC score of 16% to 50%. Perforations, negative appendectomies, and cases of missed appendicitis by study phase did not differ significantly by study group. Costs did not differ overall by study group.

**Conclusions and Relevance:**

In this study, AppyCDS was not associated with overall reductions in diagnostic imaging; exploratory analysis revealed more appropriate use of imaging in patients with a low pARC score.

**Trial Registration:**

ClinicalTrials.gov Identifier: NCT02633735

## Key Points

**Question:**

Can an electronic health record–linked clinical decision support tool reduce the use of diagnostic imaging in children and adolescents with emergency department visits for acute abdominal pain?

**Findings:**

In this cluster randomized trial of 17 emergency departments caring for 5940 patients, the clinical decision support tool did not lead to an overall reduction in the use of computed tomography or ultrasonography. In 1 health care system, the clinical support tool was associated with a significant reduction in diagnostic imaging among patients at low or moderate risk of appendicitis.

**Meaning:**

These findings suggest that more research is needed to determine whether clinical decision support tools promote more appropriate imaging in patients with acute abdominal pain.

## Teaser

This cluster randomized trial evaluates the effectiveness of an electronic health record–linked clinical decision support intervention on diagnostic imaging, health care costs, and safety outcomes for patients with suspected appendicitis.

## Introduction

Appendicitis is the most common pediatric surgical emergency, with more than 75 000 cases in the United States each year.^1^ Despite its high frequency, distinguishing appendicitis from other causes of acute abdominal pain remains a challenge.^2^ Pressures to improve emergency department (ED) efficiency, while preventing negative appendectomies and missed appendicitis, have contributed to heavy utilization of computed tomography (CT) and ultrasonography (US) in patients with suspected appendicitis.^3,4^

During the past 2 decades, there have been incremental improvements in the evaluation of patients with suspected appendicitis, especially at pediatric EDs,^5,6,7,8,9^ where efforts to reduce ionizing radiation exposure have shifted imaging from CT to US.^10,11,12^ Clinical pathways for acute abdominal pain have demonstrated the safety and effectiveness^5,13,14,15,16^ of US as first-line imaging.^14,15,16^ However, US can be problematic if applied indiscriminately, as it is operator dependent, and equivocal or nondiagnostic results are common.^17,18^ Unfortunately, CT use remains high for children with abdominal pain at community-based, general EDs.^3,4,19^ Differential management between pediatric and general EDs is concerning, considering that community-based sites are where most children across the United States present for emergent evaluation.^20^

We previously developed and validated the pediatric appendicitis risk calculator (pARC).^21^ Subsequently, we created a clinical decision support (CDS) system, AppyCDS, integrating pARC with the electronic health record (EHR). Using EHR and web-based algorithms, AppyCDS prompts health care clinicians to screen patients at risk of appendicitis and recommends next steps in care based on calculated risk or pARC score.^22^ The primary aim of this study was to evaluate the impact of AppyCDS on use of CT or US during an ED visit among pediatric and adolescent patients with acute abdominal pain. Secondary aims were to evaluate the impact of AppyCDS on health care costs and safety outcomes.

## Methods

We evaluated AppyCDS in a pragmatic (ie, the intervention was tested in real-world clinical settings), parallel cluster randomized trial in 17 community-based general EDs within 2 large health systems. Of these, 6 were HealthPartners (HP) facilities in Minnesota and Western Wisconsin, with a combined annual ED census of 160 000 visits. Eleven EDs were Kaiser Permanente Northern California (KPNC) facilities, located across Northern California, with a combined annual ED census of 800 000 visits. This study was approved by institutional review boards from participating sites with a waiver of informed consent because it posed minimal risk to patients. This study followed the Consolidated Standards of Reporting Trials (CONSORT) reporting guideline. The trial protocol and statistical analysis plan are available in Supplement 1. For both health systems, patients aged 5 to 20 years old account for approximately 20% of ED visits. All EDs use the Epic EHR. Neither health system is university-based; 5 EDs have academic affiliations. Facilities were staffed by board-certified or board-eligible emergency physicians. Physician assistants also provided care at HP EDs. All EDs had access to CT and US during regular business hours; after-hours US availability varied by facility. Age thresholds to admit for observation or surgery (vs transfer to a dedicated pediatric facility) also varied by ED.

### Design, Study Population, and Randomization

We conducted the trial between October 2016 and July 2019, with 2 study phases, ie, preintervention and intervention. Detailed descriptions of the AppyCDS intervention have been published.^22^ The timing of AppyCDS implementation varied by health system (eFigure in Supplement 2). At HP, following a 5-month pilot, the preintervention phase ran from November 2016 to April 2017. After a 1-month washout period, the intervention phase ran from June 2017 to July 2019. At KPNC, following a 2-month pilot, the preintervention phase ran from October 2016 to June 2017. After a 1-month washout period, the intervention phase ran from August 2017 to July 2019. Data from pilot and washout periods were not included in analyses.

At all sites, patients aged 5 to 20 years presenting to the ED with a chief complaint of abdominal pain were initially assessed using 3 study-specific screening questions, displayed in the EHR, as follows: (1) is the patient’s abdominal pain diffuse (ie, generalized)? (2) does the patient have any right-sided abdominal pain? and (3) what is the duration of abdominal pain? Screening questions were completed by nursing staff (at HP) or treating physicians (at KPNC) prior to evaluation. Patients with 5 days or fewer of right-sided or diffuse abdominal pain were eligible and enrolled. Automated exclusions were applied in real time based on data in the EHR.^22^ Additional exclusions were applied during analysis (eTable 1 in Supplement 2).

Sites were randomized to the AppyCDS intervention or usual care (UC) in January 2017. We stratified EDs by health system and then grouped them into clusters by patterns of pediatric referral and health care professional coverage. We sorted clusters into pairs by pediatric patient volume. Using a random-number generator, 6 clusters (9 EDs) were randomly assigned to AppyCDS intervention; 6 clusters (8 EDs) were assigned to UC.

### Description of Intervention

The overall goals of AppyCDS were as follows: (1) to collect data to calculate risk of appendicitis (pARC score) and (2) to provide point-of-care decision support, recommending next steps in care based on pARC score. As previously described, AppyCDS platforms differed by health system to accommodate existing workflows and EHR-linked CDS infrastructure.^22^ At HP, clinicians at intervention sites were directed by best practice advisories (BPAs) to use AppyCDS; BPAs displayed for patients who were study eligible when the clinician opened the medical record or attempted to order a CT or US. At KPNC, physicians were trained to access AppyCDS through an existing EHR-linked CDS platform, RISTRA (Risk Stratification).^23,24^ In addition, KPNC physicians were notified of potentially eligible patients through automated text message alerts.^25^ At KPNC, physicians accessing AppyCDS completed initial screening questions, identified exclusions, and, for eligible patients, proceeded with data entry to calculate a pARC score. Across all intervention sites, recommendations for patient management displayed based on pARC score (eTable 2 in Supplement 2). Recommendations were tailored by health system, based on resource availability and consensus from clinical leaders.

### Description of UC

At all HP sites preintervention and at HP UC sites during the intervention, no additional data were collected. At all KPNC sites during preintervention and at KPNC UC sites during the intervention, additional clinical information was entered by health care professionals into AppyCDS, but pARC scores and recommended next steps in care were not displayed.

### Outcomes

The primary outcome was utilization of diagnostic imaging (ie, CT, US, or any imaging [CT or US]) during the index ED visit. Use of diagnostic imaging was identified through *Current Procedural Terminology* (*CPT*) or internal codes.

Appendicitis was confirmed by manual review of pathology and operative notes for appendectomies within 7 days of the index ED visit with definitions based on our prior work.^15,21^ When operative or pathology reports were not available, diagnosis was based on data at the index ED visit.

Safety outcomes included negative appendectomies, perforation, and missed appendicitis. Negative appendectomy was defined as a nonincidental appendectomy within 7 days of the index visit with no evidence for appendicitis on histopathology. Perforation was based on manual review of operative notes. Missed appendicitis was defined as appendicitis within 7 days of the index visit among patients discharged home. Appendicitis outcomes were reviewed by 2 study investigators (D.W.B. and E.O.K.) with adjudication by a third investigator (A.B.K.) as needed.

Secondary outcomes included ED length of stay, disposition, ED visits and intensive care admissions within 7 days of the index visit, severe medical conditions (eg, pyelonephritis, nephrolithiasis), and other surgical conditions (eg, intussusception, ovarian torsion). Among patients insured through HP or KPNC, costs of care for the index ED visit and the subsequent 14 days were evaluated using claims data. Costs of care in 2018 US dollars were derived by matching billing codes to Total Care Relative Resource Values,^26^ a standardized set of measures endorsed by the National Quality Forum for calculating the total cost of care.^27^ Pharmacy costs were limited to antibiotics, antiemetics, and pain medications.

### Statistical Analysis

First, we evaluated patient characteristics and use of imaging by study group and phase using frequency distributions, means, and standard deviations. We evaluated the effectiveness of AppyCDS by estimating the ratio of ratios (RORs) and 95% CIs of the intervention group, from intervention phase to preintervention phase, compared with the UC group for the 2 study phases for primary and safety outcomes. To estimate the ROR, we used a general estimating equation (GEE) Poisson model with a study group indicator, a study phase indicator, a study group–study phase interaction term, and health system as fixed effects. We evaluated whether adding patient characteristics into the model changed the ROR estimates. The GEE model accounted for the data structure, patients within each ED, and randomization by cluster, with a repeat statement and compound symmetry covariance structure. For safety and secondary binary outcomes, similar methods were used. Length of ED stay and hospitalization were estimated using a log normal distribution. Costs were estimated with a GEE model using a γ distribution with a log link for patients enrolled during the intervention phase. For KPNC, where pARC values were collected for all enrolled patients, we evaluated imaging outcomes by pARC strata in exploratory analyses, using a second-order interaction. Furthermore, we evaluated whether the AppyCDS intervention significantly reduced imaging in patients with pARC scores of 50% or less, consistent with the AppyCDS tool recommendations for this low-risk group (eTable 2 in Supplement 2). Analysis for effectiveness of the AppyCDS system was evaluated with a 2-sided test at *P* < .05 level of significance. Analysis was performed using SAS version 9.4 (SAS Institute). A priori power analysis assumed that with 12 clusters, baseline CT utilization of 30%, 600 patients per cluster during the intervention period, α of .05 with a 2-tailed test, and an intraclass cluster correlation of 0.05, the study would have 80% power to identify an ROR of 0.76, contrasting the post-pre ratios of CT in the intervention vs the UC group.

## Results

Between October 2016 and July 2019 there were 40 283 patients aged 5 to 20 years with visits to participating EDs with abdominal pain. Of these, 8605 (21.4%) had an a priori exclusion (eg, prior appendectomy, pregnancy, trauma, other selected comorbidities, or visit during washout period). We excluded 949 patients (2.4%) at intervention sites and 703 (1.7%) at UC sites, based on data entered into AppyCDS indicating pain was not right-sided or generalized or had lasted for more than 5 days (Figure). An additional 12 945 (32.1%) at intervention sites and 11 140 (27.7%) at UC sites did not have data entered in AppyCDS, so their eligibility could not be evaluated. The final enrolled population with 5 days or less of right-sided or diffuse abdominal pain included 3161 patients at intervention EDs (1011 [32.0%] preintervention phase; 2150 [68.0%] intervention phase) and 2779 patients at UC EDs (788 [28.4%] preintervention phase; 1991 [71.6%] intervention phase). Mean age was 11.9 (4.6) years, and 2614 (44.0%) were boys or young men. Patients at UC sites were more likely to be White, non-Hispanic individuals compared with those at intervention sites (preintervention UC, 311 [39.5%]; intervention UC, 901 [45.2%] vs preintervention AppyCDS, 234 [23.2%]; intervention AppyCDS, 721 [34.5%]) (Table 1). Proportions with appendicitis ranged from 11.1% (112 of 1011) to 13.5% (290 of 2150) by study group and study phase (Table 2). Comparison of patients who were not enrolled vs those who were enrolled revealed a higher mean (SD) age (13.4 [5.0] years vs 11.8 [4.5] years) and a higher proportion of female patients (15 166 of 24 203 [62.7%] vs 3293 of 5940 [55.4%]).

**Figure.**
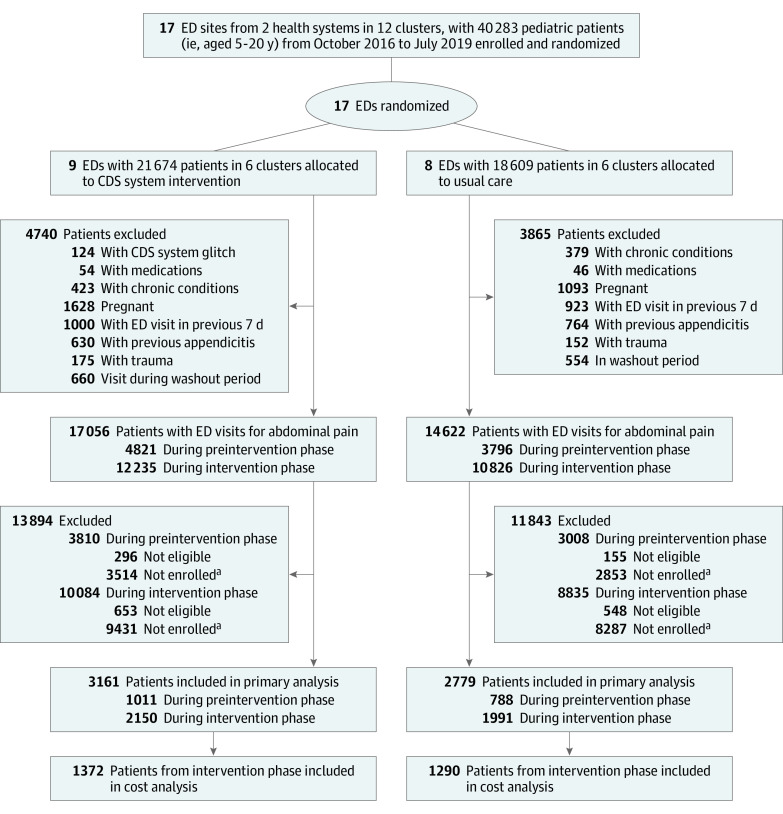
Flowchart of Patients Who Were Eligible, Excluded, and Enrolled by Intervention and Usual Care Group Cost analyses were limited to enrolled subjects insured through the health system (HealthPartners or Kaiser Permanente, Northern California). CDS indicates clinical decision support; ED, emergency department. ^a^Not enrolled indicates that no patient information was entered in the clinical decision support system.

**Table 1.** Study Population Characteristics by Intervention Group and Study Phase

Characteristic	No. (%)			
	AppyCDS group		Usual care group	
	Preintervention phase (n = 1011)	Intervention phase (n = 2150)	Preintervention phase (n = 788)	Intervention phase (n = 1991)
Age, mean (SD), y	11.0 (4.3)	12.4 (4.6)	11.0 (4.3)	11.9 (4.6)
Race/ethnicity				
Asian	127 (12.6)	240 (11.0)	45 (5.7)	131 (6.6)
Black	154 (15.2)	319 (15.1)	110 (14.0)	292 (14.7)
Hispanic	403 (30.9)	701 (31.6)	256 (32.5)	532 (26.7)
Other^a^	93 (9.2)	169 (7.8)	66 (8.4)	135 (6.8)
White	234 (23.2)	721 (34.5)	311 (39.5)	901 (45.2)
Female patients	559 (55.3)	1221 (56.8)	420 (53.3)	1092 (54.9)
Site				
HP	170 (16.8)	721 (33.5)	52 (6.6)	572 (28.7)
KPNC	841 (83.2)	1429 (66.5)	736 (93.4)	1419 (71.3)

Abbreviations: HP, HealthPartners; KPNC, Kaiser Permanente, Northern California.

^a^ Other category includes American Indian or Alaska Native, Native Hawaiian, more than 1 race, and unknown race.

**Table 2.** Appendicitis, Imaging Use, and Safety Outcomes During the Index Visit by Intervention Group and Study Phase^a^

Outcome	AppyCDS group			Usual care group			Intervention/usual care, ratio of ratios (95% CI)
	No. (%)		Ratio by phase (95% CI)	No. (%)		Ratio by phase (95% CI)	
	Preintervention phase (n = 1011)	Intervention phase (n = 2150)		Preintervention phase (n = 788)	Intervention phase (n = 1991)		
Appendicitis	112 (11.1)	290 (13.5)	1.26 (1.06-1.50)	95 (12.1)	246 (12.4)	1.06 (0.80-1.40)	1.19 (0.86-1.66)
CT use	232 (22.9)	538 (25.0)	1.07 (0.97-1.17)	142 (18.0)	438 (22.0)	1.13 (0.92-1.40)	0.94 (0.75-1.19)
US use	485 (48.0)	986 (45.9)	1.01 (0.91-1.12)	397 (50.4)	958 (48.1)	1.03 (0.92-1.16)	0.98 (0.84-1.14)
Any imaging^b^	586 (58.0)	1237 (57.5)	1.02 (0.94-1.11)	455 (57.7)	1163 (58.4)	1.06 (0.98-1.14)	0.96 (0.86-1.08)
Perforation^c^	20 (17.9)	43 (14.8)	0.80 (0.48-1.35)	17(17.9)	50 (20.3)	1.09 (0.80-1.48)	0.74 (0.41-1.32)
Negative appendectomy^d^	9 (7.4)	11 (3.7)	0.52 (0.28-0.94)	8 (7.8)	19 (7.3)	1.02 (0.36-2.89)	0.51 (.16-1.66)
Missed appendicitis, No./total No. (%)^e^	2/845 (0.2)	11/1782 (0.6)	2.62 (1.28-5.34)	5/657 (0.8)	10/1660 (0.6)	0.92 (0.47-1.83)	2.83 (1.0-7.68)

Abbreviations: CT, computed tomography; US, ultrasonography.

^a^ No statistical significance by study group–pre-post interaction χ^2^ test (CT, *P* = .62; US, *P* = .79; any imaging, *P* = .53; appendicitis, *P* = .31; perforation, *P* = .30; negative appendectomy, *P* = .29; and missed appendicitis, *P* = .10); general estimating equation Poisson model had pre-post, study group, study group–study phase interaction term, and health system as fixed effects, and compound symmetry covariance structure for emergency department facility.

^b^ Any imaging signifies CT or US.

^c^ Perforations among patients with appendicitis, based on operative note or clinical adjudication for patients undergoing draining or antibiotic treatment with interval appendectomy.

^d^ Negative appendectomy based on pathology report for patients undergoing immediate appendectomy.

^e^ Missed appendicitis within 7 days of index emergency department visit, based on clinical adjudication, of patients discharged home at index visit.

### Primary Outcomes

There was no statistically significant change in CT use from preintervention to intervention phase between study groups (ROR, 0.94; 95% CI, 0.75-1.19) (Table 2). A nonsignificant decrease in US use was noted between preintervention and intervention phases at intervention and UC sites (485 of 1011 [48.0%] to 986 of 2150 [45.9%] and 397 of 788 [50.4%] to 958 of 1991 [48.1%], respectively; ROR, 0.98; 95% CI, 0.84-1.14). At intervention sites, any imaging decreased nonsignificantly from 586 (58.0%) in the preintervention phase to 1237 (57.5%) in the intervention phase. At UC sites, there was a nonsignificant increase in any imaging, from 455 (57.7%) in the preintervention phase to 1163 (58.4%) in the intervention phase. The ratio of any imaging by study phase did not differ by study group (ROR, 0.96; 95% CI, 0.86-1.08). After adjusting for age, sex, and race, results were similar (CT: ROR, 0.92; 95% CI, 0.76-1.06; US: ROR, 0.98; 95% CI, 0.83-1.14; any imaging: ROR, 0.95; 95% CI, 0.84-1.06).

### Exploratory Analyses

At KPNC, where pARC scores were calculated for all enrolled patients, AppyCDS was associated with a reduction in any imaging (ROR, 0.82; 95% CI, 0.73-0.93) for pARC scores of 15% or less and a reduction in CT (ROR, 0.58; 95% CI, 0.45-0.74) for pARC scores between 16% and 50% (Table 3). However, there was no overall association between pARC strata and imaging.

**Table 3.** Imaging Use by pARC Risk Stratification, Study Group and Phase, at Kaiser Permanente Northern California Sites

pARC score	AppyCDS group		Usual care group		Ratio of ratios (95% CI)^a^
	Preintervention phase (n = 510)	Intervention phase (n = 1054)	Preintervention phase (n = 490)	Intervention phase (n = 997)	
≤15%					
CT use	73 (26.1)	105 (19.1)	49 (19.3)	84 (15.9)	0.85 (0.54-1.33)
US use	178 (63.6)	291 (52.8)	159 (62.6)	327 (62.0)	0.88 (0.77-0.99)
Any imaging^b^	207 (73.9)	331 (60.1)	177 (69.7)	368 (69.8)	0.82 (0.73-0.93)
16%-50%					
CT use	63 (46.7)	112 (38.0)	38 (27.9)	109 (38.5)	0.58 (0.45-0.74)
US use	103 (76.3)	199 (67.5)	107 (78.7)	207 (73.1)	1.04 (0.92-1.17)
Any imaging^b^	126 (93.3)	240 (81.4)	121 (89.0)	245 (86.6)	0.94 (0.83-1.06)
51%-75%					
CT use	25 (45.5)	70 (56.5)	31 (47.7)	50 (47.2)	1.12 (0.64-1.95)
US use	37 (67.3)	89 (71.8)	54 (83.1)	76 (71.7)	1.19 (0.95-1.50)
Any imaging^b^	51 (92.7)	117 (94.4)	63 (96.9)	99 (93.4)	1.02 (0.92-1.12)
>75%					
CT use	19 (47.5)	48 (57.1)	5 (14.3)	35 (43.2)	0.45 (0.18-1.11)
US use	30 (75.0)	58 (69.0)	29 (82.9)	63 (77.8)	0.98 (0.73-1.30)
Any imaging^b^	37 (92.5)	81 (96.4)	33 (94.3)	79 (97.5)	1.02 (0.91-1.15)

Abbreviations: CT, computed tomography; pARC, pediatric appendicitis risk calculator; US, ultrasonography.

^a^ A test for heterogeneity, with 3 degrees of freedom, was conducted across pARC strata (CT, *P* = .40; US, *P* = .12; any imaging, *P* = .10).

^b^ Any imaging signifies CT or US.

### Safety, Secondary Outcomes, and Cost Analysis

Perforation rates decreased over time at intervention sites (20 [17.9%] to 43 [14.8%]) and increased at UC sites (17 [17.9%] to 50 [20.3%]); the ratio of perforation by phase did not differ significantly by study arm (ROR, 0.74; 95% CI, 0.41-1.32). Negative appendectomies decreased at both intervention sites (9 [7.4%] to 11 [3.7%]) and at UC sites (8 [7.8%] to 19 [7.3%]); however, the ratio by study arm was not significant (ROR, 0.51; 95% CI, 0.16-1.66). Missed appendicitis was uncommon in intervention and UC arms across study phases; changes in missed appendicitis rates between the preintervention and intervention phases, by study group, were not significant (ROR, 2.83; 95% CI, 1.0-7.7) (Table 2).

Most patients were discharged home following their ED visit with minimal variation by study group and study phase. Mean ED length of stay increased minimally at intervention sites and remained constant at UC sites. Additional secondary outcomes did not differ by study phase by study group (eTable 3 in Supplement 2).

A total of 1372 patients in the intervention arm and 1290 patients in the UC arm had HP or KPNC insurance and were included in cost analyses (Figure). Overall health care costs did not significantly differ by study group. Nevertheless, total outpatient costs on days 4 to 14 after the index visit were significantly lower at intervention sites compared with UC sites (difference, −$31; 95% CI, −$47 to −$15) (Table 4; eTable 4 in Supplement 2).

**Table 4.** Costs of Care Within 14 Days After Index Emergency Department Visit by Study Group During Intervention Phase^a^

Category	Cost, $		Difference (95% CI), $	*P* value
	AppyCDS group (n = 1372)	Usual care group (n = 1290)		
Total costs	1995	2020	−25 (−260 to 210)	.83
Total outpatient costs	993	1035	−42 (−172 to 88)	.52
Index ED visit	633	620	13 (−79 to 106)	.78
CT imaging	112	107	5 (−31 to 41)	.80
US imaging	78	81	−3 (−31 to 26)	.85
All other outpatient costs	360	410	−50 (−110 to 9)	.10
Within 3 d of index visit	284	302	−18 (−68 to 33)	.49
4-14 d From index visit	78	108	−31 (−47 to −15)	<.001
Total inpatient costs	947	960	−13 (−312 to 286)	.93
Pharmacy costs	17	20	−3 (−8 to 2)	.28

Abbreviations: CT, computed tomography; ED, emergency department; US, ultrasonography.

^a^ All costs were measured in 2018 US dollars during 14 days following and including the index ED visit for patients in each study group during the intervention phase. Total costs equal the sum of total outpatient costs, total inpatient costs, and pharmacy costs. All other outpatient costs include all outpatient costs (including emergency department) observed outside of the index visit. Pharmacy costs were limited to prescription fills for antibiotics, antiemetics, and pain medications. Subgroup totals will not necessarily sum to grand totals due to independently modeling costs within each group while accounting for cluster randomization across 2 sites.

## Discussion

In this multisite pragmatic cluster randomized trial, AppyCDS did not reduce CT or US use for the full spectrum of pediatric patients with suspected appendicitis. However, in an exploratory analysis, patients with a low (≤15%) or moderate (16%-50%) pARC scores underwent less imaging. Our study demonstrated the feasibility and safety of conducting a large randomized trial to improve care for children presenting for care in general, community-based EDs, while highlighting challenges and areas for improvement in future interventions.

The pARC score was designed to quantify risk of appendicitis and maximize sorting of patients as having high or low risk of appendicitis.^21^ Each participating health system developed care recommendations based on pARC strata, with input from clinical leaders. At intervention sites, clinicians were significantly more likely to defer imaging for patients with a less than 15% risk of appendicitis, consistent with AppyCDS recommendations. For those with a risk of 16% to 50%, we recommended US as first-line imaging, and accordingly, we noted a significant decrease in use of CT at intervention sites. Consistent with data from the National Surgical Quality Improvement Program for Pediatrics, patients with high pARC scores (>75%) continued to undergo diagnostic imaging at high rates (>90%).^3^ These results suggest that intervention EDs moved toward more appropriate imaging.^30^

Our findings are also consistent with prior research demonstrating low CDS use in acute health care settings.^28,29^ AppyCDS was designed to provide recommendations in real time to influence decision-making; however, alerts to complete the screening or use the CDS were easy to dismiss or ignore. The CDS system was developed in collaboration with end-users, yet fully automated estimation of pARC using EHR data may be preferred to improve efficiency and adherence. Requiring clinicians to provide a reason for overriding the CDS system or providing real-time feedback has also been demonstrated to improve use of CDS systems.^31,32^

Lack of agreement with recommendations, along with system-level barriers, may have limited adherence with AppyCDS guidance.^33^ For example, calculation of appendicitis risk through pARC required a white blood cell count, yet parents may have preferred imaging to confirm a diagnosis without a blood draw. For patients at low to moderate risk (ie, pARC score of 16%-25%), the CDS recommended observation prior to imaging, but this may be impractical in busy general EDs with limited space for pediatric patients. For many community-based EDs, AppyCDS recommendations represented a deimplementation of care (ie, forgoing US in patients at low risk of appendicitis). Efforts to deimplement care in the ED may require multilevel approaches that cannot be delivered through CDS systems alone. Ultimately, shared decision-making between families and clinicians,^34^ based on pARC score, along with health system support,^35^ may be needed to optimize diagnostic imaging in pediatric patients with acute abdominal pain.^36^

### Limitations

Several important limitations should be noted. There were a large number of patients who were not enrolled at intervention and control sites. As previously described, the rate of appendicitis among these patients was low^22^; it is likely that physicians ignored alerts to use AppyCDS because they had low suspicion for appendicitis. Improved capture of these patients may have increased the generalizability of our results but would also have resulted in a lower rate of appendicitis. The clinical characteristics of our enrolled population were consistent with prior publications describing children with acute abdominal pain.^15,21,37^ Regardless, our findings demonstrated the challenges we faced trying to evaluate and improve care for children in community-based general EDs. Barriers to CDS systems adherence may be amplified in settings where children are only a fraction of the patient population, and thus, alerts to complete CDS screening questions were infrequent. Additional barriers to CDS system use, such as pressure to reduce ED length of stay, lack of consistent availability of US, financial concerns of families, and fear of litigation due to missed appendicitis, were not measured. To conduct this trial in 2 health systems across 17 hospitals, it was necessary to provide system-level recommendations for care and differences in integration of the CDS system.^22^ However, this approach did not account for varying levels of risk tolerance among physicians and surgeons. Furthermore, our primary a priori study aim was to demonstrate an overall reduction in diagnostic imaging. A more nuanced approach to outcome assessment may have been more appropriate and was consistent with our findings. To achieve sustained reductions in diagnostic imaging, further studies should evaluate targeted approaches to US based on appendicitis risk.

## Conclusions

In this cluster randomized trial, we demonstrated the feasibility and safety of an EHR-linked appendicitis CDS tool in community-based EDs. Although we did not show an overall reduction in imaging, exploratory analyses at 1 health system revealed more appropriate use of diagnostic imaging.

## Notes

Supplement 1. **Trial Protocol and Statistical Analysis Plan**

Supplement 2. **eTable 1. **Exclusions Applied in Real Time and Post Hoc and Their Data Sources **eTable 2. **Recommendations by Pediatric Appendicitis Risk Calculator (pARC) and by Site **eTable 3. **Secondary Outcomes by Study Group and Phase **eTable 4. **Outpatient Costs of Care, Excluding Index Emergency Department Visit, by Study Group and Time From Index Visit During Intervention Phase **eFigure. **Calendar Month and Year for Start and End of Pilot, Preintervention, Washout, and Intervention Phases by Site

Supplement 3. **Data Sharing Statement**
