# Supplementary material for: Effect of Clinical Decision Support on Diagnostic Imaging for Pediatric Appendicitis: A Cluster Randomized Trial
Source: JAMA Netw Open. 2021 Feb 9;4(2):e2036344. doi: 10.1001/jamanetworkopen.2020.36344 (PMC7873779; doi:10.1001/jamanetworkopen.2020.36344)
Supplement: Supplement 1. — Trial Protocol and Statistical Analysis Plan [file jamanetwopen-e2036344-s001.pdf]

Manual of Operations

EHR-based Decision Support for Pediatric Acute Abdominal Pain in Emergency Care (Appy-CDS)

NCT02633735

Appy CDS is designed to reduce the reliance on diagnostic imaging for pediatric and adolescent patients with acute abdominal pain.

Anupam Kharbanda, MD, MSc
Elyse Kharbanda, MD, MPH

Contents

Abbreviations, Acronyms and Definitions 4

1) Summary 5

2) Introduction 6

a) Background 6

b) Rationale 7

c) Innovation 9

d) Preliminary Studies 10

3) Study Sites and Population 12

a) Exclusion Criteria 12

b) Patient Recruitment 12

4) Allocation of Interventions 13

5) Description of Intervention 13

a) Design at HealthPartners 13

b) Design at Kaiser Permanente 14

c) Implementation of the Appy-CDS Intervention 15

At Control and Intervention Sites 16

At Intervention Sites Only 16

6) Plan of Analysis and Analytic Models 17

a) Development of a Risk-Prediction Estimate for use in Appy-CDS 17

Hypothesis 1 18

Hypotheses 2 and 3 19

Hypothesis 4 – resource utilization: 19

b) Measurement of Outcomes 21

7) Reporting and Data Storage 24

a) Capturing Transfer Patients 24

b) Ongoing reporting 25

8) Protection of Human Subjects 26

a) Risks to Subjects 27

b) Adequacy of Protection Against Risks 28

c) Potential Benefits of the Proposed Research to the Subjects and Others 29

9) Data Safety and Monitoring 30
10) Project Organization and Management 39
a) Purpose of Document 40
b) Yearly Medical Center IRB Reviews 40
c) Participating Sites and Contacts 40
d) Directory of Study Personnel 41
e) Advisory Board Members 42
11) Investigator and Research Staff Responsibilities 43
a) IRB related issues 43
b) Study related issues 43
c) General Responsibilities 43
12) Literature Cited 45
Appendix A: Intervention Design and Workflow at HP 52
a) Capturing eligibility: Epic Triage BPA 52
a) Workflow 53
c) Full CDS-HP sites 54
d) Recommendations by Risk Group 58
Appendix B: Intervention Design and Workflow- KP 59
a) Capturing eligibility: RISTRA 59
b) Full CDS-KPNC sites 61
c) Recommendations by Risk Group 65
Appendix C: Reporting 67
a) Example enrollment report 67
Appendix D: Data Collection Instruments 68
Appendix E: Data Dictionary 76

Abbreviations, Acronyms and Definitions

BPA:	Best Practice Advisory
CDS:	Clinical Decision Support
CT:	Computed tomography
ED:	Emergency Department
EHR:	Electronic Health Record
FAQ:	Frequently Asked Questions
HP:	HealthPartners
ICU:	Intensive Care Unit
IRB:	Institutional Review Board
KPNC:	Kaiser Permanente Northern California
MOO:	Manual of Operations
N/A:	Not applicable
OR:	Operating Room
PI:	Principal Investigator
RA:	Research Assistant
US:	Ultrasound

1) Summary

Abdominal pain is one of the most common reasons for children and adolescents to seek care in the emergency department (ED). Computed tomography (CT) has been promoted as a method to improve diagnostic accuracy when evaluating patients with acute abdominal pain. In the past 20 years, CT use has increased dramatically, especially for children receiving care in general ED settings. Although in some adult cohorts, increased CT use has been associated with decreased rates of negative appendectomies, similar improvements in health outcomes among children with acute abdominal pain have not occurred. Negative consequences of CT include increased costs and substantial exposure to ionizing radiation.

Although appendicitis is the most common surgical emergency in children, its diagnosis remains a challenge and thus, emergency department (ED) providers increasingly rely on computed tomography to distinguish appendicitis from other conditions. This project (a) uses electronic health record (EHR) technology to deliver patient-specific clinical decision support to ED providers at the point of care, (b) assesses the impact of this intervention on the use of diagnostic imaging and clinical outcomes, and (c) assesses the impact of the intervention on the costs of care delivered.

Appy CDS is an automated, evidence-based tool designed to provide point-of-care clinical decision support (CDS) to providers for pediatric patients presenting to the emergency department (ED) with acute abdominal pain. Appy CDS uses Epic and web-based algorithms to provide recommendations, consistent with standard of care and informed by a personalized appendicitis risk calculation. The Appy CDS will be implemented and evaluated within two large health systems, HealthPartners (HP) and Kaiser Permanente Northern California (KPNC). The overall approach and algorithms driving the appendictis risk calculation within Appy CDS will be the same at the KPNC and HP sites. However, the actual implementation and appearance of the CDS may vary, based on local differences between the two health systems in: workflow, current EHR structure, and preferences of clinical leaders.

If successful, this flexible decision support tool could be adapted and implemented broadly in a range of acute care settings to both standardize and personalize care delivered to pediatric patients.

2) Introduction

a) Background

Acute abdominal pain is the chief complaint for 5%-10% of all emergency department (ED) visits among children.[1-4] Up to 10% of these patients have appendicitis, the most common surgical emergency in this age group.[2] Due to overlap in presentation between appendicitis and nonsurgical conditions, identifying the etiology of acute abdominal pain can be difficult. Thus, ED providers increasingly use computed tomography (CT) to diagnose appendicitis.[5,6] From 1998-2008, in a national sample of pediatric ED visits, CT use among children with abdominal pain increased from 0.9% to 15.4%.[4] Furthermore, children receiving care at general EDs were more likely than those at pediatric EDs to undergo CT.[4,7] In fact, general facilities are both the predominant providers of emergent care[8] in the United States and the major source of ionizing radiation exposure in children.[9] CT use in children is associated with increased costs[10,11] and adverse health effects[12-14] without consistent clinical benefits.[4,5,15-19]

Previously, our group and others have developed and validated clinical decision rules for children with acute abdominal pain.[20-23] Goals of these rules have been to identify: 1) patients at high risk for appendicitis who do not require imaging before surgery; and 2) patients at low risk for appendicitis, for whom the risks of CT may outweigh the benefits.[24] In our pilot study, we demonstrated that providing ED clinicians a child's estimated risk for appendicitis based on their current symptoms can reduce CT use.[25] The current proposal expands on our prior formative work by creating an electronic health record (EHR)-linked, web-based clinical decision support (CDS) system that integrates data from multiple sources and presents point-of-care CDS in order to augment clinician judgment and prioritize care delivery. Such a CDS system is likely to be an efficient and scalable method for translating evidence into practice across multiple general EDs, the primary sites where children with acute abdominal pain receive emergent care.

Community-based EDs in two regions will develop, implement, and prospectively evaluate a web-based CDS tool (Appy-CDS) for children with acute abdominal pain. Appy-CDS will identify pediatric patients with acute abdominal pain at increased risk for appendicitis, notify providers of a patient's estimated risk for appendicitis, and provide specific evidence-based recommendations. To measure the impact of Appy-CDS, we will conduct a cluster- randomized trial across 17 participating EDs in two large health systems with a combined total of more than 90,000 pediatric visits per year.

<u>Specific Aim 1</u>: To design and implement a triage-based trigger, as part of Appy-CDS, to identify pediatric patients with acute abdominal pain at increased risk for appendicitis.

- Hypothesis 1: Among pediatric patients with abdominal pain, a triage-based trigger will identify those at risk for appendicitis with a negative predictive value ≥99% and positive predictive value ≥20%.

<u>Specific Aim 2</u>: To evaluate the impact of the full Appy-CDS intervention on appropriate use of diagnostic imaging (CT and ultrasound) for pediatric patients with acute abdominal pain at risk for appendicitis.

- Hypothesis 2. Among eligible pediatric patients at risk for appendicitis, those treated at intervention EDs compared to those treated at control EDs will be less likely to undergo a CT examination.

- Hypothesis 3. Among eligible pediatric patients at risk for appendicitis, those treated at intervention EDs compared to those treated at control EDs will be less likely to undergo diagnostic imaging with CT or ultrasound.

Specific Aim 3: To evaluate the impact of the full Appy-CDS intervention on health care costs.

- Hypothesis 4. Among pediatric patients at risk for appendicitis, those treated at intervention EDs compared to those treated at control EDs will have significantly lower resource use in a defined 7-day period after an index ED visit.

In addition, we will monitor rates of appendiceal perforation, negative appendectomies, and missed appendicitis in our study cohort to ensure the safety of our proposed intervention. This innovative project builds on extensive previous work by our interdisciplinary team, including derivation and validation of pediatric ED clinical decision rules, CDS development and implementation, and economic and statistical analyses of ED resource use. The proposed point-of-care CDS tool will be a scalable model for using EHR-linked, web-based technology to safely deliver personalized and evidence-based care for children in the ED.

b) Rationale

Abdominal pain is one of the most common reasons for children and adolescents to seek care in the ED.[1] About 5%-10% of all pediatric ED visits are for abdominal pain, and appendicitis is ultimately diagnosed in up to 10% of these children.[2,3,26] Appendicitis is the most common surgical emergency in pediatrics. The lifetime risk of appendicitis is 8.6% for males and 6.7% for females.[27] When evaluating pediatric patients with acute abdominal pain, ED physicians often consider surgical conditions, such as ovarian torsion or cholecystitis, in addition to appendicitis; however, these conditions are uncommon in children.[28,29] In a recent multicenter study of more than 2,500 pediatric patients (3-18 years old) presenting to an ED with <96 hours of abdominal pain, 39% had appendicitis, while only 1.1% of patients had a surgical condition other than appendicitis.[21]

Despite the high incidence, diagnosing appendicitis remains challenging. The clinical presentation of non-surgical conditions causing acute abdominal pain (e.g., mesenteric adenitis, acute gastroenteritis) often mimics that of appendicitis. In addition, children may have difficulty communicating their complaints.[30] Up to 40% of children with appendicitis are misdiagnosed on initial presentation,[30-32] leading to delays in treatment and increased morbidity and mortality.[33] There is also considerable variability in the ED evaluation of children with suspected appendicitis.[5,19,34,35] A recent survey of 40 US children's hospitals demonstrated that, among children with appendicitis, CT use ranged from 21%-49%, and ultrasound rates ranged from 2%-26%.[5]

CT has been promoted as a method to improve diagnostic accuracy in patients with acute abdominal pain. In a landmark 1997 study, Rao et al demonstrated that CT lowered negative appendectomy rates in adult patients with acute abdominal pain.[36] Subsequently, Garcia Peña et al found CT to be highly accurate for identifying or ruling out appendicitis in pediatric patients with equivocal presentations.[37] Following these publications, a widespread increase in abdominal CT use was observed.[38,39] In some adult cohorts, increased CT use was associated with reductions in negative appendectomies.[40] Similar decreases in pediatric populations have not occurred.[16,18,35] In addition, increased CT use has not affected rates of appendiceal perforation in children or adults.[18-20,41,42] Thus, the benefits of CT for patients with equivocal

presentations have not been generalizable to larger cohorts of children with acute abdominal pain.

Overuse of CT is problematic given the risks of radiation exposure and increased costs of care. As a result of their smaller size and increased organ sensitivity, pediatric patients are especially vulnerable to radiation-induced injuries.[12,14,43,44] Researchers estimate that 1 radiation-induced malignancy may result from every 700 pediatric abdominal CT scans obtained in young males, and the risks may be even higher in young females.[13,14,45] This is especially troubling given the rapid increase in CT use nationwide.[17,35,39] Furthermore, for children who meet obvious clinical criteria for appendicitis, CT may delay operative care and increase costs.[11,16,41,46,47] For pediatric patients with low likelihood of appendicitis based on history and clinical exam, CT may prolong ED length of stay and increase costs.[10,11,18,48] Although CT has a demonstrated sensitivity of 94% (95% CI 92%-97%) and specificity of 95% (95% CI 94%-97%) for identifying appendicitis in children, its positive predictive value decreases substantially when used in children with low likelihood of appendicitis.[49] For a subset of pediatric patients with acute abdominal pain, the risks of CT may outweigh the benefits.

General or non-pediatric facilities are the ideal clinical setting to implement interventions for reducing CT use.[6,9] In the United States, most emergent care for children is provided in general EDs.[8] Only 7% of hospitals in the United States have EDs exclusively dedicated to pediatric care.[50] Recent data from the National Hospital Ambulatory Care Survey demonstrate that children with abdominal pain receiving care at general facilities were more likely than those at children's hospitals to undergo CT.[4,7,51] Plausible explanations for this difference include 1) general ED providers may have less confidence than pediatric ED providers in eliciting and interpreting physical exam findings in children and 2) general ED providers may be more familiar with adult guidelines that advocate CT to diagnose appendicitis.[52] Children undergoing CT at general institutions are also at risk of receiving a radiation dose that is excessive for their height and weight.[53]

Protocols to reduce reliance on abdominal CT in children have been safely developed and implemented in pediatric and general emergency departments.[21,54-58] In 2004, Garcia Peña and colleagues demonstrated the theoretical benefits of an appendicitis decision rule that first stratified patients based on their white blood cell count (WBC) and then applied a selective imaging protocol, with ultrasound as the first-line imaging.[59] Similarly, utilizing data from 10 pediatric EDs, our group has derived and validated a decision rule to identify patients at low risk of appendicitis using recursive partitioning. We found that the absolute neutrophil count (ANC) was the first node, or the most powerful tool, for identifying pediatric patients with acute abdominal pain who could forgo diagnostic imaging.[20,21] Additional key predictors were: focal right- sided pain, pain with walking, emesis, and duration of pain.[60] Several other pediatric and general EDs have implemented protocols that risk-stratify based on WBC and additional clinical variables.[55-58] Although there are no national guidelines regarding the management of children with acute abdominal pain, there is an increasing consensus among surgeons and ED providers on the use of risk stratification and the need to limit unnecessary CT exposure.[30,55,58] Collectively, single-site studies have documented that it is possible to safely reduce rates of advanced diagnostic imaging in patients with suspected appendicitis. To date, point-of-care appendicitis protocols that integrate laboratory data, symptoms, and clinical history have not been implemented and evaluated in multisite studies, likely due to logistical and practical barriers.

EHR-linked, Web-based CDS is a scalable model that has successfully changed clinician behavior and improved quality of care in a range of settings. Many CDS studies have been single-site efforts performed in academic medical centers.[61-65] A systematic review of research studies conducted through 2004

included 100 randomized and nonrandomized trials with concurrent controls, compared computer-based CDS with routine care. The authors noted that computer-based CDS improved clinician performance in 64% of the studies. Two of four ED-based trials showed that CDS led to improvements in diagnosis. In all clinical settings, improvement was noted more often with automatic delivery of CDS versus when users had to activate the CDS.[63] These results mirror our previous work demonstrating that CDS in outpatient clinics improves clinician adherence to diabetes guidelines and glucose and blood pressure control in diabetic patients.[66-68] In our previous work, we found that ED physicians are receptive to CDS based on validated prediction rules and are open to multiple means of incorporating CDS into the EHR.[69]

Implementing CDS via the EHR in multiple EDs presents unique challenges. EDs provide care to a high volume of acutely ill patients requiring rapid management decisions despite often incomplete clinical information. The proposed CDS will gather information from multiple sources at multiple times during the patient encounter, integrate these data using previously validated clinical decision rules, and provide evidence- based patient-specific clinical care recommendations in real time. Furthermore, ED clinicians work in dynamic and complex systems; each ED has evolved a particular method of decision-making or clinical workflow. For CDS to be successful, it is necessary to assess and account for local ED processes and clinician perceptions.[70] Given the current variability in ED management of pediatric patients with acute abdominal pain, local consensus for key recommendations in Appy-CDS is imperative. As in our previous work, we will conduct detailed workflow analysis, obtain key stakeholder feedback, seek endorsement from clinical leaders, and conduct pilot testing and iterative refinement of our CDS system.[71] Ultimately, the success of Appy-CDS will depend on its ability to save time, to provide accurate clinical care recommendations that augment clinician judgment, and to promote appropriate utilization of diagnostic imaging.[72]

CDS integrated into an existing EHR platform and delivered at the point of care is the ideal scalable intervention for reducing unnecessary CT use in children with acute abdominal pain.[73] In their 2007 article on US EDs, Gausche-Hill et al wrote, “Key opportunities for improving pediatric emergency care lie in non-children’s hospitals, where the vast majority of care occurs”.[8] The Appy-CDS intervention integrates clinical information to optimize management of children with acute abdominal pain in general EDs. Moreover, our technical approach is a useful template to generalize ED point-of-care CDS to a range of clinical conditions.

c) Innovation

The potential for computerized CDS to improve practitioner performance and adherence to guidelines is widely recognized. However, the impact of EHR-linked CDS on patient outcomes has been variable.[63,74,75] Review of previous negative studies identifies 3 principal reasons for failure: (a) Clinicians have not accessed the CDS information in a timely fashion, (b) the CDS has been limited to simple prompts or reminders, and (c) the CDS has not provided personalized information.[76,77] This project addresses these limitations by moving beyond simple computer prompts and reminders to provide more sophisticated, patient-specific recommendations. Furthermore, the CDS is integrated in the EHR at multiple points during the encounter, ensuring maximum visibility and allowing for varying practice styles and workflows in disparate EDs.

Previous CDS for pediatric emergency care have primarily been developed and implemented in academic children’s hospitals.[21,78,79] Because most pediatric ED visits and radiation exposures are in general EDs[9], there is an urgent need to study the effectiveness of pediatric-specific CDS in these settings.[6] We propose

to work closely with site-based leadership to develop and implement the Appy-CDS system in 2 large health systems with 14 general EDs. The EDs in this study vary in pediatric patient volume and geographic region. Thus, our intervention will be relevant to and more broadly generalizable to the settings in which most pediatric patients receive emergent care in the United States.[8]
The use of a Web service to host the CDS algorithms is a design feature that maximizes the scalability of the intervention, facilitates updating of algorithms as evidence or clinical practice changes, and maximizes the impact of CDS resources to improve quality of care for pediatric patients with acute abdominal pain. For instance, although magnetic resonance imaging (MRI) is currently not a standard diagnostic tool for pediatric patients with acute abdominal pain, our protocols could be modified to address use of this imaging modality in the future.

Innovation in Assigning Patients a Personalized Risk of Appendicitis: This project builds on previous successful efforts by our team in developing[71] and implementing CDS to support goal-based guidelines.[67] Previous CDS interventions for acute abdominal pain have largely presented clinicians with overarching, general evaluation options rather than patient-specific guidance.[55] A personalized risk assessment is more likely than a general recommendation to substantially change practice patterns.[80] The Appy-CDS will use available clinical data to estimate a quantifiable and patient-specific risk for appendicitis and prioritize the potential benefits of additional imaging modalities. Successful adoption of the proposed CDS for acute abdominal pain will facilitate development of additional CDS tools to enhance the care of children and adolescents in the ED.

An important aim of this study is to promote the appropriate use of diagnostic imaging. Due to variability in ultrasound performance,[81] protocols that simply recommend ultrasound for all patients with acute abdominal pain may have unintended consequences[82] and ultimately increase total imaging rates.[83] To avoid this, the Appy-CDS tool will provide detailed recommendations for when diagnostic imaging is not required as well as guidance on when CT may be more appropriate than ultrasound. By providing nuanced, evidence-based recommendations, Appy-CDS will offer guidance tailored to each patient.

d) Preliminary Studies

__Pilot data:__ **We recently completed a single-center pilot study to implement CDS integrated in an EHR for pediatric patients with acute abdominal pain.** For this project, based in the 2 primary EDs of Children's Hospitals and Clinics of Minnesota (Children's Hospital), we engaged a multidisciplinary team of radiologists, surgeons, and ED physicians to develop a consensus care pathway. Similar to the current proposal, goals of the pilot were to reduce CT use among pediatric patients with ED visits for acute abdominal pain. The pilot CDS stratified patients into 3 risk groups based on previously validated decision rules. First, we identified pediatric patients with acute abdominal pain at low risk for appendicitis who could safely forgo CT by applying our previously derived and validated decision rule.[20,21] Patients with an ANC $<6.75 \times 10^3/\mu L$, absence of nausea, and absence of maximal tenderness in the right lower quadrant were identified as at low risk for appendicitis.[21] Second, to determine which patients were at high risk for appendicitis and could proceed to the operating room without imaging, we used the externally validated Pediatric Appendicitis Score (PAS).[22,84] The remaining patients were classified as medium risk, and ultrasound was the recommended initial imaging modality. We then worked with our informatics group to create a simple "time-of-ordering alert": the pilot CDS tool was presented to clinicians when ordering a CT or ultrasound for patients with acute abdominal pain. The CDS tool was modified and refined in two phases. Drs. Schmeling and Mize, members of the Clinical Advisory Council for the current proposal, were instrumental to the success of the pilot.

Over the 36-month study period, 4,234 pediatric and adolescent patients with acute abdominal pain received care in the pilot EDs. Of these, 778 (18%) had appendicitis and 178 (23% of appendicitis cases) had a perforated appendix. After CDS was implemented, 942 (22%) had an abdominal CT, and 1,666 (39%) had an ultrasound. Interrupted time series design revealed a significant 23% decline in CT use post-implementation (OR=0.77 (95%CI.67-.88) P<0.01) (Figure 1). Safety outcomes remained unchanged over the study period.[54]

The pilot study provided information critical to the design of this project. Although the pilot reduced CT use, total imaging (CT or ultrasound) did not decrease. In addition, after a marked early post-implementation decrease in CT, further reductions were limited despite the Phase II refinements. Review of the intervention revealed 3 key themes we will address in the current proposal: a) clinicians prefer patient specific information to general approaches to acute abdominal pain b) clinicians do not want to calculate a patient's risk for appendicitis by entering multiple data points, and c) a "time-of-ordering alert" can promote replacement of CT with ultrasound but is not sufficient to reduce total diagnostic imaging. Based on the pilot data and our team's previous work, we conclude that the success of the proposed Appy-CDS intervention can be maximized by: (1) Making decision support available at the point of care and based on all available relevant clinical data; (2) Providing patient-specific information in a sophisticated manner, beyond simple prompts or reminders; (3) Integrating the CDS into the routine flow of patient care; and (4) Ensuring adequate ED staff training, process measurement, and feedback to reinforce use.

Figure 1. CT, US and Total Imaging Rates of Patients with Possible Appendicitis

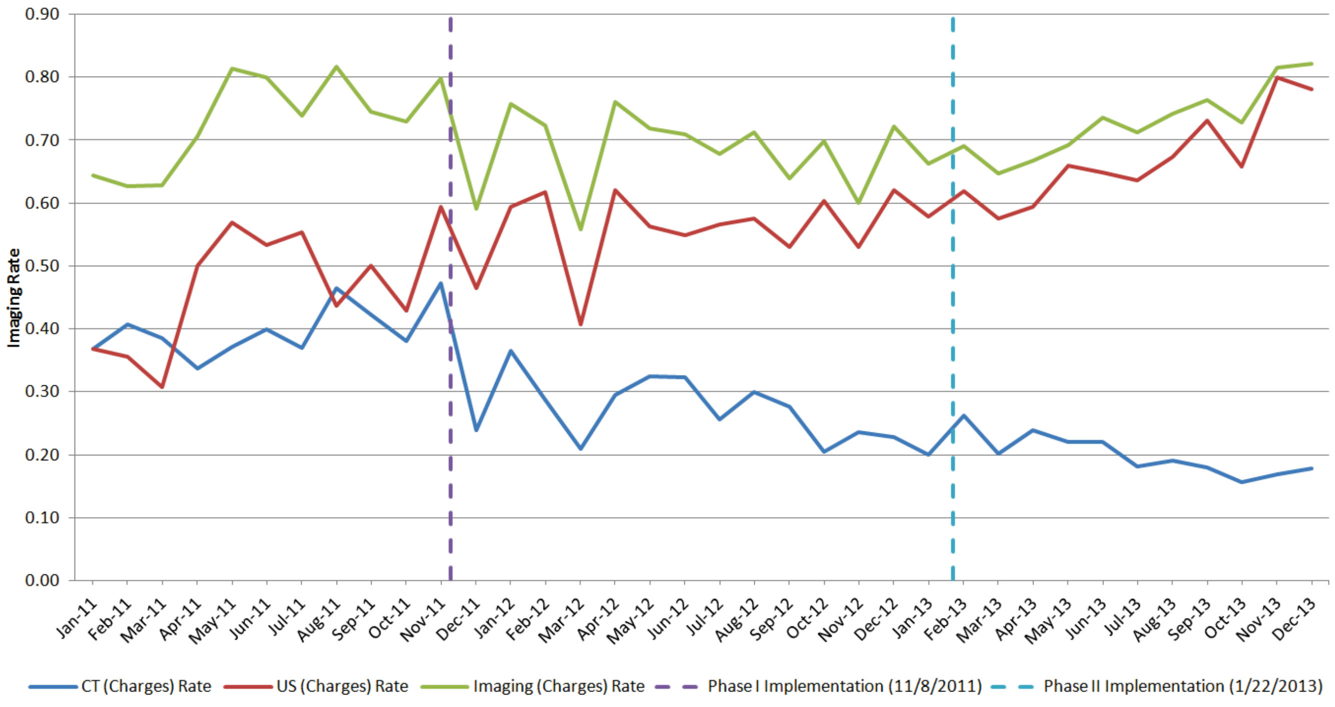

3) Study Sites and Population

We will conduct a prospective cluster-randomized clinical trial at 17 general EDs in two large care systems to evaluate the impact of Appy-CDS on CT and ultrasound use, health care outcomes, and direct medical expenditures. We will first measure ultrasound and CT use for 6 months pre-intervention in children identified at triage as at risk for appendicitis presenting for care in a participating ED. We will then group some sites with significant provider cross-coverage and pair within clusters by pediatric volume. We will then randomize half of the clusters to receive the full Appy-CDS. CT and ultrasound utilization in intervention and control sites will be measured for 24 months after the full Appy-CDS "go-live" date. Rates of perforation, negative appendectomies, and missed appendicitis will be monitored as safety outcomes. We will compare resource use and re-visit rates between intervention and control EDs within 7 days of the index ED visit. Figure 2 is an overview of the study design.

This study includes 17 general EDs operated by two health systems: HPMG (Regions, Hudson, Lakeview, Methodist, Westfield and Amery), in the Upper Midwest and KPNC (Sacramento, Roseville, South San Francisco, San Rafael, Santa Clara, South Sacramento, San Leandro, San Francisco, Fremont, Oakland and Richmond). These 17 EDs provide care to a diverse pediatric patient population (34% Hispanic, 19% African-American, and 11% Asian). None of the participating EDs are university-based; however, 5 EDs have academic affiliations. Most ED clinicians are board-eligible or board-certified emergency medicine physicians. Ultrasound is available at each participating ED, and 5 sites are referral centers for pediatric appendectomies. Each ED currently uses the EpiCare EHR (Verona, WI). Both health systems have agreed to participate and work with the study investigators to create linkages between their EHR and the Appy-CDS tool. *Collectively, the 17 participating EDs have more than 650,000 annual ED visits, of which 101,014 are patients aged 5-20 years).*

To be included, patients must: a) be 5-20.9 years old on the index date, and b) present to a participating ED with a chief complaint of acute abdominal pain.

a) Exclusion Criteria

Using data from the EHR and gathered from triage-based prompts introduced at all participating EDs, we will identify patients with a current pregnancy, previous abdominal surgery, trauma, cystic fibrosis, sickle cell anemia, inflammatory bowel disease, or other selected comorbidities at the prime site, or with an ED visit for abdominal pain in the prior 7 days. These patients will be excluded from the study cohort. As described below, based on responses to the initial screening questions, patients with >120 hours of abdominal pain or patients with no right sided or generalized abdominal pain will also be excluded from the risk score calculation and further analyses.

If patients are transferred to an outside facility (outside of KPNC or HealthPartners networks) for surgery *and* we are unable to review their operative and pathology notes, they will be excluded from the safety analyses. These patients will be retained in the primary analyses, evaluating the use of CT or US, with the diagnosis of appendicitis or not appendicitis assigned based on provider impression and imaging available at the time of ED discharge / transfer.

b) Patient Recruitment

Patients will be identified based on a chief complaint of abdominal pain and at HP, using automated alerts within the electronic health record (EHR). A flag will be created for patients who meet eligibility criteria. The flag will be stored in Epic for use by the full clinical decision support (CDS) at a later point. Although

the process of identifying eligible patients is fully automated, success of the study depends, to a large extent, on the engagement of nursing staff and providers at all participating sites. Enrollment can be maximized by using a combination of the methods outlined below.

1. Education of nurses, physicians and residents providing care in each emergency department (ED).
    a. Present the study at ED staff meetings.
    b. Train nurses and providers at shift meetings or other meetings as appropriate.
    c. Use the "train the trainer" approach including the recruitment of "champions" to promote the project.
2. Put in place a system to remind physicians about the EHR tools available to them.
3. Post laminated cards with project info next to all workstations as reminders to staff regarding the purpose and importance of responding to the EHR alerts.
4. Incentives to nurses or providers for completing the EHR-linked tools.
5. Provide feedback to nurses and providers regarding use rates.

4) Allocation of Interventions

EDs will be stratified by hospital system and then pair-matched based on pediatric patient volume. Matching EDs on selected characteristics *a priori* minimizes possible sources of bias by balancing potential ED-level confounders between intervention arms. It will sharpen our comparisons of interest and it is recommended for trials with a small number of clusters[85,86] Further, this design allows staggered implementation without breaking concealment to better control for seasonal variation in ED use.[87-89] Matched EDs will be randomly assigned, in pairs, to one of the two arms using a block design. The randomization scheme will be prepared by the study statistician (GVB) using a computerized random-number generator. Assignment will be revealed before implementation. Emergency Department stakeholders at each site have agreed to randomization before initiation of the study.

5) Description of Intervention

Appy-CDS will be developed using existing CDS platforms at each site. The tool will be consistent but also locally relevant, thus will have subtle differences in design and workflow. Implementation and training will be tailored to local preferences.

a) Design at HealthPartners

At all HealthPartners (HP) sites, the Appy CDS has 3 components. The first, *Triage CDS,* will be implemented at triage at all participating EDs. It will leverage learnings from previous studies and identify our population of interest. The second, *MD Alert*, will be triggered at initial provider login to the EHR at the intervention sites and will cognitively prime[90-93] the ED provider toward use of Appy-CDS. The third, *Imaging CDS*, will be triggered at intervention sites when a clinician begins to order a CT or ultrasound and will present a risk estimate and targeted recommendations. Each component is described in detail below. Final algorithms will be modified based on evaluation of clinical workflow and feedback from ED, radiology, and surgery clinical leaders. Detailed information of HP specific workflow is illustrated in Appendix A.

<u>*Triage CDS:*</u> When a patient 5-20.9 years old presents to a participating ED with a chief complaint of

abdominal pain, an alert will trigger prompting the triage nurse (or provider at KPNC) to answer several discrete historical questions. Relevant previous diagnoses will also be pulled from the EHR (e.g., sickle cell anemia, inflammatory bowel disease, cystic fibrosis, previous abdominal surgeries) along with the results of a urine pregnancy test, if obtained. Thus *Triage CDS* will consistently apply exclusion and inclusion criteria across all sites to identify a subject group, in real-time, with elevated appendicitis risk (Hy 1). This group will then be targeted by the full Appy-CDS system at intervention sites and will serve as the comparison group at control sites (Hy 2-3).

MD Alert: At intervention sites, a provider EHR- based alert will trigger for patients identified by the *triage CDS* as at risk for appendicitis. This alert will provide a recommendation that, if appendicitis is being considered, to "order a CBC and refer to the risk-stratification algorithm" (accessible via embedded hyperlink).

Imaging CDS: At the intervention sites, when a clinician begins ordering a CT or ultrasound for a study-eligible pediatric patient, an alert box will prompt the clinician to answer 3 discrete questions about the patient's history and physical exam findings. This information will be matched to the data provided by the *Triage CDS*, processed through a predefined algorithm, and presented to the clinician in real time as estimated risk of appendicitis. As described in detail below, a risk estimate will be developed based on data from our previous multicenter study.[21] Along with this estimate, we will provide tailored clinical recommendations on the need for any additional diagnostic testing. For example, if the WBC count is available, it will be used to calculate a risk of appendicitis. If the WBC result is pending or has not been ordered, a risk for appendicitis will be provided for a range of WBC levels. Ultimately, clinicians will be able to order any imaging study and provide additional care at their discretion. The imaging CDS will not trigger if a clinician does not attempt to order a CT or ultrasound. Lastly, the Appy-CDS will allow clinicians to activate a hyperlink to an external Web platform, where they can obtain additional information about appendicitis, previously validated prediction rules,[24] and risks associated with CT (See Appendix A for design and workflow).
At all KPNC sites, pediatric patients with abdominal pain will be identified based on their chief complaint. Providers will be trained to complete additional appendicitis assessment questions within RISTRA, KPs existing platform linked within the EHR. Analogous to the HP sites, these questions will be used to assess eligibility and appendicitis risk.

b) Design at Kaiser Permanente

At KPNC intervention sites only, users will again be trained to use the RISTRA platform to access the CDS. There will be an interface where providers will be asked 3-5 additional questions regarding the patient's signs or symptoms. The patient's individual appendicitis risk based on elicited questions and information recorded in the EHR will then be presented. Based on this risk score, there will be targeted recommendations for the provider to consider in their diagnostic evaluation. In addition, there will be a time of order BPA for Ultrasound and CT, again reminding providers to consider the appendicitis risk score. Figure 2 shows a high level schematic of the Appy-CDS across sites. Detailed information of KP specific workflow is illustrated in Appendix B.

Figure 2. High Level Schematic of Appy-CDS System

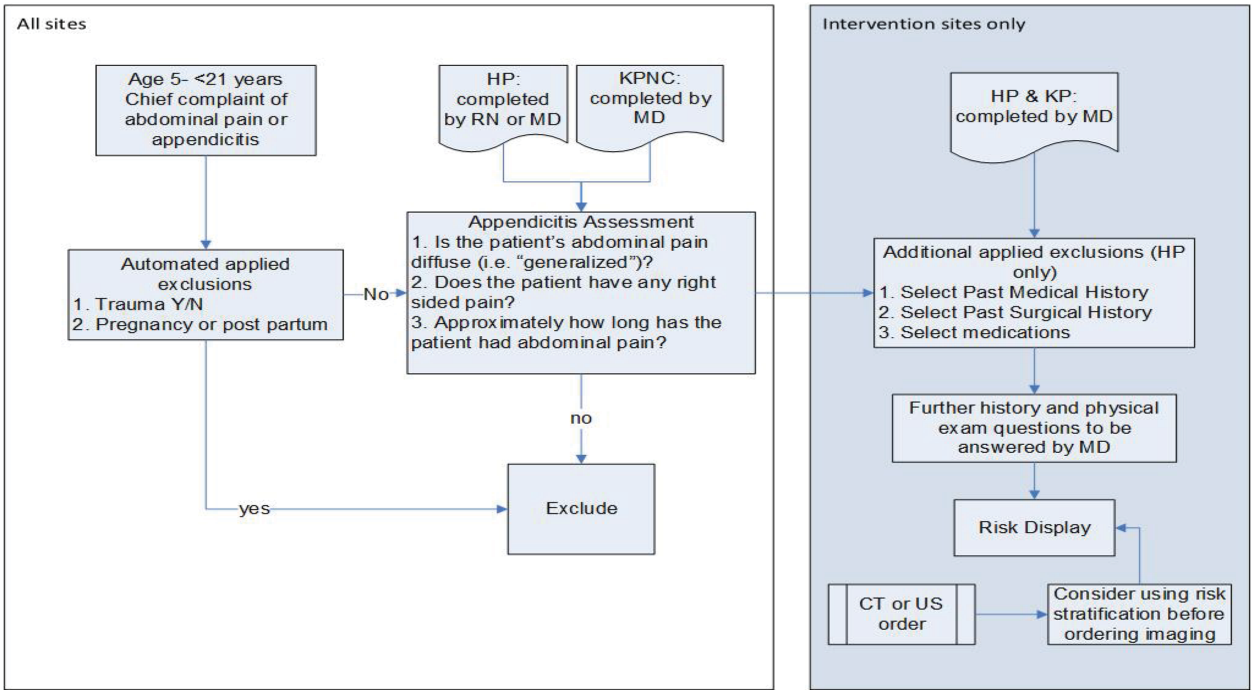

c) Implementation of the Appy-CDS Intervention

At intervention sites, the Appy-CDS system will provide patient-specific targeted recommendations to clinicians during distinct phases of the clinical encounter to maximize visibility and usability. Furthermore, Appy-CDS is designed to address several common reasons identified by Ebell,[80] Cabana,[94] and Kawamoto[95] for clinician non-adherence with CDS tools.

To address lack of agreement with clinical recommendations, we will convene a Clinical Advisory Council consisting of ED clinicians, ED nurses, site PIs, nurse managers, and medical group IT experts from the participating health systems, who will meet quarterly via teleconference to review, refine, and approve the Appy-CDS system. Dr. David Schmeling, Chief of Pediatric Surgery and Dr. William Mize, Chief of Pediatric Radiology at Children's Hospital and Clinics of Minnesota, have agreed to serve on the Clinical Advisory Council. We will conduct detailed clinical workflow assessments at each participating ED to ensure proper integration. We will seek letters of agreement from the directors at participating EDs indicating their support for the final Appy-CDS system. A list of Advisory Board members from each site is listed in Tables 12 and 13.

To address lack of awareness and lack of familiarity with the CDS system, Physician and Nurse Champions (depending on site) will be recruited from each participating ED. They will receive extensive training from site PIs and project managers in the Appy-CDS system. Before implementation of the *Triage CDS*, Physician Champions will orient nurses regarding the interactive alerts in the triage box. At the intervention sites, before implementation of the full Appy-CDS, Physician Champions will orient all clinicians to the *MD alert and Imaging CDS.* The site-based Physician Champions will also be available to

field questions and reinforce CDS use at the site.

To address low outcomes expectancy, site PIs will provide summary reports to Physician and or Nurse Champions, who will disseminate them to ED clinicians at intervention sites. Reports will include the number of patients with acute abdominal pain evaluated, frequency of use of Appy-CDS, and ED return visits. This feedback will promote continued use over the 24-month intervention. At Kaiser, as in previous interventions,[67,68] we will use small *group or individual incentives ($5 gift cards)* to facilitate ED clinicians' acceptance of new EHR-based tools. HealthPartners will also use small incentives including offering gift cards for using the CDS and providing food to individual groups (e.g. residents and physician assistants) if a pre-specified threshold for CDS use is reached.

The Appy-CDS intervention will be implemented over 4 phases, as described below.

At Control and Intervention Sites

Phase 1: The HP and KPNC Clinical Advisory Councils will review and approve the Appy-CDS system. The algorithms underlying the CDS will reside on a distinct Web site housed on secure servers owned by each health system. Site PIs will work with their IT personnel to integrate the triage CDS component with their EpiCare platform.

Phase 2: We will first implement *Triage CDS* to screen patients and create a cohort at increased risk for appendicitis. Once the local IT groups have completed the programming necessary, we will pilot the *Triage CDS*. Triage nurses will be asked to provide feedback on their experience with the *Triage CDS*, including assessment of the clinical plausibility, utility of prompts, and impact on clinic workflow.

At Intervention Sites Only

Phase 3: Once modifications to the *Triage CDS* are complete and the triage tool fully implemented, we will begin to develop and test the *MD Alert* and the *Imaging CDS* components. The clinician study Investigators will test the functionality of the full Appy-CDS using a series of simulated patients meeting eligibility criteria. After the first round of testing and modifications, we will pilot-test the *MD Alert and Imaging CDS* components at intervention EDs in each hospital system for up to 3 months. During this time, we will seek feedback from clinicians and nurses to ensure proper integration of Appy-CDS into the ED clinical workflow. To ensure proper function in each hospital system, Appy-CDS will be implemented over 1 month in the intervention EDs at each hospital system. Rollout to the entire clinical staff and formal training will be accomplished using strategies similar to our previous work, including group and individual meetings with all ED physicians, triage nurses, clinic staff, and email reminders. Physician Champions from each site will assist with training.

Phase 4: Ongoing education is critical to ensure continued use of the CDS throughout the 24-month intervention. Following implementation, ED clinical supervisors and staff will receive email reminders with rates of use of the triage alert (at HP), and the CDS. During the initial phase of provider education, adoption of the new practice pattern will be encouraged through the use of incentives. The project manager or Physician Champion will meet with triage nurses and ED clinicians throughout the implementation period to provide information on use of Appy-CDS and gather informal feedback. We will conduct a second round of staff training on the CDS after the first implementation year.

6) Plan of Analysis and Analytic Models

a) Development of a Risk-Prediction Estimate for use in Appy-CDS

We derived a new pediatric Appendicitis Risk Calculator (pARC) from an existing, de-identified cohort of children with suspected appendicitis.[21] Although the parent study included children 3-18.9 years, given the low risk for appendicitis in children under 5 and increased likelihood of atypical clinical presentations,[96] the pARC score was derived and validated in the subset of children and adolescents 5-18.9 years. In the prior prospective study, conducted from March 2009 through April 2010, ED clinicians collected clinical data from patients with suspected appendicitis at 9 pediatric emergency departments (PEDs). Treating clinicians enrolled children and adolescents who presented to the PED with <96 hours of abdominal pain and under evaluation for suspected appendicitis. "Suspected appendicitis" was defined as undergoing laboratory testing, diagnostic imaging or a surgical consultation for possible appendicitis. Patients with the following conditions were excluded: pregnancy, prior abdominal surgery, inflammatory bowel disease, chronic pancreatitis, sickle cell anemia, cystic fibrosis, a medical condition affecting the ability to obtain an accurate history, or history of abdominal trauma within the prior 7 days. Study procedures related to training of site staff, patient enrollment, data collection, and data management have been described previously.[21]

We validated pARC using de-identified data from two independent cohorts of patients 5-18 years with visits to the Children's Hospital Boston PED from 2003– 2004 and from 2013 – 2015. These cohorts were chosen as the validation sample as their criteria for cohort entry, data collection, data cleaning and data quality control were similar to those used in the derivation sample. Consistent with the recommendations of Altman, the validation population did not overlap with the derivation population.[97] Clinical data were collected as part of distinct research[20] and quality improvement projects. For both, children and adolescents with possible appendicitis were prospectively identified by trained coordinators who screened patients in the PED 10 hours per day. Subjects were included in the cohort when their treating emergency physician ordered advanced imaging or a surgical consult with concern for appendicitis.

In both the derivation and validation cohorts, the primary outcome was appendicitis. Patient history, physical exam, and laboratory variables were collected and prospectively recorded using standardized processes in the parent studies.[20,21] For consideration in pARC, we included only predictors with less than 10% missing data and at least moderate inter-rater reliability (kappa >.35).[60] Following the prognostic model development approach recommend by Royston et al[98], we selected all potential predictors for the multivariable model based on the following rules: 1) variables associated with appendicitis with a p-value <0.05 in the age and gender adjusted models; 2) associations between variables and appendicitis were in the expected direction; 3) for binary predictors, the beta coefficient was greater than 0.4; 4) Transformation of the laboratory values to a normal scale and shape of the association was informed by graphical exploration; 5) If only WBC was available, but not ANC, the ANC was imputed as ANC = (-.8783 + 1.1008 * sqrt(WBC))^2; 6) If neither WBC nor ANC were available, the ANC was imputed as $7x10^3/\mu L$, corresponding to the mean ANC in our derivation cohort; 7) Interactions between age and gender and each additional predictor with appendicitis were evaluated as potential terms in the model. The final pARC model is shown below.

Table. pARC risk score equation from logistic regression analysis

	Final model	p-value
Clinical characteristic	Beta Coefficient (95% CI)	
Intercept	-8.7	NA
Male	1.28 (.89, 1.66)	<.0001
Age and Gender		
Age 5-7.9 years	0.38 (-0.04, 0.80)	.08
Male 5-7.9 years	-1.05 (-1.65, -0.44)	.001
Age 8-13.9 years	-0.72 (-1.21, -0.23)	.004
Male 14-18 yrs / Female 12-18 yrs	Reference	
Duration of pain in hours		
<24	Reference	
24-<48	0.47 (0.22, 0.72)	.001
48-96	0.10 (-0.18, 0.38)	0.49
Presence of pain with walking	1.05 (.80, 1.30)	<.0001
History of Migration of pain to RLQ	0.46 (0.24, 0.67)	<.0001
Maximal tenderness in RLQ	1.14 (0.89, 1.40)	<.0001
Abdominal Guarding	0.67 (0.46, 0.89)	<.0001
For ANC<14 $x10^3/\mu L$, $\sqrt{ANC}$	1.77 (1.56, 1.99)	<.0001
For ANC≥14 $x10^3/\mu L$, constant	6.62 (5.94, 7.29)	<.0001

RLQ: right lower quadrant; ANC: absolute neutrophil count; final model: C-statistic: 0.864

The newly derived pARC was then applied to the validation cohort. We evaluated the discriminatory performance using the area under the curve (AUC) plot and AUC statistic.

Complete data for the validation of pARC were available for 1426 patients. Across seven clinically actionable risk categories (< 5%, 5-14%, 15-24%, 25-49%, 50-74%, 75-84% and ≥ 85%) the pARC score provided valid risk prediction. The AUC for pARC was 0.85 (95% CI 0.83-0.87).[99]

Hypothesis 1: Among pediatric patients with abdominal pain, a triage-based trigger will identify those at risk for appendicitis with a negative predictive value ≥99% and positive predictive value ≥20%.

The purpose of the *Triage CDS* (triage-based trigger) is to reliably identify a study cohort at elevated risk of appendicitis at both the intervention and control EDs. This cohort will comprise the analytic sample used to determine the impact of the Appy-CDS system on our key outcomes of CT and ultrasound use (Hy 2 & 3) and health care costs (Hy 4). In addition, data from *Triage CDS* will be included in the appendicitis risk algorithm (as detailed above). Final content of *Triage CDS* will be based on our previous decision rule,[21] expert opinion, and review of ED workflow; Appendix A demonstrates the questions that are included.

Assessment of the *Triage CDS* will consist of 2 steps. First, the frequency that triage nurses or providers answer questions when prompted in the EHR (pain duration <96 hours, pain on right side, history of emesis, pain with walking) will be identified. Second, among pediatric patients with complete data in the EHR, the negative and positive predictive value of the triage tool will be determined. We hypothesize that among pediatric patients with complete data, the triage tool will yield a positive

predictive value of at least 20% and a negative predictive value of at least 99%. Due to variation in design, patient load, and number of urgent pediatric presentations, we anticipate considerable differences in data capture across EDs. Identifying these differences will inform optimal site-specific system design and training. If the positive or negative predictive value deviates substantially from our goals, (i.e., a 95% confidence interval for negative predictive value failing to include 99%), before full implementation we will review EHR data for all appendicitis cases and refine our trigger. Following implementation, we will prospectively track the performance of the *Triage CDS* at each site and report performance metrics (sensitivity, specificity, positive predictive value, and negative predictive value). If significant differences are discovered across study sites, we will use site-specific estimates of negative and positive predictive values as covariates in multivariate analysis of our key study outcomes (Hy 2-4).

Hypotheses 2 and 3: Appy-CDS implementation will reduce CT and total diagnostic imaging (CT and ultrasound) in patients identified as at risk for appendicitis.

Effectiveness of the full Appy-CDS system will be evaluated using a cluster-randomized trial. We will include data from both the pre and post intervention periods in the analysis to account for site-specific variation and potential imbalances in baseline imaging use. Our analysis will follow an intent-to-treat approach and consider both randomization and the timing of the Appy-CDS implementation at the particular study site. Patient and ED- level confounding will be addressed in three ways. First, our paired randomization scheme will attempt to balance potential confounders across study arms before Appy-CDS implementation. Second, we will use a pre- post intervention design to account for imbalances at baseline in imaging use. Third, before final analysis, we will compare patient characteristics across study arms and randomized pairs. If ED or patient-level characteristics deviate in the control and intervention arms, they will be adjusted for in multivariable models.

To test the effectiveness of the full Appy-CDS system, we will compare ratios of CT and ultrasound use pre and post implementation across study arms (i.e., difference-in-differences approach). This is best described as estimating a time (pre/post intervention) by treatment interaction. We will conclude that the Appy-CDS changed CT or other imaging and/or ultrasound utilization if significance at the 5% level is found using a 2-tailed test. To best estimate this effect across the entire pediatric ED patient population, we will fit a marginal model that provides population-level impacts averaged across all clusters.[86] To do this, generalized estimating equations (GEE)[100] and a log-linear model with an autoregressive covariance structure will be employed. A parsimonious approach to multivariable adjustment will be followed, and only factors found unbalanced across study arms at the 5% level and associated with the effect size (>.2) will be retained and included in the models as covariates.

Exploratory Analysis

Effectiveness of the intervention may vary by population subgroup and provider. Although our study may be underpowered to detect such differences, we intend to conduct two exploratory analyses. The first will examine if patient and provider characteristics act as moderators of the overall intervention effect, examining both single predictors, such as patient age, sex, and risk of appendicitis and a composite moderator.[101] Our second exploratory analysis will focus on the process of provider change, comparing trajectories of imaging and ultrasound use across intervention sites.

Hypothesis 4 – resource utilization: The skewed nature of health care utilization patterns is well known.[102,103] This is particularly true of episodic hospital data such as the treatment of appendicitis which

has a known likelihood of complications.[104] We will follow the standard outlined in the literature and model the total amount of emergency and hospital-based care used during the treatment episode and the likelihood of re-visits due to misdiagnosis or surgical complications. This will begin by modeling the likelihood of re-visits due to an adverse event (e.g., wound infection, abdominal abscess, and obstruction) or other unplanned re-admission using logistic regression. Then we will directly model the total costs of the treatment episode. The choice of a statistical approach to model episode costs will depend on the observed distribution of these costs. The most common approach is the log-linear model. However, this model requires the use of a retransformation factor that is sensitive to heteroscedasticity.[102] Thus, we will explore the use of other specifications such as the gamma distribution and compare alternatives using a modified Hosmer-Lemeshow test. To facilitate this approach, an alternative hierarchical estimation technique, GEE, may be considered. GEE extends the generalized linear model to allow for covariance matrices that specify clustering of the data (e.g. at the ED level).

We anticipate some variation in billing practices across study sites and variation in patterns of care. Thus, we will examine health care utilization from two perspectives, distinguished by the dataset. The first analytic dataset will include the entire sample and will focus on ED and other hospital costs incurred at study sites. This analysis will focus upon the impact of the Appy-CDS from a single hospital perspective. The second analytic dataset will be limited to those insured by HealthPartners and KPNC. This tightened sample will provide complete capture of all billed and reimbursed medical care and accurately estimate the risks of ED re-visit and total episode costs and examine the Appy-CDS' impact across the broader health care system.

Similarly, two utilization measures will be employed. The first will be Total Cost Relative Resource Value units (TCRRVs), which are endorsed by the National Quality Form (NQF)[105] to measure costs for the full study cohort. TCRRVs are based on Centers for Medicare and Medicaid Services (CMS) relative value units (RVUs) but extend RVU measures to include inpatient, outpatient surgery, ED services, and scheduled outpatient, professional, and pharmacy services that do not have CMS RVU weights. Because TCRRVs value services similarly to RVUs, TCRRVs are convertible to dollars using the Medicare cost factor and have been used by our team in several recent econometric studies.[106-109] The second measure will be billed amounts, or the total dollars billed, including patient co-pays and deductibles. The use of billed amounts allows comparison between hospitals in HPMG (open access) and KPNC (total cost per member) systems. Although we do not anticipate significant differences in adverse event rates post Appy-CDS implementation if these do occur, we will determine the long term health care cost and quality of life impact by developing a simulation model of the Appy-CDS implementation using established techniques.[106,110-117]

Table 1. Power estimates*

Number of patients enrolled per ED per year	Decrease in imaging 25%	Decrease in imaging 20%
**200**	80	63
**300**	91	82
**400**	98	86

*Assumptions: Number of sites: 14
Intra-class correlation =.05
Baseline CT Use Rate = .3

Sample size and power

We present in Table 1 results from our *a priori* power analyses to evaluate the impact of the full implementation of Appy-CDS (Hy 2). Power estimates are based on 14 clinical sites. We anticipate 15 sites will be available, 8 intervention and 7 control sites. Over the 36-month study period, we anticipate 24,603 patients aged 5-20.9 will present with acute abdominal pain, of whom 2,634 (11%) will have appendicitis. Based on our previous work and review of other prediction rules, we anticipate that the Triage CDS will identify about 50% of patients who present to the ED with acute abdominal pain as being "at risk for appendicitis". Thus, the

total number of patients at risk for appendicitis, and whose outcomes we will evaluate across the 14 EDs, is forecasted to be 4,200 per year (or on average, 300 patients per ED). Based on preliminary data from the participating EDs, we estimate that the baseline utilization of CT is 30%. We anticipate that the group of ED-level covariates and the temporal correlation with the error structure will explain a significant proportion of variance of the dependent variable. However, power may be overestimated because sample sizes will vary by ED.[118] Our power analyses consider a range for possible decreases in imaging rates and sample sizes while fixing a high intra-class correlation. Here, we report the estimated power to detect a significant period intervention effect, assuming a 2-tailed test with alpha set to 0.05. Actual power of our intervention may vary is enrollment goals are not reached.

Missing data

Missing data on predictors of appendicitis and use of diagnostic imaging in patients identified at risk of appendicitis will be handled using multiple imputation techniques applying a regression switching with predictive mean matching (MICE-PMM).[119] Patient characteristics, implementation variables, and provider characteristics will be used to maximize information used for multiple imputations.

b) Measurement of Outcomes

Progress of all eligible patients before and during implementation will be followed for all outcomes.

Identification of a cohort of patients with acute abdominal pain at risk for appendicitis (Hypothesis 1): Using existing EHR data and simple prompts implemented at triage at all 17 sites over the 6-month pre-intervention period and 24-month trial we will identify our study cohort of children and adolescents at elevated risk for appendicitis. The algorithm underlying this tool will exclude patients with chronic abdominal pain, abdominal trauma, and/or pregnancy, because their management is beyond the scope of the proposed intervention. For patients undergoing an appendectomy within 7 days of their index ED visit, a trained, blinded nurse abstractor will review their pathology report. For patients undergoing appendectomy, appendicitis is defined by specific terminology on the pathology report, as in our previous multicenter study (consistent with appendicitis, neutrophil invasion of tissue, serosal inflammation, acute peri-appendicitis, or acute appendicitis).[21] For patients with perforation undergoing drainage and or intravenous antibiotics and interval appendectomy, diagnosis will be based on clinical notes from the treating surgeon. For patients transferred from the ED where they initially sought care to a facility with pediatric surgical expertise, we will seek final diagnoses and pathology reports from the receiving institution. If we are unable to verify final diagnosis, the patient will be excluded from primary analyses. These patients may be included sensitivity analyses with the diagnosis of appendicitis or not appendicitis assigned based on provider impression and imaging available at the time of ED discharge / transfer. For HPMG, outside of the study EDs, Children's Hospital would be the primary outside institution to receive transfers for further evaluation and appendectomy. Thus, we will establish a registry of patients with a discharge diagnosis of appendicitis from Children's Hospital and use a probabilistic method to determine whether these patients are matches with patients receiving care in the HPMG study EDs. KPNC uses a hub and spoke system for pediatric surgery. Among the 11 CREST sites are four pediatric surgical hubs.

Reduction in CT use (Hypothesis 2): Among pediatric patients identified through the Appy-CDS as at risk for appendicitis, receipt of an abdominal and/or pelvic CT during the index ED visit will be determined through completed orders in Epic. We will identify CTs obtained using the following CPT codes: 74150, 74160, 74170, 74174, 74176, 74177, 72192, 72193, and 72194. Only CTs ordered in the ED will be included.

Total Imaging Utilization (Hypothesis 3): Among pediatric patients identified through the Appy-CDS as at risk for appendicitis, receipt of an abdominal and/or pelvic CT or abdominal and/or pelvic ultrasound during the index ED visit will be determined through completed orders in Epic. We will identify CTs as described above and ultrasounds using the following CPT codes: 76705, 76700, 76770, 76775, 76857, 76830, and 76856. Only ultrasounds ordered in the ED will be included.

Direct medical expenditures (Hypothesis 4): For HP and KPNC members, utilization of all medical services within 7 days of the index ED visit will be captured from insurance claims data. Hospital-based costs at the study sites for nonmembers will be assessed using Total Care Relative Resource Value units (TCRRVs),[120] described in detail below. While the goals of the CDS are to reduce CT use, the CDS may inadvertently lead to overuse of other diagnostic tests. In addition, by discouraging CT, the CDS may result in prolonged ED or inpatient observation. To capture all potential costs related to the treatment episode, we will include all utilization of health care services occurring at the ED visit and during the subsequent 7 days. This time window is adequate to include the most common complications associated with an appendiceal perforation. Across both participating health systems, coding guidelines and incentives ensure that all services provided should be accurate in the claims data. Although we do not anticipate differences in appendectomy or adverse event rates related to initiation of the Appy-CDS, if this does occur, we will assess the potential long- term impact of these differences by modeling long-term impact over a 12- and 24-month timeframe.[106,110-117,121]

Appendiceal perforation, negative appendectomies, and missed appendicitis (safety outcomes): Although it is unlikely that the Appy-CDS will significantly increase rates of adverse events, the following clinical or safety outcomes will be monitored both before and during the full Appy-CDS intervention. *Appendiceal perforation* will be evaluated in patients who undergo appendectomy based on chart review of the surgeon's intra-operative note. As in our previous work, perforation will be defined by keywords in the operative report (presence of abscess, peritonitis, complex appendicitis, or purulent material). In addition, we will review all charts for patients with a discharge diagnosis of perforated appendicitis (ICD-9 540.1) who do not undergo an appendectomy during their index hospitalization, because a subset of patients with appendiceal perforation (4% in our previous multicenter cohort, unpublished data) will be admitted for intravenous antibiotics and undergo an interval appendectomy 6-12 months later. *Negative appendectomies* are appendectomies in which the pathology report reveals no evidence of appendiceal inflammation. This will be determined via review of pathology reports. *Missed appendicitis* is when a patient presents for the evaluation of abdominal pain, is discharged home, and returns within 1 week with appendicitis. Missed appendicitis will be identified through review of automated EHR data for all patients with appendicitis. As described above, under exclusion criteria, patients who are transferred for definitive care and whose final pathology and surgical reports are not available will not be included in the assessment of safety outcomes.

Appy CDS Project Consort Diagram- Draft

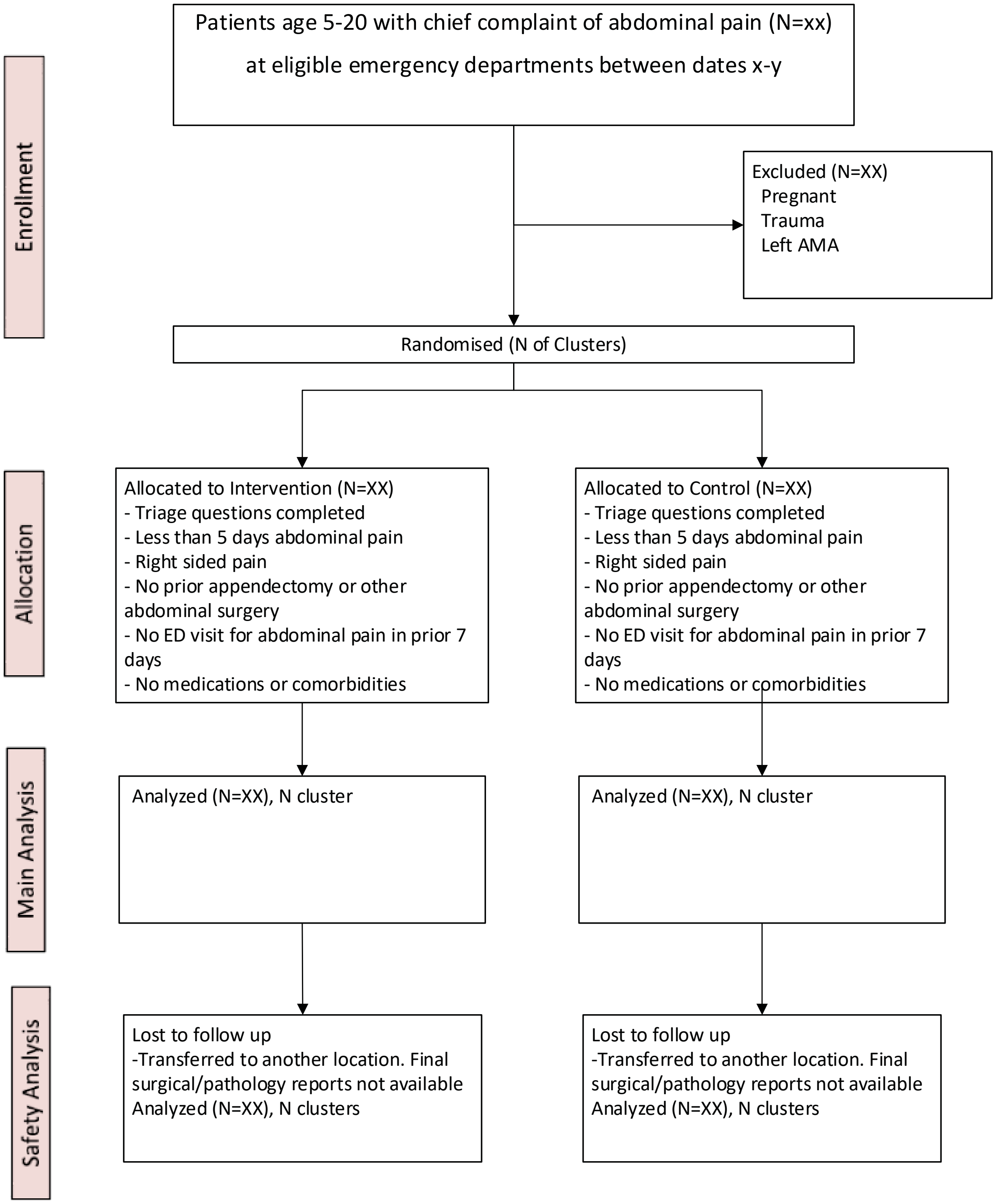

7) Reporting and Data Storage

a) Capturing Transfer Patients

One of the unique challenges of this project at the prime site is that many children in this age group are transferred to another hospital for additional follow up or surgery. A subset of patients presenting to a HealthPartners or HealthPartners affiliated ED who are eligible for Appy CDS will most likely be transferred to Children's Hospitals of Minnesota for definitive surgical care. In order to evaluate final diagnoses for these patients, a process for identifying and linking patients has been developed and is outlined in Figure 3.

Figure 3. Process for Identifying and Linking Transferred Patients

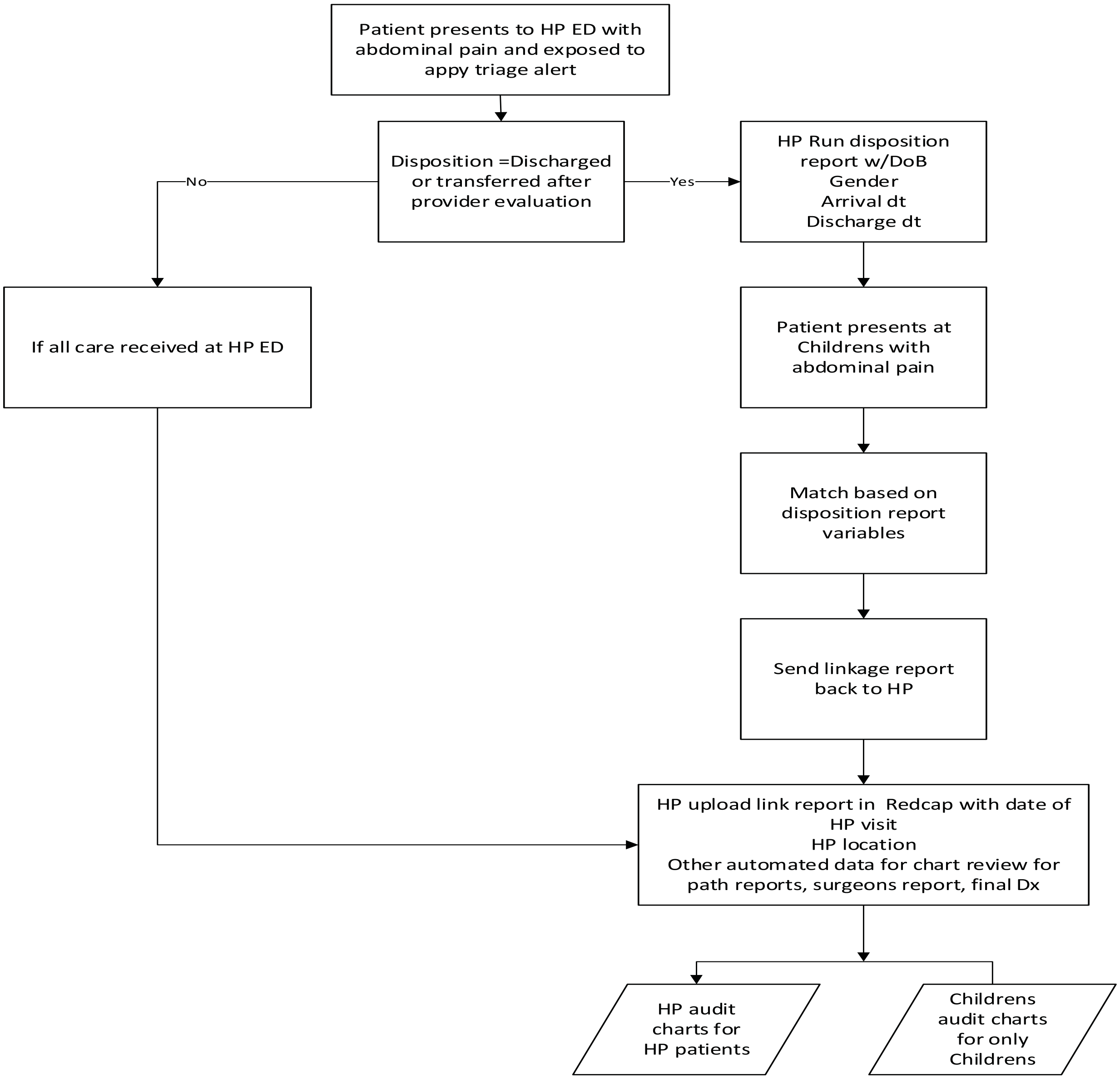

Data transfers will occur on a monthly basis. The limited data set sent from HP to Children's will include a study ID, date of birth, sex, arrival date, discharge date, reason for encounter, disposition, and discharge disposition of patients that were transferred from a HealthPartners facility to Children's. Files will be sent through secure file transfer and password protected. The above figure illustrates the process for capturing transfer patients and evaluating final outcomes.

In other cases, if patients are transferred out of the HP or KP system for surgery and we are unable to review the operative and pathology notes we will exclude them from the primary analyses because we cannot confirm appendicitis based on our definition. In sensitivity analyses we will explore inclusion of these patients with classification of final diagnosis (appendicitis versus other surgical or medical condition) based on diagnosis at the time of transfer.

b) Ongoing reporting

Study enrollment and use of the CDS will be monitored and reported on a weekly and/or monthly basis using automated data. Use reports will be shared with ED leaders and staff to encourage ongoing use of the tool. This approach has proved successful in previous projects.

Internal reporting, to promote continued CDS use, will vary by site. At HealthPartners, we will report responses to the Triage BPA for the 6 participating sites and risk score calculation for the intervention sites, on a monthly basis, with additional weekly reporting as needed.

Examples of Use Reports:

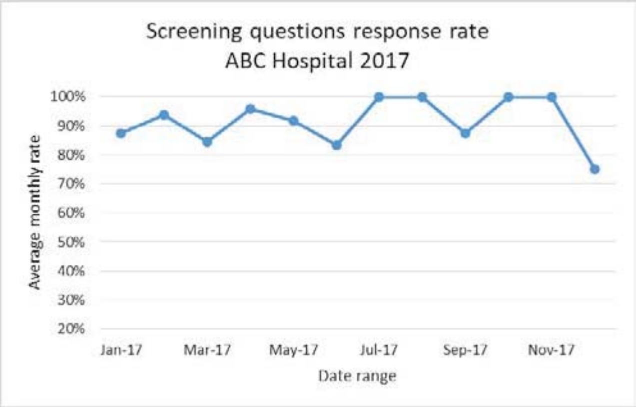

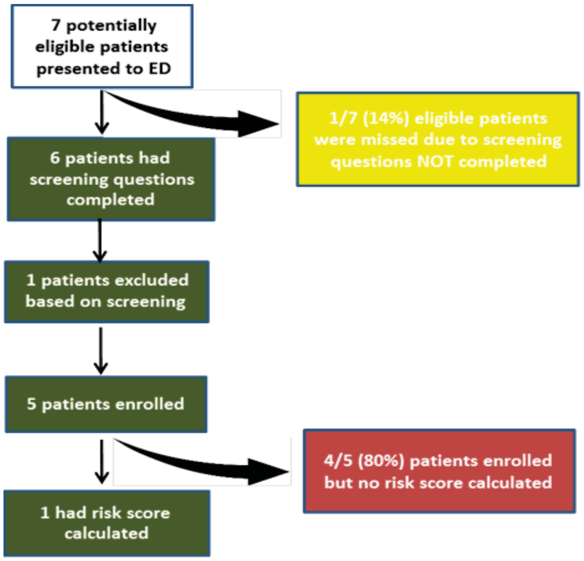

Enrollment reporting, by Cluster and health system

*Column A: Starting with cc of abdominal pain and excluding; left prior to being seen, had prior ED visit, trauma, pregnant, chronic condition/medication, or prior appendectomy, and for HP only BPA did not fire

**Column B: Screening questions not completed includes 1) nurse not completing BPA questions at HP, provider not completing questions via BPA or CDS at HP, provider not accessing RISTRA at KP.

***Column C: Screening questions; is abdominal pain diffuse, is there right sided abdominal pain, pain for 5 days or less

		Column A*	Column B**	Column C***	Column D	Column E
Label	Site(s)	Potential eligible patients (n)	Screening questions not completed n (%)	Excluded based on screening questions n (%)	ENROLLED n (%)	# months in study (starting with intervention go live)
HP Cluster 1 intervention						
HP Cluster 1, control						
HP Cluster 2, intervention						
HP Cluster 2, control						
KP Cluster 1, intervention						
KP Cluster 1, control						
KP Cluster 2, intervention						
KP Cluster 2, control						
Etc..						

Protection of Human Subjects

a) Risks to Subjects

Human Subjects Involvement and Characteristics

Potentially eligible study subjects will include roughly 25,000 patients 5 to 20.9 years old who present to a participating ED with a chief complaint of acute abdominal pain over a 30-month period. We anticipate approximately 12,600 will have right sided or generalized abdominal pain for <5 days, and no other exclusion criteria, and thus be enrolled.

Study subjects also include roughly 414 clinicians at the two participating hospital systems (17 EDs). To participate in this study, ED clinicians must practice at one of the 17 EDs and be trained in emergency medicine, internal medicine, or family medicine or be a physician extender (nurse practitioner or physician assistant).

Limited clinical data from automated diagnostic, pharmacy, and laboratory databases will be collected for study subjects for specified periods of time to (a) identify eligibility for the study and the analysis based on age, presenting chief complaint, comorbid conditions, and responses to the Appy-CDS system and to (b) assess the impact of study interventions on implementation of Appy-CDS on use of CT and ultrasound and rates of perforated appendicitis, negative appendectomy, and missed appendicitis.

Sources of Materials

All necessary data to determine eligibility, implement and operate the intervention, and assess the impact of the intervention are derived from the EHRs or health plan or medical group administrative databases.

Database Design

A relational database to store patient-level data extracted from administrative and EHR databases at each site will be developed, linking information from different time points during the ED visit and retrieving chief complaints, laboratory data, radiology data, disposition final diagnoses, and responses to any interactive prompts to a specific individual on a specific date using a fixed patient identifier. In addition, diagnoses from previous ED visits will be retrieved.

Data Standardization and Validation Studies

We will establish common study variable definitions drawn from national standards and definitions used in previous studies.[122] We will construct variable definitions and data-extraction procedures for demographics, enrollment characteristics, chief complaints, previous diagnoses, laboratory values (WBC count), use of CT and ultrasound (whether imaging study was conducted), operative and pathology reports, and patient disposition (discharge, admission, operation, or transfer). We will develop conceptual and operational definitions and technical specifications for data elements without established definitions. Data from all sources will be restructured into a common format and data elements combined into uniform files. All person-level information will be linked by a unique identifier so data can be compiled to the person level. Data integrity will be assessed to ensure that observations are valid, reliable, and consistent. Each variable will be tested for completeness and out-of-range values.

Data Security

To protect patient confidentiality, we will create an analytic dataset that includes only encrypted study

identifiers. The analytic dataset will be created from various databases that include the actual patient identifiers. We will use an encryption algorithm to create the encrypted study identifiers. For KPNC, only analytic datasets will be transferred to HPMG. These datasets will include the minimum necessary data for conducting the primary analyses. A table that maps encrypted study identifiers to actual patient identifiers will be stored at the Institute. We will also adhere to all requirements imposed by the governing Institutional Review Board (IRB) and all legal requirements such as HIPAA.

Potential Risks

Risks to ED clinician study subjects are considered minimal and principally involve consideration of the risk of violation of confidentiality of study data. If confidentiality were breached and quality of care were seriously out of range for one or more ED clinicians, leadership of the participating hospital systems could conceivably use this information to release one or more clinicians from employment with the respective participating ED. Therefore, no identifying information on individual ED clinician performance with respect to the clinical domains addressed in this study or any other aspect of care gathered as part of this research project will be made available to hospital or ED leaders who make employment, compensation, or disciplinary decisions. Furthermore, this research project will not alter pre-existing hospital policy of using quality-of-care assessments to evaluate the performance of ED clinicians. Potential risks to study subjects who are patients include the possibility that the intervention may provide CDS advice to ED clinicians that may be inappropriate for a given individual patient and, if applied without further checking the clinical status of a given patient, could lead to erroneous therapy, adverse events, disability, or death. However, the clinical recommendations are primarily related to giving clinicians a risk assessment for appendicitis based on published data and clinical consensus among physician leaders in each participating institution. Therefore, the risk of untoward consequences of such clinical actions is low. Moreover, this potential risk is routinely present in every clinical encounter in the health care system. We have described below the methods used to minimize this risk.

b) Adequacy of Protection Against Risks

Recruitment and Informed Consent

Potential adverse effects on the patients: We have requested and received waivers of written informed consent from the KPNC and HP IRBs, for patients for the following reasons: (a) All open treatment options identified by the intervention are evidence-based and will have been agreed to as the standard of care by key stakeholders at participating hospitals. Furthermore, no care is advocated that would deviate substantially from the standard of care for pediatric patients with abdominal pain. Therefore, our intervention does not represent any additional risk to patients beyond the routine risk that all patients assume whenever they have contact with the medical care system. (b) At intervention training sessions, we will emphasize that it is inappropriate for a ED clinician to follow suggested treatment options without further checking the clinical status of a given patient and that they must use our CDS tools as adjuncts, not replacements for clinical judgment. (c) It would be impractical to consent patients (due to large numbers of patients—up to 10% of all ED visits) and impossible to answer the primary research questions (due to selection effects related to consent) if written informed consent of patients were required. Although unlikely, there is a small chance that implementation of the acute abdominal pain CDS tool will increase the likelihood of a missed case of appendicitis. We will monitor for missed cases of appendicitis and rate of perforated appendicitis, along with other important safety outcomes throughout the study and report missed cases to the Independent Data Monitoring Committee (IDMC).

Potential adverse effects for the ED providers: In participating EDs, we have requested and received

waivers of informed consent from the KPNC and HPC IRBs for the clinicians participating in the CDS trial for the following reasons: (a) All treatment options included in the CDS algorithms will be based on the best available clinical evidence, and all recommendations will be reviewed with site-based clinical leaders. Thus, the care recommendations will conform to current standards of care and ought not to represent any additional risk.

Because clinicians will maintain autonomy regarding the decision to obtain a CT or ultrasound, we do not expect legal consequences related to recommendations given as part of the CDS. Clinicians' names will not be transmitted to the data collection center. Drs. Anupam Kharbanda, Elyse Kharbanda, Dustin Ballard, David Vinson, Michael Zwank and Patrick O'Connor have no role in employing the ED clinicians who may be involved in this study.

Protection against Risk

The following measures will be taken to protect ED clinicians and patients from the risk of breach of confidentiality: A unique study ID code unrelated to the medical record number or other study subject-specific information will be assigned to each patient and provider study subject and used to link data from various sources and needed for analysis. A crosswalk table linking this code number to a provider or patient name or IRB medical record number will be destroyed 12 months after completion of the linked databases needed to test study hypotheses. The written informed consent procedure and documentation for ED clinicians will be reviewed in advance, approved, and monitored on an ongoing basis by the IRB at the respective hospital sites.

The following measures will be taken to minimize the risk that an ED clinician will act wrongly on the basis of information provided through CDS developed for this study: Each project-related communication to providers will include a written explanation indicating that the CDS is a suggestion, not a mandate, and that the action should be taken only if judged clinically appropriate by the treating provider on the basis of the patient's health, previous health care, current treatment, and other factors.

c) Potential Benefits of the Proposed Research to the Subjects and Others

No claim is made in communications with ED provider study subjects that any personal benefit will accrue from participating in this project. Neither clinicians nor other clinical staff will have any defined benefits from participating in this project aside from small, monetary group incentives (raffles) designed to encourage familiarity with the Appy-CDS system. The CDS designed to optimize identification and management of appendicitis in children may familiarize some clinicians with new and potentially useful information that can be used to improve the care they provide. ED clinicians at participating sites will not receive direct monetary compensation for time devoted to the study. All incentives will be approved by HealthPartners and Kaiser Permanente IRBs prior to implementing.

Patient study subjects and their parents or legal guardians will have no defined personal benefit from participating in this project and will receive no compensation. No communication between research team members and study subject patients is planned as part of the study protocol. Although some patients may receive better identification or management of their abdominal pain as a result of this intervention, no claim of clinical benefit to an individual patient can or will be made. There will be no compensation for participation. The minimal risks of study participation are reasonable, despite the lack of direct benefit for study participation.

Importance of the Knowledge to be Gained

If the interventions significantly improve identification of appendicitis and the management of children with acute abdominal pain, we expect to observe a decrease in the use of CT for these patients. This will have the effect of decreasing the exposure to ionizing radiation for the individual patient and thus lowering the associated risk of cancer. If the interventions fail to significantly improve identification of patients with appendicitis, that knowledge will also be important because it will direct the attention of investigators to other, potentially more fruitful, lines of investigation. Thus, regardless of specific findings, the results of this trial will provide important new knowledge that will ultimately contribute to improved care of pediatric patients with acute abdominal pain.

Collaborating Sites

This study will be conducted at emergency departments operated by HealthPartners Medical Group (HPMG) and Kaiser Permanente Northern California (KPNC). There will be subcontracts to KPNC and Children's Hospitals and Clinics of Minnesota. DSMB members will be selected from institutions other than HPMG, Children's Hospitals and Clinics of Minnesota, or KPNC, as described below. No other institutional subcontracts are anticipated.

Special Populations

The study will include pediatric and adolescent patients 5 to 20.9 years old at study entry. Appendicitis is the most common surgical emergency for this age group; thus, it is important to study this disease in this population. Furthermore, this age group has been excluded or underrepresented in previous research studies that have examined the utility of CDS. To ensure that CDS recommendations for identification and management of acute abdominal pain are clinically appropriate, we will exclude pregnant or postpartum adolescents.

9) Data Safety and Monitoring

Oversight of the trial is provided by the Principal Investigator(s) (PI), Drs. Elyse and Anupam Kharbanda at the HealthPartners sites and Drs. Dustin Ballard and David Vinson at Kaiser Permanente of Northern California. In addition, co-investigators Dr. Uli Chettipally, Dr. Patrick O'Connor, Dr. Gabriela Vazquez-Benitez, and Dr. Mike Zwank will assist with oversight throughout the project.

This study will be conducted as a multi-site, cluster randomized trial that targets a vulnerable population, children. As recommended by NICHD, we will compose an Independent Data Monitoring Committee (IDMC) that includes four members with expertise in clinical trials methodology and the topic areas targeted in the proposed research. In addition, our NICHD Project Officer, will be invited to participate in conference calls. The PI(s) will participate in the DSMB meetings in a limited way as recommended in NIH policy.

The IDMC for this study is guided by two general research principles:

1. Ensuring and enhancing the safety of the study; that is to protect the study participant from unacceptable risk; and

2. Assuring the scientific validity of the study; that is, to protect the data and preserve its integrity.

The members of the IDMC will serve as an independent advisory group and are required to provide recommendations about starting, continuing, and stopping the Appy CDS. More specifically, the IDMC will:

- Review the research protocol, and plans for data and safety monitoring;
- Review methodology used to help maintain the confidentiality of the study data and the results of monitoring by reviewing procedures put in place by investigators to ensure confidentiality;
- Monitor study design, procedures and events that will maximize the safety of the study participants and minimize the risks;
- Evaluate the progress of the study, including periodic assessments of data quality and timeliness, rate of cohort entry, participant risk versus benefit, performance of the study site(s), and other factors that may affect study outcome;
- Consider factors external to the study when relevant information becomes available, such as scientific or therapeutic developments that may have an impact on the safety of the participants or the ethics of the study;
- Review serious adverse event documentation and safety reports and make recommendations regarding protection of the safety of the study participants;
- Report to the participating IRBs, if requested, on the safety and progress of the study;
- Evaluate and report to the participating IRBs on any perceived problems with study conduct, enrollment, sample size, and/or data collection;
- Provide to the participating IRBs a recommendation regarding continuation, termination or other modifications of the study based on the cumulative experience including the observed beneficial or adverse effects of the treatment under study;

The IDMC for the *Appy CDS* is composed of the members listed in the table below. In addition, their high level roles and responsibilities are identified below in Table 2.

Table 2. IDMC Members

Name of Member	Role on IDMC	High Level Responsibilities
Bradley Segura, MD, PhD<br>Assistant Professor, Division of Pediatric Surgery<br>University of Minnesota Masonic Children`s Hospital	Chair of IDMC	To facilitate and summarize discussions

Joseph S. Koopmeiners, PhD<br>Assistant Professor, Division of Biostatistics<br>School of Public Health<br>University of Minnesota	Voting member	To review study protocol, provide expert advice regarding statistical issues including: analytic approach, randomization, safety outcomes, power
Matt Zapala<br>Assistant Professor<br>Pediatric Radiology<br>UCSF School of Medicine	Voting member	To review study protocol, provide expert advice regarding radiological outcomes, potential risks and benefits of intervention
David Schnadower, MD MPH<br>Associate Professor of Pediatrics<br>Fellowship Director and Associate Division Director (Research)<br>Division of Pediatric Emergency Medicine<br>Washington University School of Medicine	Voting member	To review study protocol, provide expert advice regarding emergency medicine outcomes, potential risks and benefits of intervention
Gabriela Vazquez-Benitez, PhD<br>Research Investigator<br>HealthPartners Institute<br>Study Statistician	Advisory member	Will provide overview of randomization procedures, cohort eligibility, a priori power estimates; Will provide ongoing data, as needed for review by IDMC

Both voting members and Advisory members listed above for this IDMC may attend closed sessions for this Committee. Other Study Investigators will not attend closed sessions. In addition, *both* Voting members and Advisory members will have access to all data presented to this Committee. The diagram below in Figure 4 illustrates the relationship between the IDMC and other entities.

Organizational Chart

Figure 4. Organizational Diagram for Appy-CDS

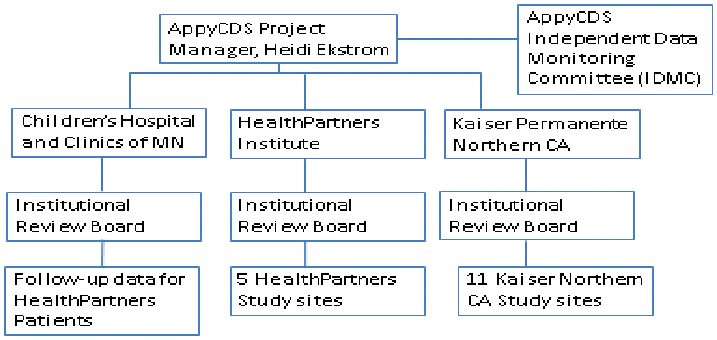

IDMC Communication

Communications for this IDMC will be primarily from the study Project Manager, Heidi Ekstrom. Investigators from Appy-CDS will not communicate directly with IDMC members about the study, except when making presentations or responding to questions at IDMC meetings or during scheduled conference calls.

IDMC Conflict of Interest (COI)

It is extremely important that all members of the IDMC state any real or apparent COIs at the onset of the study. Members of the IDMC shall read the NICHD Clinical Research Guidance Document regarding COI and will provide their signed summary of any COIs to the Study Project Manager, Heidi Ekstrom. Prior to each meeting, all members of the Appy-CDS IDMC will have an opportunity to state whether they have developed any new COIs since the previous meeting. If a new COI is identified, it must be documented in the table above and a new signed summary of the COI should be provided to the Project Manager, to be shared with the Project officer for the study. If a new conflict is reported, the Chair and staff will determine if the conflict limits the ability of the IDMC member to participate in the discussion.

Compensation

All IDMC Voting members will be compensated for their role in supporting the committee. Compensation will be $200 per meeting.

Scheduling, Quorum, and Organization of Meetings

The purpose of the first meeting for the IDMC for EHR-based Decision Support for Pediatric Acute Abdominal Pain in Emergency Care (R01HD079463) or Appy-CDS is to:

- Draft, review, discuss and sign the Charter;
- Designate a Chair for the IDMC
- Provide an overview of AppyCDS activities;
- Review and make recommendations about the study protocol(s); and
- Determine the frequency of interim analyses

In addition to familiarizing the committee with the Appy-CDS study at the first meeting, the IDMC will review plans for following meetings, which are documented in the Table 3 below.

Table 3. IDMC Meeting Content

**Meeting / Review Type**	**Scheduled Time**	**Purpose**	**Required Attendees**
Kickoff Meeting	May 2016, Prior to enrollment of study participants	• Review charter template<br>• Identify data for review and how it should be presented at future meetings<br>• Review protocol including review of statistical analysis plan	All voting and advisory members

Second pre-intervention meeting	Fall 2016, Prior to enrollment of study participants	• Finalize protocol and methods to monitor<br>• Review data from "pre-intervention" period	All voting and advisory members
Third pre-intervention meeting	Spring 2017	• Finalize protocol, clinical recommendations<br>• Finalize monitoring plan<br>• Review data from "pre-intervention" period	All voting and advisory members
Regularly scheduled conference calls	Fall 2017 and then every 6 months	• Review data on enrollment, missed eligible<br>• Review safety outcomes	All voting and advisory members

It is expected that all IDMC members who are identified in the table above will attend every meeting. However, it is recognized that this may not always be possible. Therefore, the IDMC for Appy-CDS has established the following quorum for voting. A quorum of this IDMC is considered to be the IDMC statistician and 2 additional IDMC members are present in-person or by phone. Quorum must be reached in order for an item to be voted on.

Materials and Protocol for IDMC Meetings

The agenda for IDMC meetings and calls will be drafted by the study PIs and project manager. The IDMC Chair will review the finalized meeting minutes prior to distribution to the group.

The agenda and meeting materials will be distributed to the IDMC by the project manager before each meeting or call to allow members adequate time to prepare for the meeting. Meeting materials, once the full intervention has begun, will include the following reports and data:

- Adverse event data
- Other safety data
- Quality and completeness of study data
- Enrollment data

The IDMC will review the above information at each meeting to ensure proper conduct of the study. Details regarding the proposed data to be reviewed and approach to analyses are detailed below.

Monitoring Procedures

A passive surveillance strategy is proposed to monitor adverse events in the study population. Programs will be automated to collect appropriate safety variables and data analyzed by KP and HP programmers/analysts and the study statistician, Dr. Gabriela Vazquez-Benitez. Clinical records for all potential adverse events will be reviewed by one or more investigators at that site. Reports will be presented at biannual meetings and summaries of IDMC recommendations will be submitted to participating IRBs.

Study data are accessible at all times for the PI and co-investigators to review. During the intervention

period, the PI and co-investigators will monitor study conduct (subject accrual, CDS use rates) on a bi-weekly basis. The PIs, co-investigators, and IDMC will safety data in aggregate on a bi-annual basis. The PIs will ensure that all protocol deviations, AEs, and SAEs are reported to the NICHD and IRB according to the applicable regulatory requirements.

This is a minimal risk study that proposes a passive safety surveillance strategy. To evaluate potential adverse events, we will collect and report on automated data for patients with a chief complaint of abdominal pain at intervention and control ED sites. Specific safety outcomes to be evaluated will be reviewed and approved by the IDMC. Proposed safety outcomes are listed in Tables 4-7 below.

Table 4. Appendicitis Specific Outcomes

Condition	Description	How outcome will be identified
Appendiceal perforation	Appendicitis with perforation of the appendix, resulting in purulent material in the peritoneal cavity; can include abscess formation	1. Chart review of surgeon's operative note; Key terms "presence of abscess", "peritonitis", "complex appendicitis", "purulent material" "ruptured appendix", or "likely perforated appendicitis"<br>2. ICD-10 for appendicitis with local or generalized peritonitis (K35.2, K35.3) with hospitalization for intravenous antibiotics who do not undergo appendectomy on initial hospitalization<br>3. We will report appendiceal perforation rates, among those with appendicitis
Negative appendectomy	An appendectomy occurring in the setting of acute abdominal pain with normal pathology, no evidence of inflammation, where an appendectomy is the only surgical procedure performed.	1. Chart review of appendix pathology reports<br>2. Does *not* include incidental appendectomies, if appendectomy is part of more extensive abdominal procedure
Missed appendicitis	A patient who is evaluated in a study ED for acute abdominal pain is discharged home and then returns, within 7 days, with appendicitis	1. Review of EHR and claims data for ICD-10 codes: K35, K35.2, K35.3, K35.8, K35.89, K36, K37 within 12 days of index ED visit, to account for diagnosis date to be hospital discharge date. Followed by manual review to confirm whether appendicitis was within 7 days of index ED visit.

Table 5. Health Utilization Outcomes

Condition	Description	How outcome will be identified
ED return visits within 7 days	Within 7 days of index ED visit, did the patient have another ED visit to the same institution or a different institution	1. Billing records<br>2. EHR<br>3. Chart review
Disposition	Plan following index ED visit	1. Discharge home<br>2. Admitted to inpatient unit
ICU admission	Hospitalization in an intensive care unit (ICU)	1. CPT codes<br>2. Chart review

ED length of stay	Time from initial triage to disposition, for index ED visit.	Time stamps from EHR data
Hospital length of stay	Number of consecutive days in an inpatient setting (includes short stay, observation unit and ICU).	1. Billing records<br>2. Chart review to confirm

Table 6. Missed Care for Other Abdominal Emergencies

Condition	Description	How outcome will be identified
Medical emergencies that require intervention	Small bowel obstruction, tub-ovarian abscess, pelvic inflammatory disease, pyelonephritis	1. Diagnoses within 7 days of index ED visit will be identified from EHR and billing records via ICD-10 codes<br>2. Potential cases will be manually reviewed by site PIs to determine whether there was a delay in definitive care
Surgical emergencies that require intervention	ovarian torsion; testicular torsion, abdominal tumor, intussusception, volvulus, ectopic pregnancy	1. Diagnoses within 7 days of index ED visit will be identified from EHR and billing records via ICD-10 codes<br>2. Potential cases will be manually reviewed by site PIs to determine whether there was a delay in definitive care

Safety outcome rates for intervention and control EDs will be reported to the IDMC every 6 months throughout the 2 year cluster randomized trial. Eligibility and enrollment will also be tracked and reported to the IDMC every 6 months, starting in the pre-intervention period. An example of the enrollment table is shown below (Table 7).

Table 7. Example Enrollment Table

	HP		KP	
	Intervention<br>2 clusters	Control<br>2 clusters	Intervention<br>4 clusters	Control<br>4 clusters
**Pre-Intervention period (n)**	**204**	**75**	**853**	**767**
**Per cluster (n)**	**102**	**38**	**213**	**192**
Goal per cluster (n)	200	200	200	200
Intervention period (n)	114	88	317	240
Projected annual	342	264	1268	960
**Projected over 2-year intervention period**	**684**	**528**	**2536**	**1920**
**Projected per cluster (n)**	**342**	**264**	**634**	**480**
**Goal per cluster**	**400**	**400**	**400**	**400**

Missed eligibles may occur at several stages. First, the Triage BPA may not fire due to changes in workflow and timing of nurse charting of chief complaint. Second, the Triage BPA may not be completed, thus eligibility cannot be confirmed. Third, patients with final diagnosis unavailable, perhaps due to transfer to an outside facility, cannot be captured.

We will compare ED visits for the target age group with abdominal pain as a chief complaint to confirm either cohort entry or intentional exclusion. Those who appear to be eligible but not included in the cohort will be listed as missed eligible. On a quarterly basis we will review missed eligible cases and review for cases of appendicitis in this group.

Meeting Protocol

IDMC meetings and calls for the Appy-CDS will be organized into open, closed, and executive sessions. Definitions for each meeting type are included below. The meeting type will be identified by the project manager when it provides the IDMC Chair with the meeting agenda.

Open sessions

Information will be presented to the IDMC by the study investigators, with time for discussion.

Closed sessions

The IDMC, study statistician, and Study Project Manager will discuss confidential data from the study, including information on efficacy and safety by treatment arm.

The IDMC may decide whether to remain masked to the treatment assignments at each meeting. If the closed session occurs on a conference call, steps will be taken to ensure that only the appropriate participants are on the call and to invite others to re-join the call only at the conclusion of the closed session.

Executive sessions

Only the IDMC members are present to discuss study issues independently. Voting on recommendations will follow Roberts' Rules of Order (Robert's Rules of Order Newly Revised (10th Edition) by Henry M. Robert III, William J. Evans (Editor), Daniel H. Honemann (Editor), Thomas J. Balch (Editor), Sarah Corbin Robert, Henry M. Robert III, General Henry M. Robert).

If the executive session occurs on a conference call, steps will be taken to ensure that only the appropriate participants are on the call and to invite others to re-join the call only at the conclusion of the executive session.

At the conclusion of the closed and executive sessions, all participants will re-convene so that the IDMC Chair can provide a summary of the IDMC's recommendations. This process provides an opportunity for study investigators to ask questions to clarify the recommendations. The meeting is then adjourned.

Reporting Requirements for the AppyCDS IDMC

Proper records will be collected at each IDMC meeting to ensure that there is a physical record of any and all decisions and recommendations. The required documentation for IDMC meetings for the AppyCDS includes the following:

Initial summary
The Study Project Manager is responsible for assuring the accuracy and transmission of a brief summary of the IDMC's discussion and recommendations for the NICHD Project Officer within 48 hours of the meeting or call. The Project Officer will review this summary and approve/disapprove the recommendation(s) or request additional information. The recommendations will then be sent to the Study Project Manager and the clinical investigators.

Formal minutes
The Study Project Manager is responsible for the accuracy the formal IDMC minutes within 30 days of the meeting or call. These minutes are prepared to summarize the key points of the discussion and debate, requests for additional information, response of the investigators to previous recommendations, and the recommendations from the current meeting.

Action plan
If the IDMC's recommendations require significant changes or follow-up, the Study Project Manager and Study Investigators will collaborate to prepare an action plan outlining the steps required to implement the recommendations.

Reports of IDMC Proceedings for IRBs

As Appy-CDS is a multi-center study, this committee may be required to submit reports to IRBs at each of the participating sites. The participating sites for this study are outlined in Table 9 below.

Table 8. Study Site and Contacts

Site of Study	Point of Contact at each Clinical Site for Reporting	Contact Info
HealthPartners Institute	*Heidi Ekstrom, Project Manager*	*Heidi.l.ekstrom@healthpartners.com*
Kaiser Permanente Northern CA	*Adina Rauchwerger, Project Manager*	*Adina.s.rauchwerger@kp.org*

If concerns are identified, the report to the clinical centers will outline those concerns, the IDMC discussion of the concerns, and the basis for any recommendations that the IDMC makes in response to the concerns. The report will be distributed by the Study Project Manager to each clinical center involved in the study. It is the responsibility of each clinical center to forward this information to the local IRB.

10) Project Organization and Management

The organizational chart and project timeline are provided in the budget justification section of this grant. As co-PIs, Drs. Elyse and Anupam Kharbanda will co-lead bi-monthly meetings of the research team at HealthPartners to ensure that all necessary tasks are completed in a timely fashion and strictly according to study protocol. Study teams from KPNC will be invited to join these project meetings via Web conference. Drs. Anupam and Elyse Kharbanda will meet with KPNC investigators in-person at least once per year. Because this project includes a staggered, 2-stage intervention (*Triage CDS* and full Appy- CDS), the development, implementation and reporting and analysis phases overlap. In **Development or Phase 1 (Months 0 to 32),** the team will develop, pilot, and revise the Appy-CDS (triage and full) interventions, including both the EHR-based algorithms to identify and extract necessary data in real time, and the Web service algorithms that process this information and return it to the EHR, where it is retained and displayed. In **Phase 2 (Months 12 to 50)**, the *Triage CDS* will be implemented across the 2 health systems and run for 12 months. Subsequently, following randomization, the full Appy-CDS will be implemented at intervention sites and continue for 24 months. **Phase 3 (Months 27 to 60)** includes import of automated data, completion of chart reviews, data analysis, and reporting of key study results. This process will begin during the course of the 2-year full intervention and allows an additional 6 months following completion of the intervention for availability of claims data for cost outcomes. Throughout the study, there will be ongoing analysis of data as they become available, and preliminary reports on the study's conceptual models, intervention strategies, and preliminary and final results will be presented at meetings and/or reported in peer-reviewed articles.

Strengths and Limitations of the Study

A few limitations to this study should be noted. First, unlike our ongoing work in hypertension and diabetes, the recommendations embedded in Appy-CDS are evidence- based but are not backed by national guidelines. This lack of guidelines should not be perceived as a lack of consensus on best practices for this population. In fact, surgeons and ED providers increasingly agree on the value of risk stratification and the need to limit CT for children with acute abdominal pain.[30,55,57,58] Furthermore, if evidence changes or new appendicitis guidelines are released during the study, we can easily modify the Web-based clinical algorithms. Second, identifying the optimal target population for Appy-CDS using automated data from the EHR in real time is challenging. In order to make comparisons across multiple EDs, it is important to identify a similar population of patients with acute abdominal pain. In our previous multi-center studies, we overcame this obstacle by using on-site research assistants; this approach is neither feasible for the current multisite study nor scalable for widespread dissemination.[20,21] In the current proposal, we will use data provided by the triage nurse along with data retrieved from the EHR to create comparable cohorts across control and intervention sites. In the development phase, we will monitor to ensure that *Triage CDS* is neither overly sensitive nor overly specific and that we are providing alerts for the "right" group of patients.

These potential limitations should be weighed against the strengths of this ambitious, timely project. Namely we are proposing to conduct a large, cluster-randomized trial in 14 general EDs, serving a diverse pediatric population across two regions. Our sophisticated Web-based EHR-linked CDS system will provide a personalized risk assessment and tailored recommendations at the point of care. This novel approach could serve as the basis for future ED interventions. Our track record supports our ability to successfully conduct the proposed project. During a 10-year period, members of our research team have developed an EHR-linked CDS that improved BP and glucose control in type 2 diabetes patients in a randomized trial,[67] and we are currently applying this strategy to improve the recognition of abnormal blood pressure

in adolescents and to optimize management for pediatric patients with a head injury. In the field of appendicitis, we have developed and validated a rule to identify children with acute abdominal pain at low risk for appendicitis. In addition, we have successfully piloted an EHR-linked time-of-ordering alert to reduce CT use in 2 large pediatric EDs.[54] The current proposal is a natural extension of our team's substantial previous work and expertise in CDS[66,123] and appendicitis. Key elements of the intervention have been successfully used in previous projects, enabling us to immediately focus on the substantial enhancements needed to implement and evaluate the Appy-CDS.

Dissemination

Results of this research will be presented at national meetings and in peer-reviewed journals. In addition, the investigators are available for any dissemination activities that NICHD staff deem appropriate. As risks associated with pediatric exposures to ionizing radiation are increasingly publicized, efforts to reduce these exposures will be of widespread interest. Because ED clinicians may hesitate to discharge patients with acute abdominal pain without the reassurance of a negative CT, our safety outcomes are particularly important. If successful, next steps would be broad implementation of this evidence-based, point-of-care Appy-CDS system.

a) Purpose of Document

This document serves as a reference guide for study operations, contacts, and describes the technical details of the study intervention. This document is to be used by the Site Principal Investigators (PI), Research Assistants (RA), and/or nursing staff at each participating medical center to ensure that the study procedures are followed as uniformly as possible. It includes details regarding data collection, flow, data entry, recording and encoding of data.

b) Yearly Medical Center IRB Reviews

It is standard policy in clinical research that all studies involving human subjects be reviewed at least annually by the Institutional Review Board(s) for all participating sites. These reviews are to be done on the anniversary of the initial reviews. Reviews may be conducted more frequently than yearly at the discretion of the various IRBs. It is the Site Investigators' with the help of site project managers or coordinators, and the Institutional Review Boards' responsibility to ensure that yearly reviews occur.

c) Participating Sites and Contacts

Table 10 includes site contact information for each emergency department participating directly in the Appy CDS project. There are 6 sites from HealthPartners Medical Group and 11 sites from Kaiser Permanente Northern California.

Table 9. Site Emergency Department Information

Site #	Site Name	Contact	Site PI
**01**	Regions	H. Ekstrom/S. Wewerka	**A/E. Kharbanda**
**02**	Lakeview	H. Ekstrom/S. Wewerka	**A/E. Kharbanda**
**03**	Westfield	H. Ekstrom/S. Wewerka	**A/E. Kharbanda**
**04**	Hudson	H. Ekstrom/S. Wewerka	**A/E. Kharbanda**
**05**	Amery	H. Ekstrom/S. Wewerka	**A/E. Kharbanda**
**06**	Methodist	H. Ekstrom/S. Wewerka	**A/E. Kharbanda**
**07**	Sacramento	A. Rauchwerger	**D. Ballard/D. Vinson**

**08**	Roseville	A. Rauchwerger	**D. Ballard/D. Vinson**
**09**	South San Francisco	A. Rauchwerger	**D. Ballard/D. Vinson**
**10**	San Rafael	A. Rauchwerger	**D. Ballard/D. Vinson**
**11**	Santa Clara	A. Rauchwerger	**D. Ballard/D. Vinson**
**12**	South Sacramento	A. Rauchwerger	**D. Ballard/D. Vinson**
**13**	San Leandro	A. Rauchwerger	**D. Ballard/D. Vinson**
**14**	Fremont	A. Rauchwerger	**D. Ballard/D. Vinson**
**15**	Oakland	A. Rauchwerger	**D. Ballard/D. Vinson**
**16**	Richmond	A. Rauchwerger	**D. Ballard/D. Vinson**
**17**	**San Francisco**	**A. Rauchwerger**	**D. Ballard/D. Vinson**

Children's Hospital and Clinics of Minnesota (Children's) will not participate in patient recruitment but will contribute data to the study as it is the primary site of referral for pediatric patients from HealthPartners requiring surgical evaluation. Records from Children's will be reviewed to determine final diagnosis for patients transferred from HealthPartners. The Children's contact is Brianna McMichael. Children's PI is Dr. Anupam Kharbanda.

d) Directory of Study Personnel

The comprehensive list of study personnel is listed in Table 10 below.

Table 10. Study Personnel

Name	Role on Project	Contact
HealthPartners		
Elyse Kharbanda	Study Co- PI	Elyse.o.kharbanda@healthpartners.com
Patrick O'Connor	Co-Investigator	Patrick.j.oconnor@healthpartners.com
Jingyi Zhu	Programmer	Jingyi.X.Zhu@HealthPartners.Com
Gaby Vazquez-Benitez	Co-Investigator, Statistician	Gabriela.x.vazquezbenitez@healthpartners.com
Steve Dehmer	Co-Investigator, Economist	Steven.p.dehmer@healthpartners.com
Mike Zwank	Co-Investigator	Michael.d.zwank@healthpartners.com
Heidi Ekstrom	Project manager	Heidi.l.ekstrom@healthpartners.com
Sandi Wewerka	Manager in charge of training	Sandi.s.wewerka@healthpartners.com
Rashmi Sharma	Epic programmer	Rashmi.x.sharma@healthpartners.com
Gopi Kunisetty	Application developer	Gopikrishna.x.kunisetty@healthpartners.com
Children's		
Anupam Kharbanda	Study Co-PI	Anupam.lharbanda@childrensmn.org
Brianna McMichael	Project manager	Brianna.McMichael@childrensmn.org
Kaiser Permanente NC		
Dustin Ballard	Site PI	Dballard30@gmail
David Vinson	Site Co-PI	drvinson@ucdavis.edu
Uli Chettipally	Co-Investigator	Uli.chettipally@gmail.com
Mamata Kene	Co-Investigator	mvkene@gmail.com
Adina Rauchwerger	Project Manager	Adina.s.rauchwerger@kp.org
Laurie Simon	Project Coordinator	Laura.E.Simon@kp.org
Margaret Warton	Programmer	Margaret.M.Warton@kp.org

e) Advisory Board Members

The comprehensive lists of advisory board members are listed in Table 11 and Table 12 below.

Table 11. Advisory Board Members for Kaiser Permanente Northern California

Name	Facility	Role
Arvind Sonik	Sac - Ped Rad	Pediatric Radiologist
Donald J Haugen	Ros - Peds HBS	Pediatric Hospitalist, Chief of Inpatient Pediatrics for the North Valley
Douglas N Miniati	Ros - Ped Surg	Pediatric Surgeon, Surgical Institutional Responsible Investigator
Elliott R Brill	SCL - Surgery	General Surgeon
Eric M Padua	Oak - Emergency	ED Physician
Jeffrey J Du Bois	Ros - Ped Surg	Pediatric General Surgeon, Chief of Children's Surgical Services
Karen Murrell	SSC - Emergency	ED Physician
Kerry Sullivan	SCL - Pediatric Surgery	Pediatric Surgeon
Robert P Norris	Sac - Emergency	ED Physician
Stevan J Cavalier	Wcr - Ped/NCAL Dir	Pediatrician, Regional Direction of Inpatient Pediatrics (Ncal)
Steven M LeVine	Oak - Emergency/HealthConnect	ED Physician
Teresa Orrante	SSF - ED Charge RN III	ED Charge Nurse
Victoria A Clague	SRF - Radiology	Radiologist
Todd K Osinski	RWC - Interventional Radiology	Radiologist
Sundeep M Nayak	SLN - Nuclear Medicine	Radiologist
Shaun Loh	SRF - Radiology	Radiologist
Tariq Chaudry	SCH - Pediatrics	Regional Dir of Inpatient Pediatrics

Table 12. Advisory Board Members for HealthPartners

**Name**	**Facility**	Role
David Schmeling	Children's of MN	Chief Pediatric Surgery
Bill Mize	Children's of MN	Medical Director Radiology
David Dries	HP Regions Hospital	Surgeon
Azhar Ali	HP Regions Hospital	Radiologist
Rachel Zacher	HP Regions Hospital	Supervisor ER RN
Brad Gordon	HP Regions Hospital	Adult ED/IT
Kurt Isenberger	HP Regions Hospital	ED Medical Director
Martin Richards	HP Regions Hospital	Leader, Lakeview, Hudson, Westfield EDs
David Julson	HP Regions Hospital	IS&T Analyst

11) Investigator and Research Staff Responsibilities

a) IRB related issues

1. Read and understand protocol and study procedures (PI/PM/RA).
2. Verify that all those required by site IRB to complete Human Subjects training and have certification on file (PI/PM/RA).
3. Submit Study for site IRB approval (PI/PM).
4. Maintain file of signature and delegated responsibilities forms (PM).
5. Maintain Essential Document Binder and Manual of Operations (MOO) (PI/PM).
6. Maintain hard copies of all patient logs (PI/PM/RA).
7. Comply with site monitoring and data oversight (PI/PM/RA).

b) Study related issues

1. Educate nurses, ED physicians, fellows and residents on purpose of study and methods.
    a. Present study at ED specific conferences, resident lectures, nursing meetings, and other staff meetings as appropriate.
2. Maintain a log of enrolled, excluded and missed-eligible patients

c) General Responsibilities

1. The scientific community in general and investigators in particular, are expected to make every effort to prevent scientific misconduct.

2. Primary responsibility for ensuring the authenticity of reported data rests with the principal Investigator(s). In addition, all investigators identified as authors of a report also assume responsibility for its authenticity. An investigator must not knowingly represent as empirical observations data which are newly synthesized, or arbitrarily altered.

3. The appropriate response to a complaint of fraudulent presentation of data is to review the original experimental records. All investigators have the responsibility to maintain a record of all experimental protocols and data sufficient to allow subsequent verification. Written, detailed, and explicit procedures for data gathering, storage, retrieval and analysis must be available at all sites.

4. Principal Investigators have the responsibility to ensure proper supervision of the research not performed directly by them. Trainees must be supervised by an experienced physician, and they should be encouraged to present their studies at review sessions or seminars. Publications must give credit to all investigators involved in the research and all publications must be approved by all coauthors.

5. All sites must ensure that sufficient management controls are in effect to preclude the occurrences of all unethical scientific practices in research. Examples of violations of ethical standards include:

a. Deliberate fabrication or falsification in the conduct or reporting of research data;

b. Plagiarism in scientific publications or in applications for research support;

c. Practices that seriously deviate from those commonly accepted within the scientific community for proposing, reporting or conducting research;

d. Misappropriation of research funds.

e. Violation of laws established for the protection of human and animal subjects.

12) Literature Cited

1. Pitts SR. *National Hospital Ambulatory Medical Care Survey: 2006 Emergency Department Summary.* 2006.
2. Scholer SJ, Pituch K, Orr DP, Dittus RS. Clinical outcomes of children with acute abdominal pain. *Pediatrics.* 1996;98(4 Pt 1):680-685.
3. Reynolds SL, Jaffe DM. Diagnosing abdominal pain in a pediatric emergency department. *Pediatric emergency care.* 1992;8(3):126-128.
4. Fahimi J, Herring A, Harries A, Gonzales R, Alter H. Computed tomography use among children presenting to emergency departments with abdominal pain. *Pediatrics.* 2012;130(5):e1069-1075.
5. Bachur RG, Hennelly K, Callahan MJ, Monuteaux MC. Advanced radiologic imaging for pediatric appendicitis, 2005-2009: trends and outcomes. *The Journal of pediatrics.* 2012;160(6):1034-1038.
6. Saito JM, Yan Y, Evashwick TW, Warner BW, Tarr PI. Use and accuracy of diagnostic imaging by hospital type in pediatric appendicitis. *Pediatrics.* 2013;131(1):e37-44.
7. Hryhorczuk AL, Mannix RC, Taylor GA. Pediatric abdominal pain: use of imaging in the emergency department in the United States from 1999 to 2007. *Radiology.* 2012;263(3):778-785.
8. Gausche-Hill M, Schmitz C, Lewis RJ. Pediatric preparedness of US emergency departments: a 2003 survey. *Pediatrics.* 2007;120(6):1229-1237.
9. Larson DB, Johnson LW, Schnell BM, Goske MJ, Salisbury SR, Forman HP. Rising Use of CT in Child Visits to the Emergency Department in the United States, 1995-2008. *Radiology.* 2011.
10. Safran DB, Pilati D, Folz E, Oller D. Is appendiceal CT scan overused for evaluating patients with right lower quadrant pain? *Am J Emerg Med.* 2001;19(3):199-203.
11. Lee SL, Walsh AJ, Ho HS. Computed tomography and ultrasonography do not improve and may delay the diagnosis and treatment of acute appendicitis. *Arch Surg.* 2001;136(5):556-562.
12. Pearce MS, Salotti JA, Little MP, et al. Radiation exposure from CT scans in childhood and subsequent risk of leukaemia and brain tumours: a retrospective cohort study. *Lancet.* 2012;380(9840):499-505.
13. Brenner D, Elliston C, Hall E, Berdon W. Estimated risks of radiation-induced fatal cancer from pediatric CT. *AJR Am J Roentgenol.* 2001;176(2):289-296.
14. Miglioretti DL, Johnson E, Williams A, et al. The use of computed tomography in pediatrics and the associated radiation exposure and estimated cancer risk. *JAMA Pediatr.* 2013;167(8):700-707.
15. Flum DR, Morris A, Koepsell T, Dellinger EP. Has misdiagnosis of appendicitis decreased over time? A population-based analysis. *JAMA.* 2001;286(14):1748-1753.
16. Partrick DA, Janik JE, Janik JS, Bensard DD, Karrer FM. Increased CT scan utilization does not improve the diagnostic accuracy of appendicitis in children. *J Pediatr Surg.* 2003;38(5):659-662.
17. Flum DR, McClure TD, Morris A, Koepsell T. Misdiagnosis of appendicitis and the use of diagnostic imaging. *J Am Coll Surg.* 2005;201(6):933-939.
18. Martin AE, Vollman D, Adler B, Caniano DA. CT scans may not reduce the negative appendectomy rate in children. *J Pediatr Surg.* 2004;39(6):886-890; discussion 886-890.
19. Newman K, Ponsky T, Kittle K, et al. Appendicitis 2000: variability in practice, outcomes, and resource utilization at thirty pediatric hospitals. *J Pediatr Surg.* 2003;38(3):372-379; discussion 372-379.
20. Kharbanda AB, Taylor GA, Fishman SJ, Bachur RG. A clinical decision rule to identify children at low risk for appendicitis. *Pediatrics.* 2005;116(3):709-716.
21. Kharbanda AB, Dudley NC, Bajaj L, et al. Validation and refinement of a prediction rule to identify children at low risk for acute appendicitis. *Archives of pediatrics & adolescent medicine.* 2012;166(8):738-744.

22. Samuel M. Pediatric appendicitis score. *J Pediatr Surg.* 2002;37(6):877-881.
23. Schneider C, Kharbanda A, Bachur R. Evaluating appendicitis scoring systems using a prospective pediatric cohort. *Ann Emerg Med.* 2007;49(6):778-784, 784 e771.
24. Kulik DM, Uleryk EM, Maguire JL. Does this child have appendicitis? A systematic review of clinical prediction rules for children with acute abdominal pain. *Journal of clinical epidemiology.* 2013;66(1):95-104.
25. Kharbanda AB, Madhok M, Krause E, et al. Implementation of Electronic Clinical Decision Support for Pediatric Appendicitis. *Pediatrics.* 2016;137(5).
26. Rothrock SG, Pagane J. Acute appendicitis in children: emergency department diagnosis and management. *Ann Emerg Med.* 2000;36(1):39-51.
27. Addiss DG, Shaffer N, Fowler BS, Tauxe RV. The epidemiology of appendicitis and appendectomy in the United States. *Am J Epidemiol.* 1990;132(5):910-925.
28. Kaechele V, Wabitsch M, Thiere D, et al. Prevalence of gallbladder stone disease in obese children and adolescents: influence of the degree of obesity, sex, and pubertal development. *Journal of pediatric gastroenterology and nutrition.* 2006;42(1):66-70.
29. Guthrie BD, Adler MD, Powell EC. Incidence and trends of pediatric ovarian torsion hospitalizations in the United States, 2000-2006. *Pediatrics.* 2010;125(3):532-538.
30. Bundy DG, Byerley JS, Liles EA, Perrin EM, Katznelson J, Rice HE. Does this child have appendicitis? *JAMA.* 2007;298(4):438-451.
31. Rothrock SG, Skeoch G, Rush JJ, Johnson NE. Clinical features of misdiagnosed appendicitis in children. *Ann Emerg Med.* 1991;20(1):45-50.
32. Nance ML, Adamson WT, Hedrick HL. Appendicitis in the young child: a continuing diagnostic challenge. *Pediatric emergency care.* 2000;16(3):160-162.
33. Jablonski KA, Guagliardo MF. Pediatric appendicitis rupture rate: a national indicator of disparities in healthcare access. *Population health metrics.* 2005;3(1):4.
34. Muehlstedt SG, Pham TQ, Schmeling DJ. The management of pediatric appendicitis: a survey of North American Pediatric Surgeons. *J Pediatr Surg.* 2004;39(6):875-879; discussion 875-879.
35. Bachur RG, Hennelly K, Callahan MJ, Chen C, Monuteaux MC. Diagnostic imaging and negative appendectomy rates in children: effects of age and gender. *Pediatrics.* 2012;129(5):877-884.
36. Rao PM, Rhea JT, Novelline RA, Mostafavi AA, Lawrason JN, McCabe CJ. Helical CT combined with contrast material administered only through the colon for imaging of suspected appendicitis. *AJR Am J Roentgenol.* 1997;169(5):1275-1280.
37. Garcia Pena BM, Mandl KD, Kraus SJ, et al. Ultrasonography and limited computed tomography in the diagnosis and management of appendicitis in children. *JAMA.* 1999;282(11):1041-1046.
38. Kocher KE, Meurer WJ, Fazel R, Scott PA, Krumholz HM, Nallamothu BK. National trends in use of computed tomography in the emergency department. *Ann Emerg Med.* 2011;58(5):452-462 e453.
39. Smith-Bindman R, Miglioretti DL, Johnson E, et al. Use of diagnostic imaging studies and associated radiation exposure for patients enrolled in large integrated health care systems, 1996-2010. *JAMA.* 2012;307(22):2400-2409.
40. Krajewski S, Brown J, Phang PT, Raval M, Brown CJ. Impact of computed tomography of the abdomen on clinical outcomes in patients with acute right lower quadrant pain: a meta-analysis. *Can J Surg.* 2011;54(1):43-53.
41. Karakas SP, Guelfguat M, Leonidas JC, Springer S, Singh SP. Acute appendicitis in children: comparison of clinical diagnosis with ultrasound and CT imaging. *Pediatr Radiol.* 2000;30(2):94-98.
42. Jones PF. Suspected acute appendicitis: trends in management over 30 years. *Br J Surg.* 2001;88(12):1570-1577.

43. Smith-Bindman R, Lipson J, Marcus R, et al. Radiation dose associated with common computed tomography examinations and the associated lifetime attributable risk of cancer. *Arch Intern Med.* 2009;169(22):2078-2086.
44. Mathews JD, Forsythe AV, Brady Z, et al. Cancer risk in 680,000 people exposed to computed tomography scans in childhood or adolescence: data linkage study of 11 million Australians. *BMJ.* 2013;346:f2360.
45. Brody AS, Frush DP, Huda W, Brent RL. Radiation risk to children from computed tomography. *Pediatrics.* 2007;120(3):677-682.
46. Pritchett CV, Levinsky NC, Ha YP, Dembe AE, Steinberg SM. Management of acute appendicitis: the impact of CT scanning on the bottom line. *J Am Coll Surg.* 2010;210(5):699-705, 705-697.
47. Lin KH, Leung WS, Wang CP, Chen WK. Cost analysis of management in acute appendicitis with CT scanning under a hospital global budgeting scheme. *Emerg Med J.* 2008;25(3):149-152.
48. Kieran JA, Curet MJ, Schermer CR. Institutional variations in the management of patients with acute appendicitis. *J Gastrointest Surg.* 2003;7(4):523-528.
49. Doria AS, Moineddin R, Kellenberger CJ, et al. US or CT for Diagnosis of Appendicitis in Children and Adults? A Meta-Analysis. *Radiology.* 2006;241(1):83-94.
50. Athey J, Dean JM, Ball J, Wiebe R, Melese-d'Hospital I. Ability of hospitals to care for pediatric emergency patients. *Pediatric emergency care.* 2001;17(3):170-174.
51. Larson DB, Johnson LW, Schnell BM, Salisbury SR, Forman HP. National trends in CT use in the emergency department: 1995-2007. *Radiology.* 2011;258(1):164-173.
52. Cuschieri J, Florence M, Flum DR, et al. Negative appendectomy and imaging accuracy in the Washington State Surgical Care and Outcomes Assessment Program. *Ann Surg.* 2008;248(4):557-563.
53. Paterson A, Frush DP, Donnelly LF. Helical CT of the body: are settings adjusted for pediatric patients? *AJR Am J Roentgenol.* 2001;176(2):297-301.
54. Krause E, Madhok M, A K. Implementation of an Appendicitis Clinical Decision Support Tool within an Electronic Health Record System Paper. Paper presented at: SPR/PAS meeting2013; Baltimore, MD.
55. Santillanes G, Simms S, Gausche-Hill M, et al. Prospective evaluation of a clinical practice guideline for diagnosis of appendicitis in children. *Academic emergency medicine : official journal of the Society for Academic Emergency Medicine.* 2012;19(8):886-893.
56. Poortman P, Oostvogel HJ, Bosma E, et al. Improving diagnosis of acute appendicitis: results of a diagnostic pathway with standard use of ultrasonography followed by selective use of CT. *J Am Coll Surg.* 2009;208(3):434-441.
57. Ramarajan N, Krishnamoorthi R, Barth R, et al. An interdisciplinary initiative to reduce radiation exposure: evaluation of appendicitis in a pediatric emergency department with clinical assessment supported by a staged ultrasound and computed tomography pathway. *Academic emergency medicine : official journal of the Society for Academic Emergency Medicine.* 2009;16(11):1258-1265.
58. Saucier A, Huang EY, Emeremni CA, Pershad J. Prospective evaluation of a clinical pathway for suspected appendicitis. *Pediatrics.* 2014;133(1):e88-95.
59. Garcia Pena BM, Cook EF, Mandl KD. Selective imaging strategies for the diagnosis of appendicitis in children. *Pediatrics.* 2004;113(1 Pt 1):24-28.
60. Kharbanda AB, Stevenson MD, Macias CG, et al. Interrater reliability of clinical findings in children with possible appendicitis. *Pediatrics.* 2012;129(4):695-700.
61. Chaudhry B, Wang J, Wu S, et al. Systematic review: impact of health information technology on

quality, efficiency, and costs of medical care. *Ann Intern Med.* 2006;144(10):742-752.
62. Eslami S, Abu-Hanna A, de Keizer NF. Evaluation of outpatient computerized physician medication order entry systems: a systematic review. *J Am Med Inform Assoc.* 2007;14(4):400-406.
63. Garg AX, Adhikari NK, McDonald H, et al. Effects of computerized clinical decision support systems on practitioner performance and patient outcomes: a systematic review. *JAMA.* 2005;293(10):1223-1238.
64. Hunt DL, Haynes RB, Hanna SE, Smith K. Effects of computer-based clinical decision support systems on physician performance and patient outcomes: a systematic review. *JAMA.* 1998;280(15):1339-1346.
65. Johnston ME, Langton KB, Haynes RB, Mathieu A. Effects of computer-based clinical decision support systems on clinician performance and patient outcome. A critical appraisal of research. *Ann Intern Med.* 1994;120(2):135-142.
66. O'Connor PJ, Desai JR, Butler JC, Kharbanda EO, Sperl-Hillen JM. Current status and future prospects for electronic point-of-care clinical decision support in diabetes care. *Curr Diab Rep.* 2013;13(2):172-176.
67. O'Connor PJ, Sperl-Hillen JM, Rush WA, et al. Impact of electronic health record clinical decision support on diabetes care: a randomized trial. *Ann Fam Med.* 2011;9(1):12-21.
68. O'Connor PJ, Desai J, Solberg LI, et al. Randomized trial of quality improvement intervention to improve diabetes care in primary care settings. *Diabetes Care.* 2005;28(8):1890-1897.
69. Ballard DW, Rauchwerger AS, Reed ME, et al. Emergency physicians' knowledge and attitudes of clinical decision support in the electronic health record: a survey-based study. *Acad Emerg Med.* 2013;20(4):352-360.
70. Georgiou A, Prgomet M, Paoloni R, et al. The effect of computerized provider order entry systems on clinical care and work processes in emergency departments: a systematic review of the quantitative literature. *Ann Emerg Med.* 2013;61(6):644-653 e616.
71. Sheehan B, Nigrovic LE, Dayan PS, et al. Informing the design of clinical decision support services for evaluation of children with minor blunt head trauma in the emergency department: a sociotechnical analysis. *J Biomed Inform.* 2013;46(5):905-913.
72. Reilly BM, Evans AT. Translating clinical research into clinical practice: impact of using prediction rules to make decisions. *Ann Intern Med.* 2006;144(3):201-209.
73. Georgiou A, Prgomet M, Markewycz A, Adams E, Westbrook JI. The impact of computerized provider order entry systems on medical-imaging services: a systematic review. *J Am Med Inform Assoc.* 2011;18(3):335-340.
74. Roshanov PS, Fernandes N, Wilczynski JM, et al. Features of effective computerised clinical decision support systems: meta-regression of 162 randomised trials. *BMJ.* 2013;346:f657.
75. Delpierre C, Cuzin L, Fillaux J, Alvarez M, Massip P, Lang T. A systematic review of computer-based patient record systems and quality of care: more randomized clinical trials or a broader approach? *Int J Qual Health Care.* 2004;16(5):407-416.
76. Ash JS, Sittig DF, Dykstra R, et al. Identifying best practices for clinical decision support and knowledge management in the field. *Stud Health Technol Inform.* 2010;160(Pt 2):806-810.
77. Ash JS, Sittig DF, Guappone KP, et al. Recommended practices for computerized clinical decision support and knowledge management in community settings: a qualitative study. *BMC Med Inform Decis Mak.* 2012;12:6.
78. Kuppermann N, Holmes JF, Dayan PS, et al. Identification of children at very low risk of clinically-important brain injuries after head trauma: a prospective cohort study. *Lancet.* 2009;374(9696):1160-1170.

79. Nigrovic LE, Kuppermann N, Macias CG, et al. Clinical prediction rule for identifying children with cerebrospinal fluid pleocytosis at very low risk of bacterial meningitis. *JAMA.* 2007;297(1):52-60.
80. Ebell M. AHRQ White Paper: Use of clinical decision rules for point-of-care decision support. *Med Decis Making.* 2010;30(6):712-721.
81. Mittal MK, Dayan PS, Macias CG, et al. Performance of ultrasound in the diagnosis of appendicitis in children in a multicenter cohort. *Acad Emerg Med.* 2013;20(7):697-702.
82. Schuh S, Man C, Cheng A, et al. Predictors of non-diagnostic ultrasound scanning in children with suspected appendicitis. *The Journal of pediatrics.* 2011;158(1):112-118.
83. Le J, Kurian J, Cohen HW, Weinberg G, Scheinfeld MH. Do clinical outcomes suffer during transition to an ultrasound-first paradigm for the evaluation of acute appendicitis in children? *AJR Am J Roentgenol.* 2013;201(6):1348-1352.
84. Bhatt M, Joseph L, Ducharme FM, Dougherty G, McGillivray D. Prospective validation of the pediatric appendicitis score in a Canadian pediatric emergency department. *Academic emergency medicine : official journal of the Society for Academic Emergency Medicine.* 2009;16(7):591-596.
85. Murray DM, Varnell SP, Blitstein JL. Design and analysis of group-randomized trials: a review of recent methodological developments. *Am J Public Health.* 2004;94(3):423-432.
86. Campbell MJ, Donner A, Klar N. Developments in cluster randomized trials and Statistics in Medicine. *Stat Med.* 2007;26(1):2-19.
87. Ruger JP, Lewis LM, Richter CJ. Patterns and factors associated with intensive use of ED services: implications for allocating resources. *Am J Emerg Med.* 2012;30(9):1884-1894.
88. Gaucher N, Bailey B, Gravel J. Who are the children leaving the emergency department without being seen by a physician? *Academic emergency medicine : official journal of the Society for Academic Emergency Medicine.* 2011;18(2):152-157.
89. Bekmezian A, Chung PJ, Cabana MD, Maselli JH, Hilton JF, Hersh AL. Factors associated with prolonged emergency department length of stay for admitted children. *Pediatric emergency care.* 2011;27(2):110-115.
90. Dalinger EL. A reference model for designing decision support systems in novel work domains: Systems, Man and Cybernetics (SMC). Paper presented at: 2011 IEEE International Conference on 9-12 Oct. 20112011.
91. Ahmed A, Chandra S, Herasevich V, Gajic O, Pickering BW. The effect of two different electronic health record user interfaces on intensive care provider task load, errors of cognition, and performance. *Crit Care Med.* 2011;39(7):1626-1634.
92. Courtney JF. Decision making and knowledge management in inquiring organizations. *Decision Support Systems.* 2001;31:17-38.
93. Razzouk E CT, Almoosa K, Patel V. Approaching the limits of knowledge: the influence of priming on error detection in simulated clinical rounds. AMIA Annu Symp Proc; 2011.
94. Cabana MD, Rand CS, Powe NR, et al. Why don't physicians follow clinical practice guidelines? A framework for improvement. *JAMA.* 1999;282(15):1458-1465.
95. Kawamoto K, Houlihan CA, Balas EA, Lobach DF. Improving clinical practice using clinical decision support systems: a systematic review of trials to identify features critical to success. *BMJ.* 2005;330(7494):765.
96. Becker T, Kharbanda A, Bachur R. Atypical clinical features of pediatric appendicitis. *Acad Emerg Med.* 2007;14(2):124-129.
97. Altman DG, Vergouwe Y, Royston P, Moons KG. Prognosis and prognostic research: validating a prognostic model. *BMJ.* 2009;338:b605.
98. Royston P, Moons KG, Altman DG, Vergouwe Y. Prognosis and prognostic research: Developing a

prognostic model. *BMJ.* 2009;338:b604.
99. Kharbanda A, Vazquez Benitez G, Ballard DW, et al. Development and Validation of a Novel Pediatric Appendicitis Risk Calculator (pARC). 2018 (in press).
100. Zeger SL, Liang KY. Longitudinal data analysis for discrete and continuous outcomes. *Biometrics.* 1986;42(1):121-130.
101. Kraemer HC. Discovering, comparing, and combining moderators of treatment on outcome after randomized clinical trials: a parametric approach. *Stat Med.* 2013;32(11):1964-1973.
102. Manning WG. The logged dependent variable, heteroscedasticity, and the retransformation problem. *J Health Econ.* 1998;17(3):283-295.
103. Manning WG, Basu A, Mullahy J. Generalized modeling approaches to risk adjustment of skewed outcomes data. *J Health Econ.* 2005;24(3):465-488.
104. Myers AL, Williams RF, Giles K, et al. Hospital cost analysis of a prospective, randomized trial of early vs interval appendectomy for perforated appendicitis in children. *J Am Coll Surg.* 2012;214(4):427-434; discussion 434-425.
105. National Quality Forum. Total Cost of Care Population-based PMPM Index (Resource Use Measure). http://www.qualityforum.org/QPS/1604. Accessed 2016.
106. Flottemesch TJ, Fontaine P, Asche SE, Solberg LI. Relationship of clinic medical home scores to health care costs. *J Ambul Care Manage.* 2011;34(1):78-89.
107. Flottemesch TJ, Anderson LH, Solberg LI, Fontaine P, Asche SE. Patient-centered medical home cost reductions limited to complex patients. *Am J Manag Care.* 2012;18(11):677-686.
108. Anderson LH, Flottemesch TJ, Fontaine P, Solberg LI, Asche SE. Patient medical group continuity and healthcare utilization. *Am J Manag Care.* 2012;18(8):450-457.
109. Fontaine P, Flottemesch TJ, Solberg LI, Asche SE. Is consistent primary care within a patient-centered medical home related to utilization patterns and costs? *J Ambul Care Manage.* 2011;34(1):10-19.
110. Flottemesch TJ, Gordon BD, Jones SS. Advanced statistics: developing a formal model of emergency department census and defining operational efficiency. *Academic emergency medicine : official journal of the Society for Academic Emergency Medicine.* 2007;14(9):799-809.
111. Bellow AA, Jr., Flottemesch TJ, Gillespie GL. Application of the emergency department census model. *Adv Emerg Nurs J.* 2012;34(1):55-64.
112. Webster J, Kauffman TL, Feigelson HS, et al. KRAS testing and epidermal growth factor receptor inhibitor treatment for colorectal cancer in community settings. *Cancer Epidemiol Biomarkers Prev.* 2013;22(1):91-101.
113. Flottemesch TJ, Scholle SH, O'Connor PJ, Solberg LI, Asche S, Pawlson LG. Are characteristics of the medical home associated with diabetes care costs? *Med Care.* 2012;50(8):676-684.
114. Maciosek MV, Coffield AB, Flottemesch TJ, Edwards NM, Solberg LI. Greater use of preventive services in U.S. health care could save lives at little or no cost. *Health Aff (Millwood).* 2010;29(9):1656-1660.
115. Maciosek MV, Coffield AB, Edwards NM, Flottemesch TJ, Goodman MJ, Solberg LI. Priorities among effective clinical preventive services: results of a systematic review and analysis. *Am J Prev Med.* 2006;31(1):52-61.
116. Solberg LI, Flottemesch TJ, Foldes SS, Molitor BA, Walker PF, Crain AL. Tobacco-use prevalence in special populations taking advantage of electronic medical records. *Am J Prev Med.* 2008;35(6 Suppl):S501-507.
117. Maciosek MV, Edwards NM, Coffield AB, et al. Priorities among effective clinical preventive services: methods. *Am J Prev Med.* 2006;31(1):90-96.

118. Guittet L, Ravaud P, Giraudeau B. Planning a cluster randomized trial with unequal cluster sizes: practical issues involving continuous outcomes. *BMC Med Res Methodol.* 2006;6:17.
119. Marshall A, Altman DG, Royston P, Holder RL. Comparison of techniques for handling missing covariate data within prognostic modelling studies: a simulation study. *BMC Med Res Methodol.* 2010;10:7.
120. HealthPartners. HealthPartners White Paper: Total Cost of Care (TCOC) and Total Resource Use. 2014; https://www.healthpartners.com/ucm/groups/public/@hp/@public/documents/documents/dev_057649.pdf.
121. Maciosek MV, Coffield AB, Edwards NM, Flottemesch TJ, Solberg LI. Prioritizing clinical preventive services: a review and framework with implications for community preventive services. *Annu Rev Public Health.* 2009;30:341-355.
122. NCQA. HEDIS & Quality Measurement 2013. http://www.ncqa.org/HEDISQualityMeasurement/HEDISMeasures/HEDIS2013.aspx. Accessed 2016.
123. O'Connor P. Opportunities to Increase the Effectiveness of EHR-Based Diabetes Clinical Decision Support. *Appl Clin Inform.* 2011;2(3):350-354.

Appendix A: Intervention Design and Workflow at HP

The Epic triage alert is a best practice advisory (BPA) built in the HP EHR that uses automated data to identify potentially eligible study participants. Data variables include age, chief complaint, pregnancy status, and trauma status. If the patient meets the initial study criteria: chief complaint of abdominal pain, age 5-20, are not pregnant and have no history of trauma, the BPA will display in the ED triage navigator. The advisory prompts the user to click a link which will bring them to a flowsheet section form where they are to complete up to 3 questions that further filter eligibility.

a) Capturing eligibility: Epic Triage BPA

The below figures illustrate the technical architecture of the Triage (Phase 1) Appy-CDS system at HealthPartners.

Triage architecture.

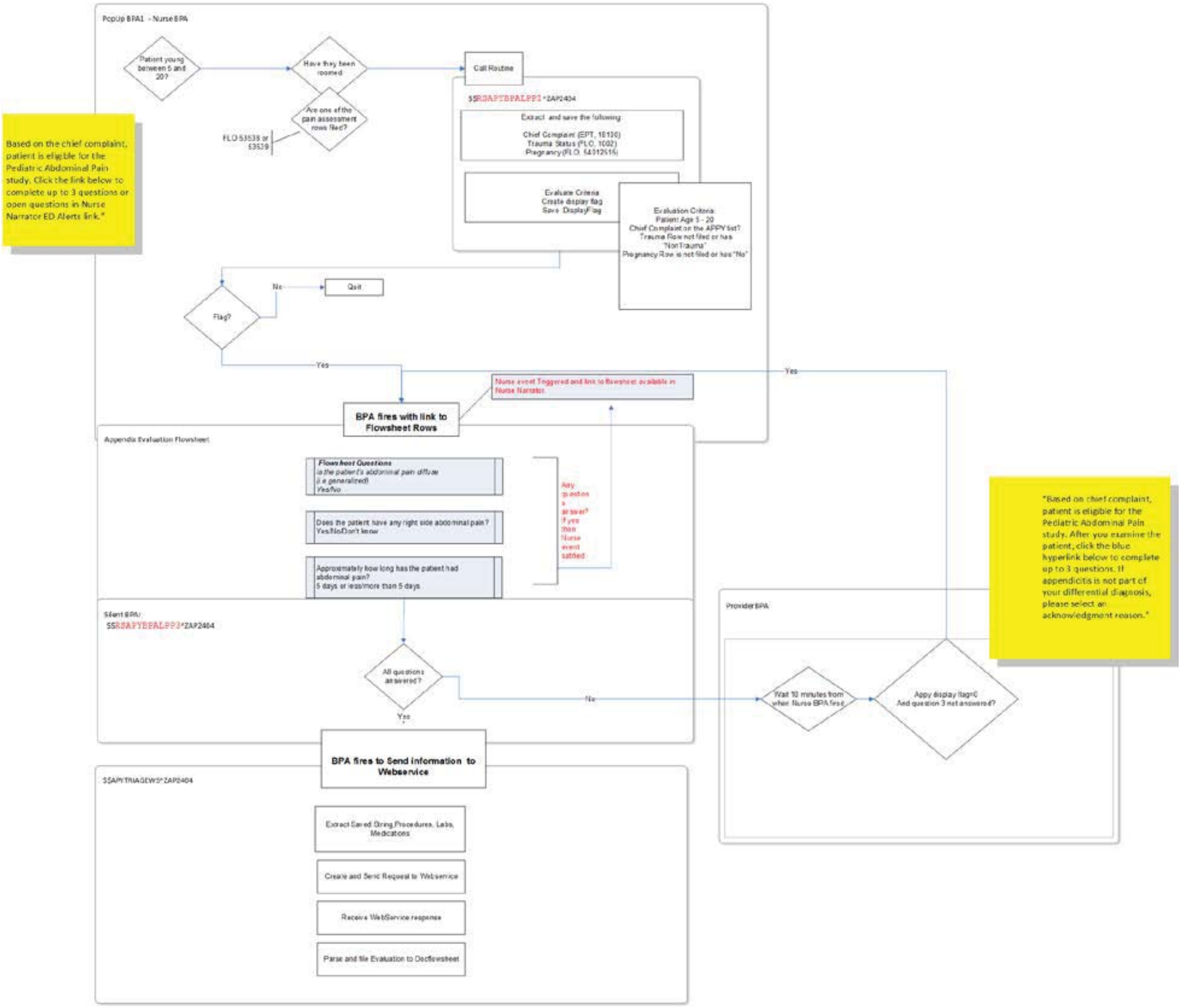

b) Workflow

For patients that meet the criteria described above, a Best Practice Advisory will display.

Best Practice Advisory prompting user for additional information.

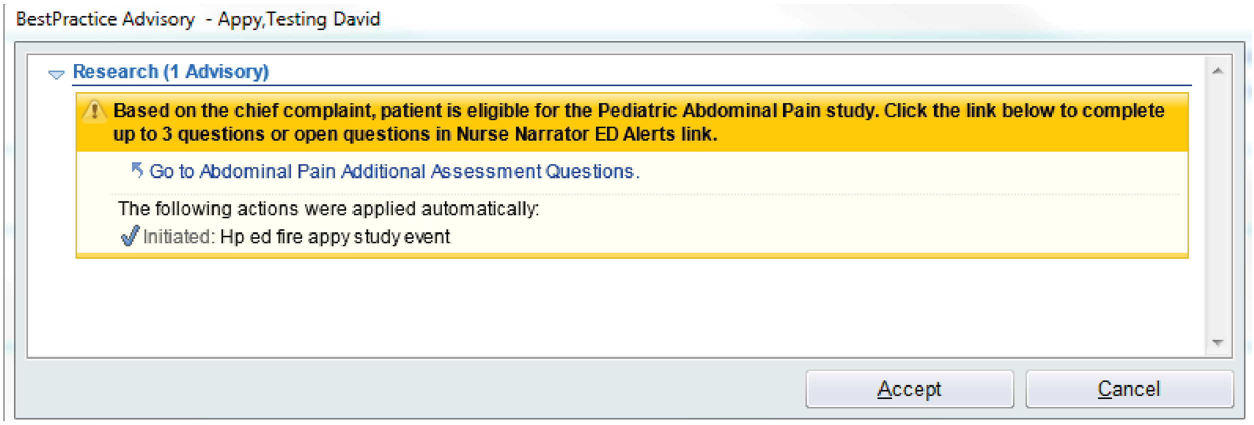

If the abdominal pain assessment link is clicked, the user will be directed to the event log in the nursing narrator section where up to 3 questions will be asked. The first two questions appear by default. The third question will appear if the answer to either 1 or 2 is yes. No further action is required by the user at this time other than closing the section.

Event log flowsheet questions for inclusion/eligibility.

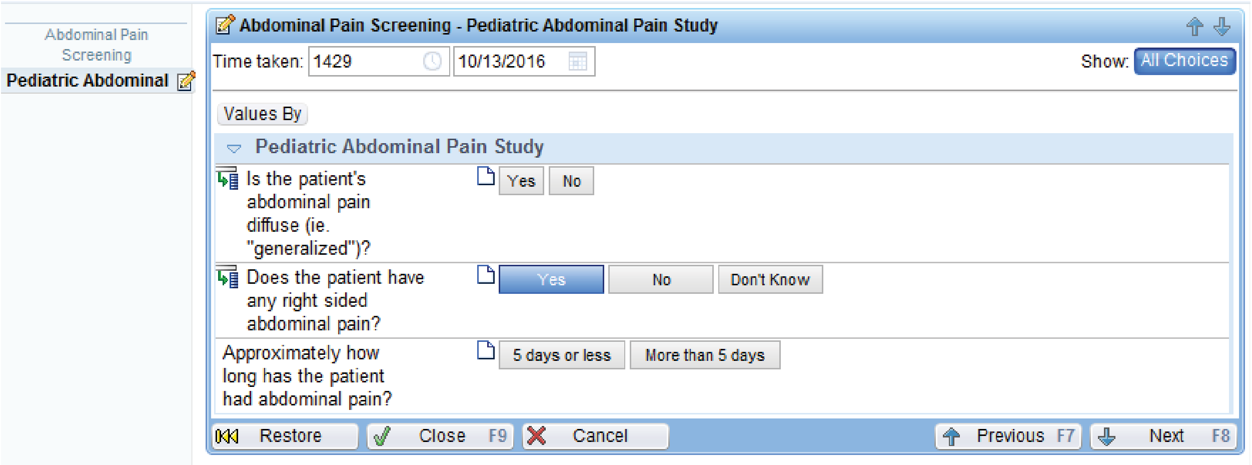

Once the flowsheet rows are completed, responses will be stored in Epic. This information will be retrieved at a later point for use in the full CDS for calculating appendicitis risk.

Additionally, if nurses do not respond to the Appy triage questions, a similar alert will fire at provider login. The Appy alert will display in the general BPA section of the EHR until the questions are addressed, or an acknowledgement reason is selected, as shown below.

Provider selects or enters reason for not completing triage questions.

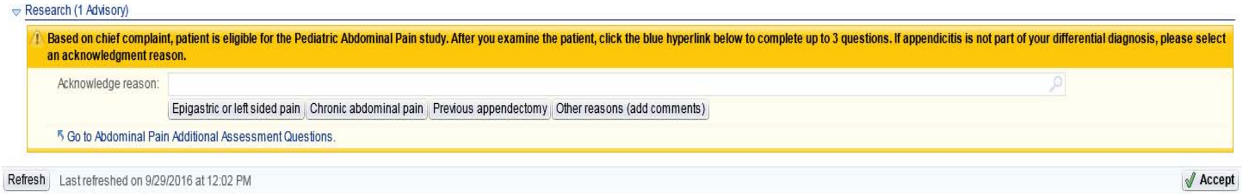

c) Full CDS-HP sites

The Full CDS is a multi-component, automated intervention. The Full CDS includes a collection of additional clinical parameters, including results of the CBC with differential (if available). The Full CDS assigns an exact "risk for appendicitis" and provides targeted recommendations regarding management.

Technical architecture of the Full CDS.

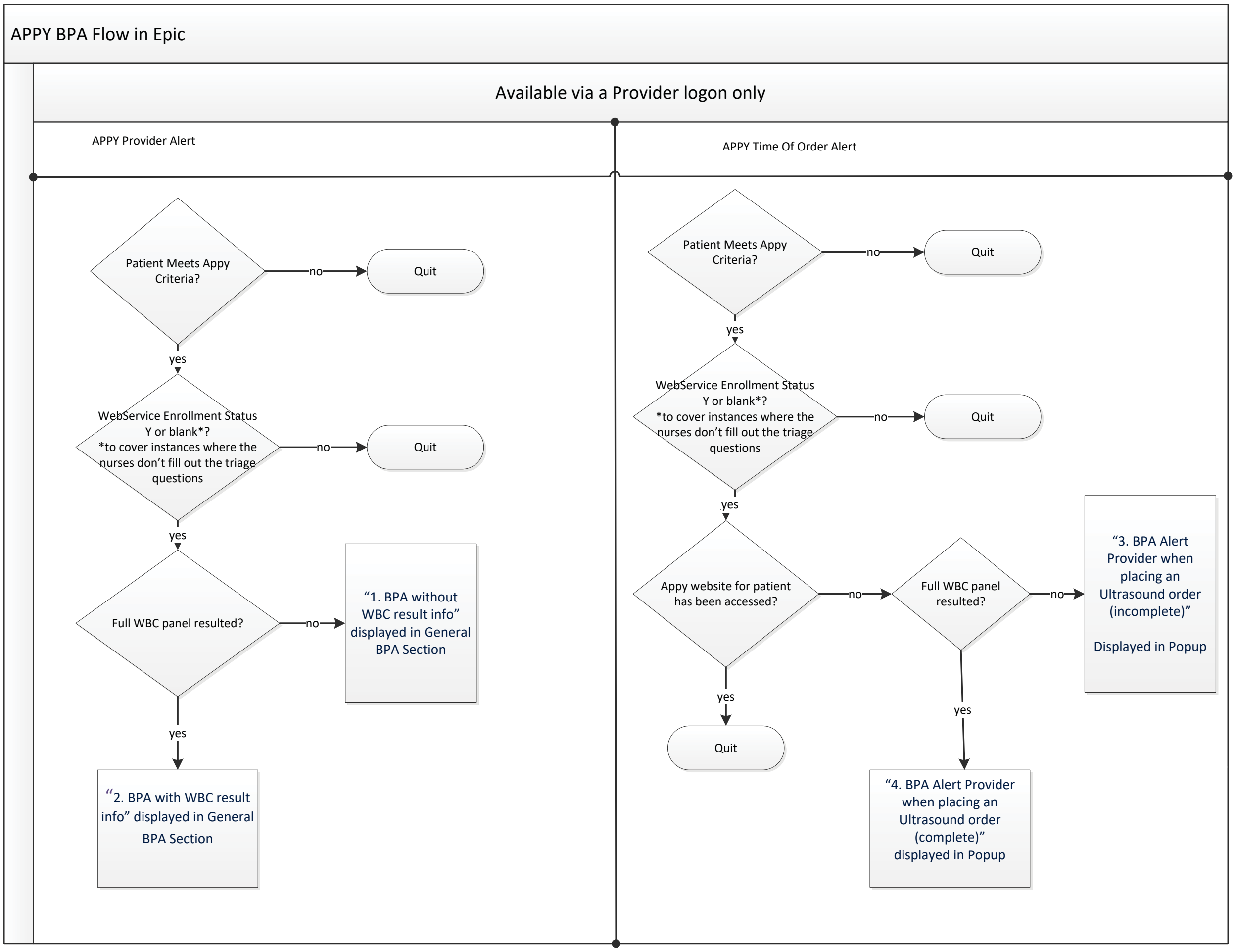

At the intervention clinics, if a patient meets the criteria described under the triage section, a BPA will display to the provider. The advisory prompts the user to click a link that will bring them to the full CDS that includes the risk prediction estimate (pARC score).

Best Practice Advisory prompting user to use the Full CDS.

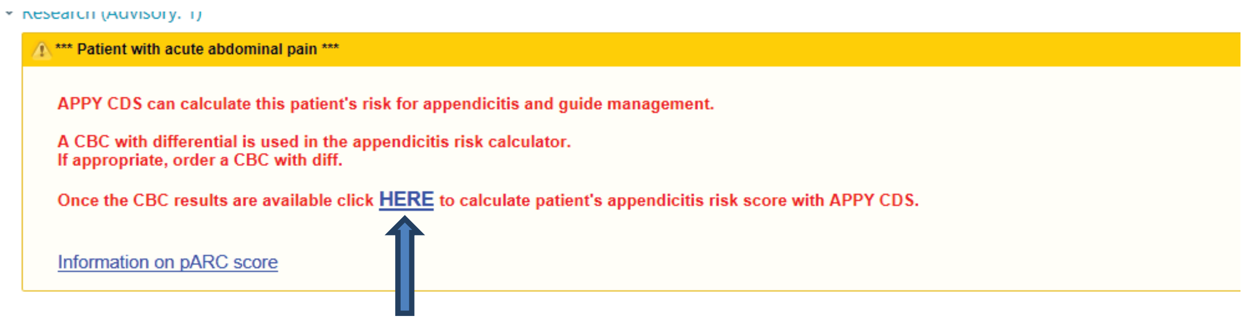

Once the link is clicked, the user is redirected to the Appy-CDS application. Below is the illustrated workflow.

Page 1- confirms eligibility and pulls in lab data, if available.

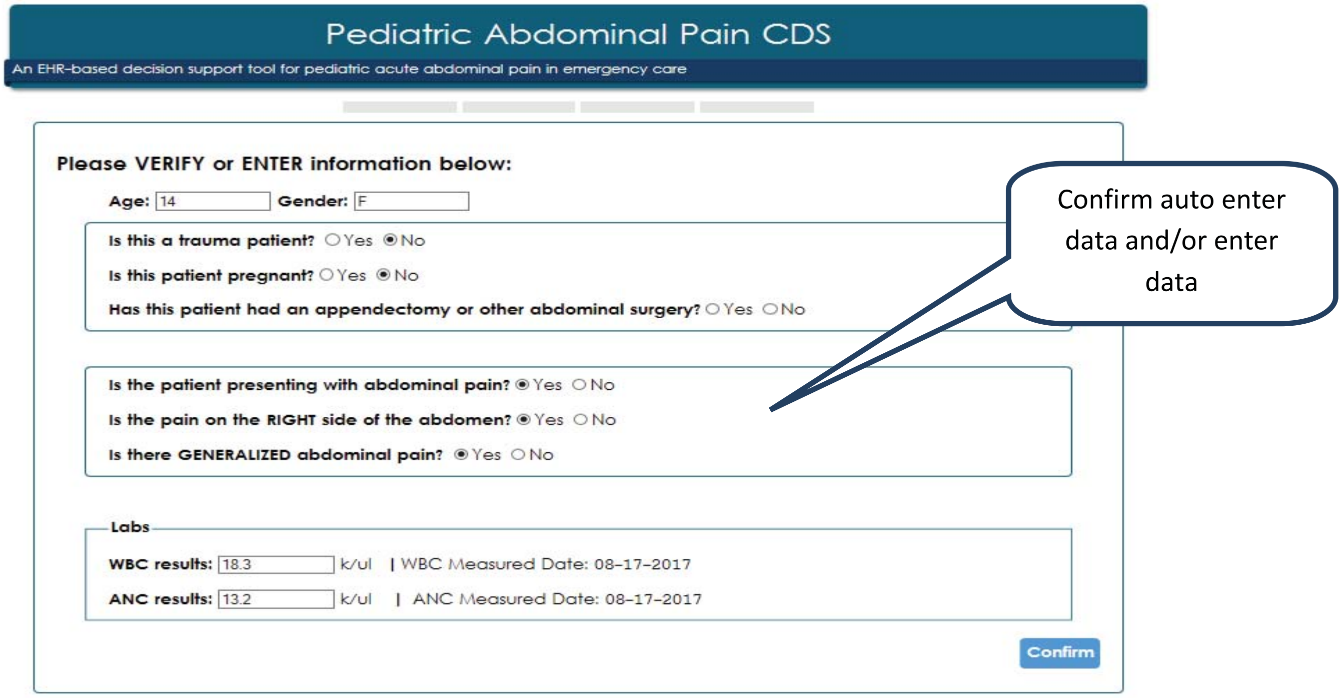

Page 2- questions regarding duration and location of pain.

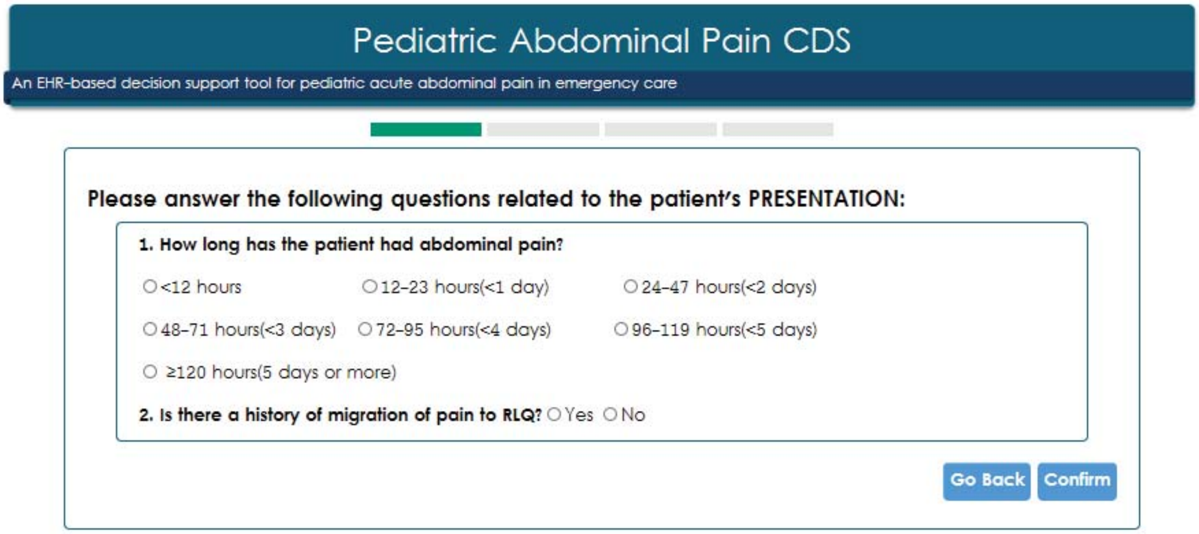

Page 3- questions on physical exam.

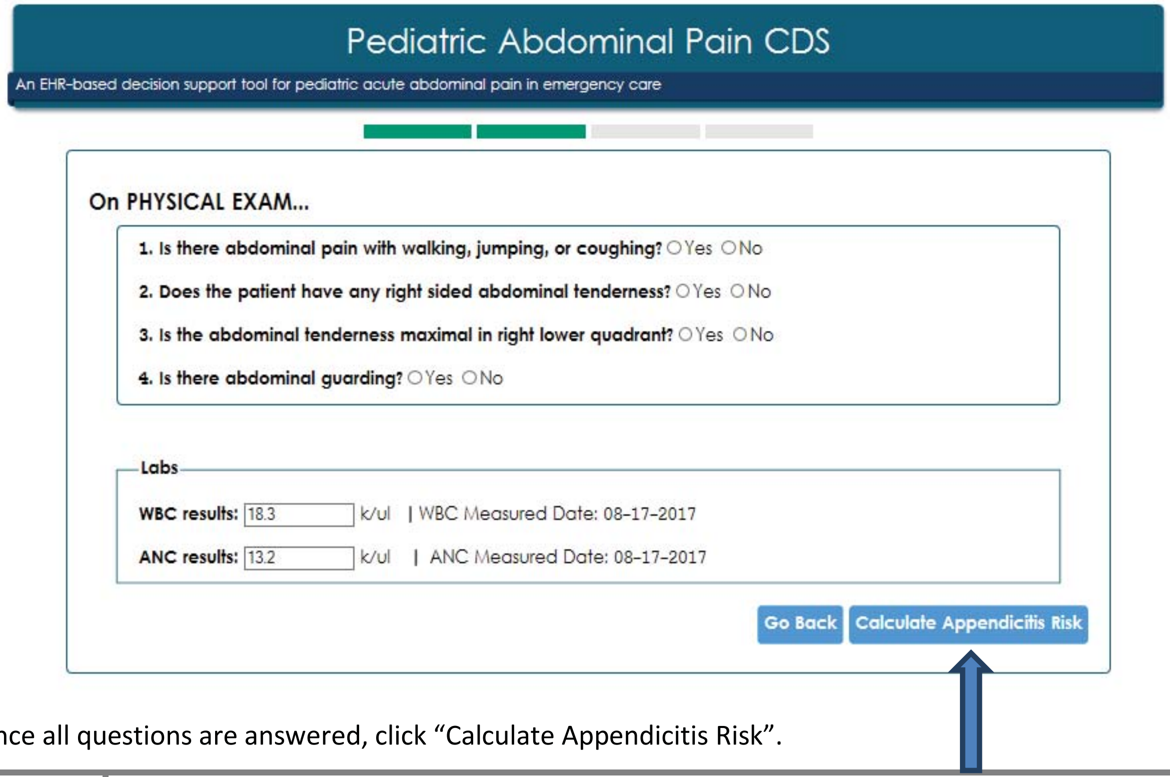

Once all questions are answered, click "Calculate Appendicitis Risk".

Page 4-Calculated risk of appendicitis using pARC score, summary, and management suggestions.

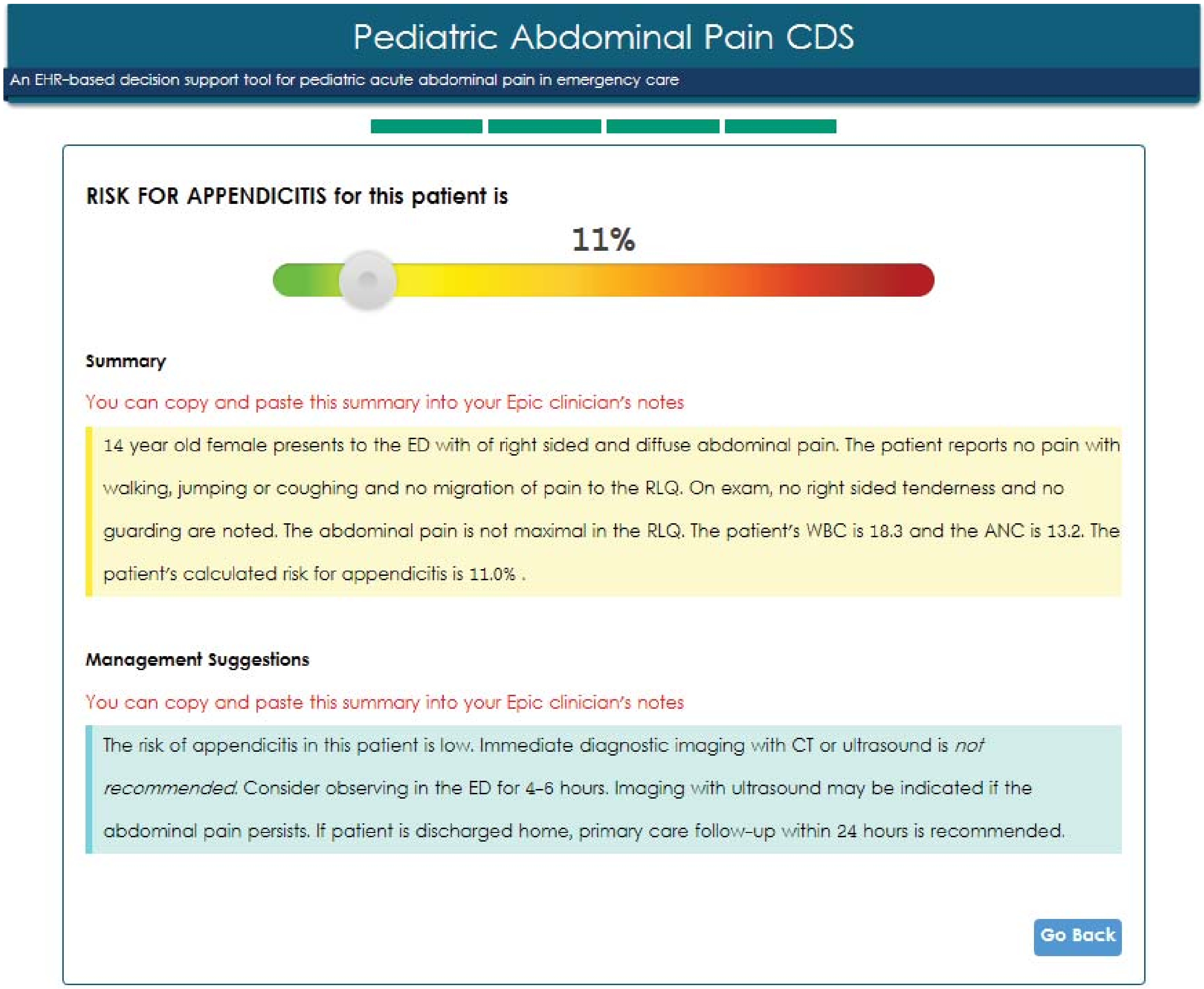

The summary and management suggestions can be copied into the provider note in the EHR. If lab data is not available for data extraction at the time the user accesses the CDS, the option to enter a theoretical WBC and ANC is available and a pARC score will be calculated based on the theoretical value. In some cases, there may be no data transferred from the EHR. In these instances, the user is able to enter data directly into the full CDS and calculate a pARC score.

To account for differences in workflow and the possibility that a provider may order imaging up front before utilizing the full CDS tool, there is an alert that displays when abdominal imaging is ordered. The imaging alert also includes a link to the full CDS and suggests to the user that using the pARC score is recommended before imaging. A screenshot of the imaging BPA is shown below.

Imaging alert for CDS prompting user for additional information.

> ***** Patient with acute abdominal pain *****
>
> If you are ordering a CT or US to evaluate for appendicitis, APPY CDS may be useful.
>
> A CBC with differential is used in the appendicitis risk calculator.
>
> Once the CBC with diff results are available and before placing an imaging order, click HERE to calculate patient's appendicitis risk using APPY CDS.
>
> Information on pARC score

d) Recommendations by Risk Group

The table below outlines the recommendations used in the HP full CDS by risk threshold.

Risk	Category	Proportion	Clinical recommendations
<5%	Ultra Low	37%	Outpatient, no diagnostic imaging
6-15%	Low	16%	ED observation for 4-6 hours; no imaging; outpatient follow-up
16-25%	Low moderate	10%	Pain <24hrs: observation x12 hrs, US and repeat CBC if no improvement<br><br>Pain ≥24 hrs, ultrasound
26-50%	Moderate	14%	Ultrasound recommended as first line imaging Admit for observation if equivocal
51-75%	Moderate	14%	Ultrasound recommended as first line imaging; CT if equivocal
76-90%	Moderate high	2%	Consult surgery, consider imaging
>90%	High	2%	Consult surgery, imaging not required

Appendix B: Intervention Design and Workflow- KP

a) Capturing eligibility: RISTRA

The analogous triage alert at KPNC will be implemented using the RISTRA interface existing at KPNC. For patients with a chief complaint of abdominal pain, ED providers will be trained to click on the RISTRA icon embedded within the EHR.

RISTRA can be accessed by clicking the icon within EHR.

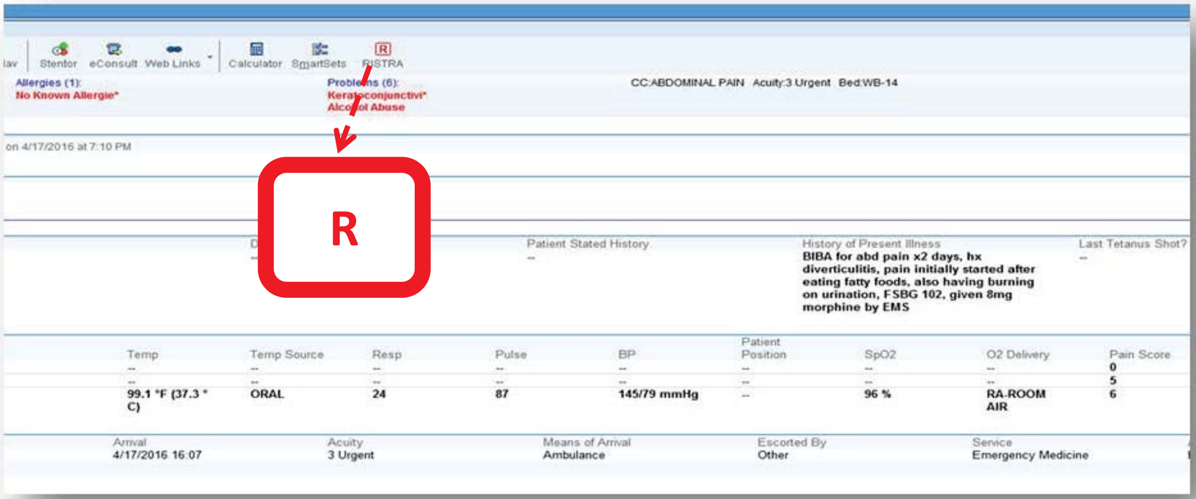

Once in the RISTRA tool, the user selects the project.

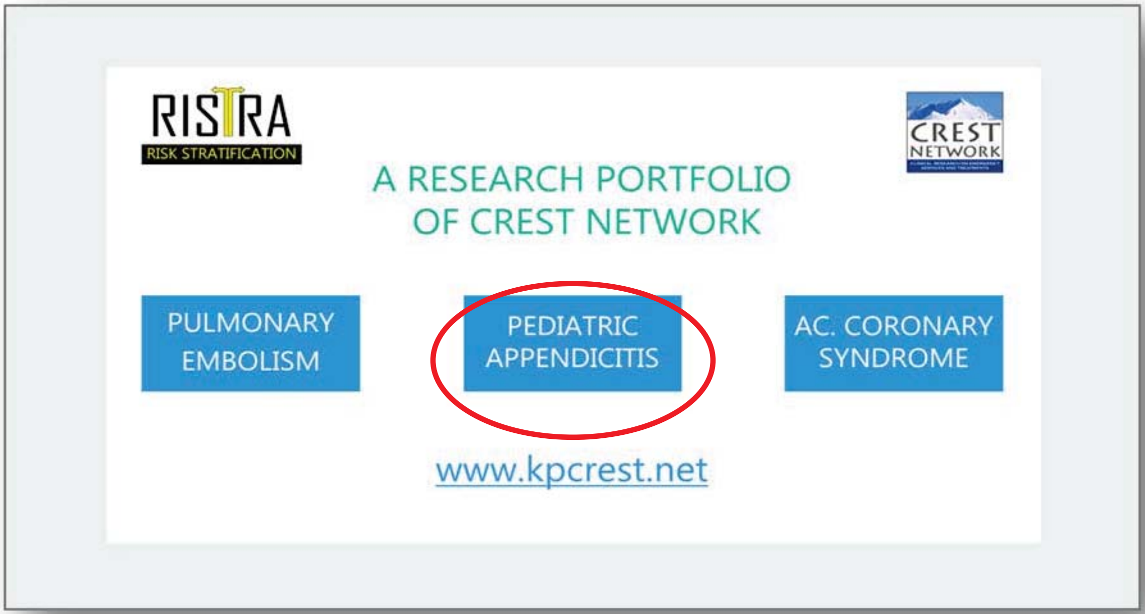

Page 1- Inclusion criteria, auto populated if available.

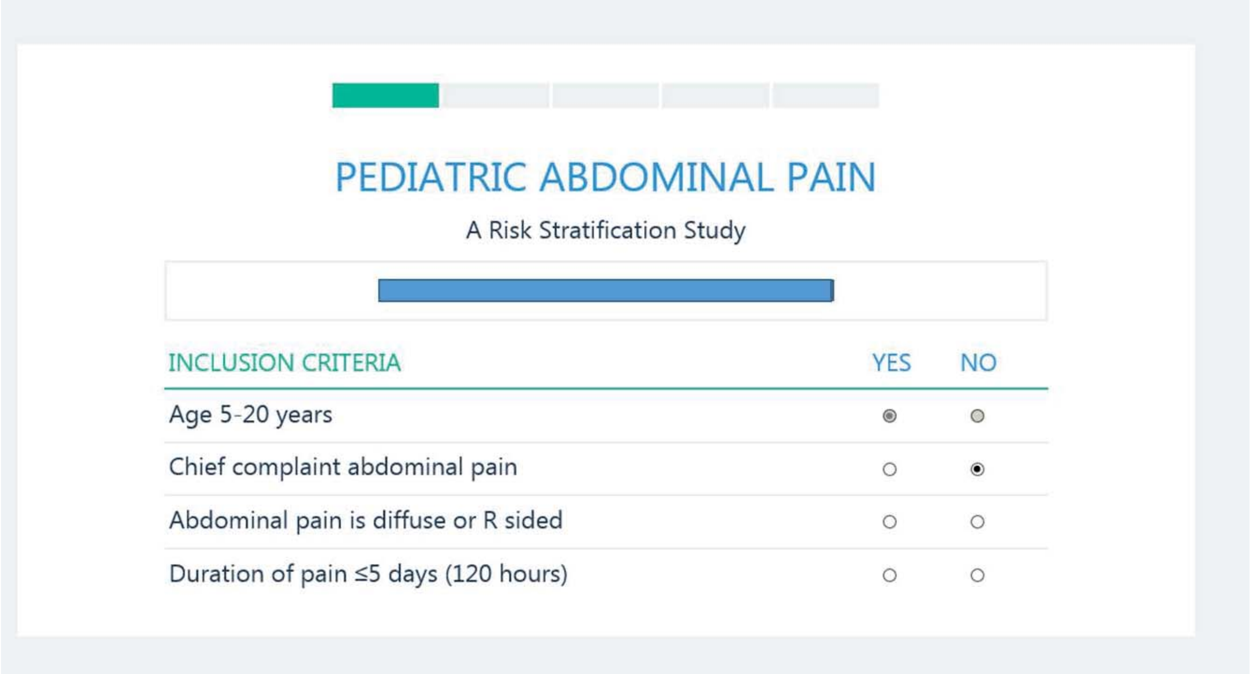

Page 2- Exclusion criteria, auto populated if available.

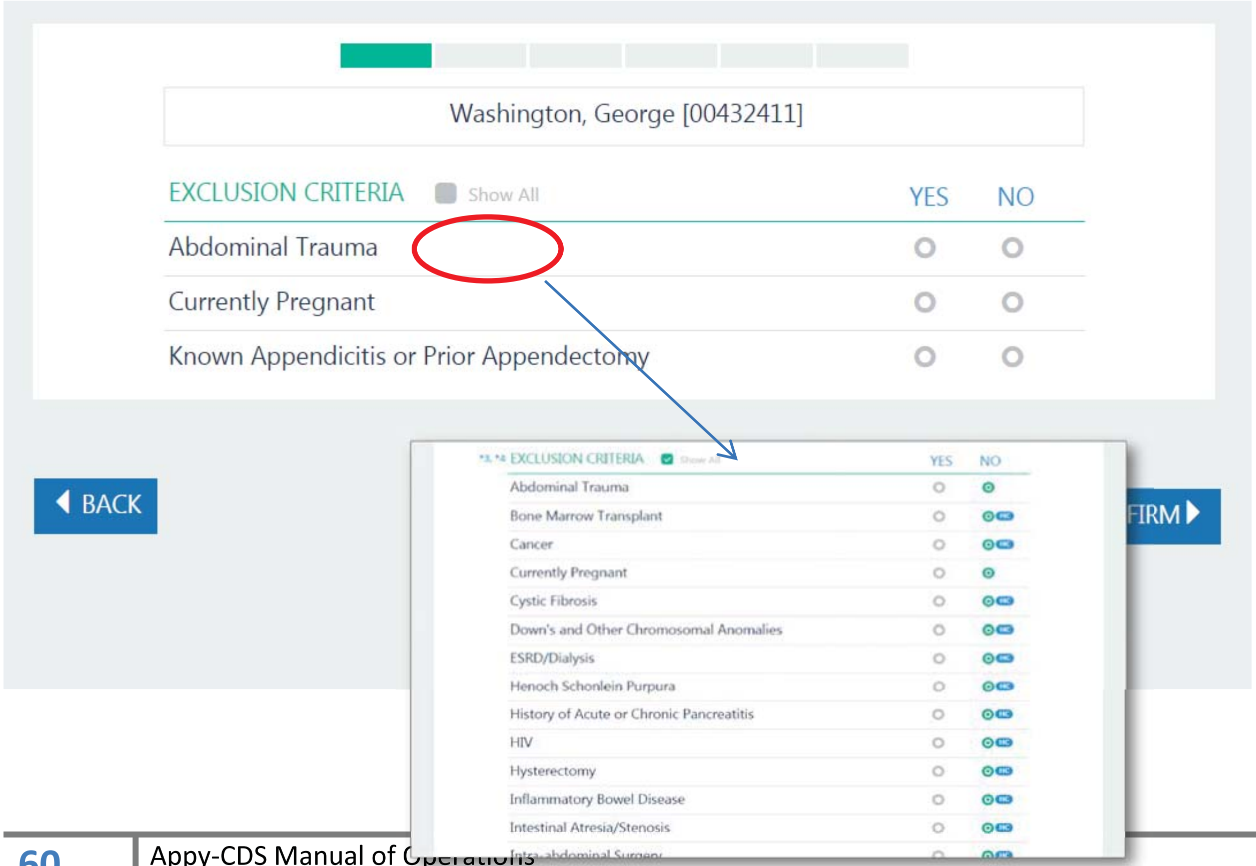

Page 3- questions regarding duration and location of pain, completed by clinician.

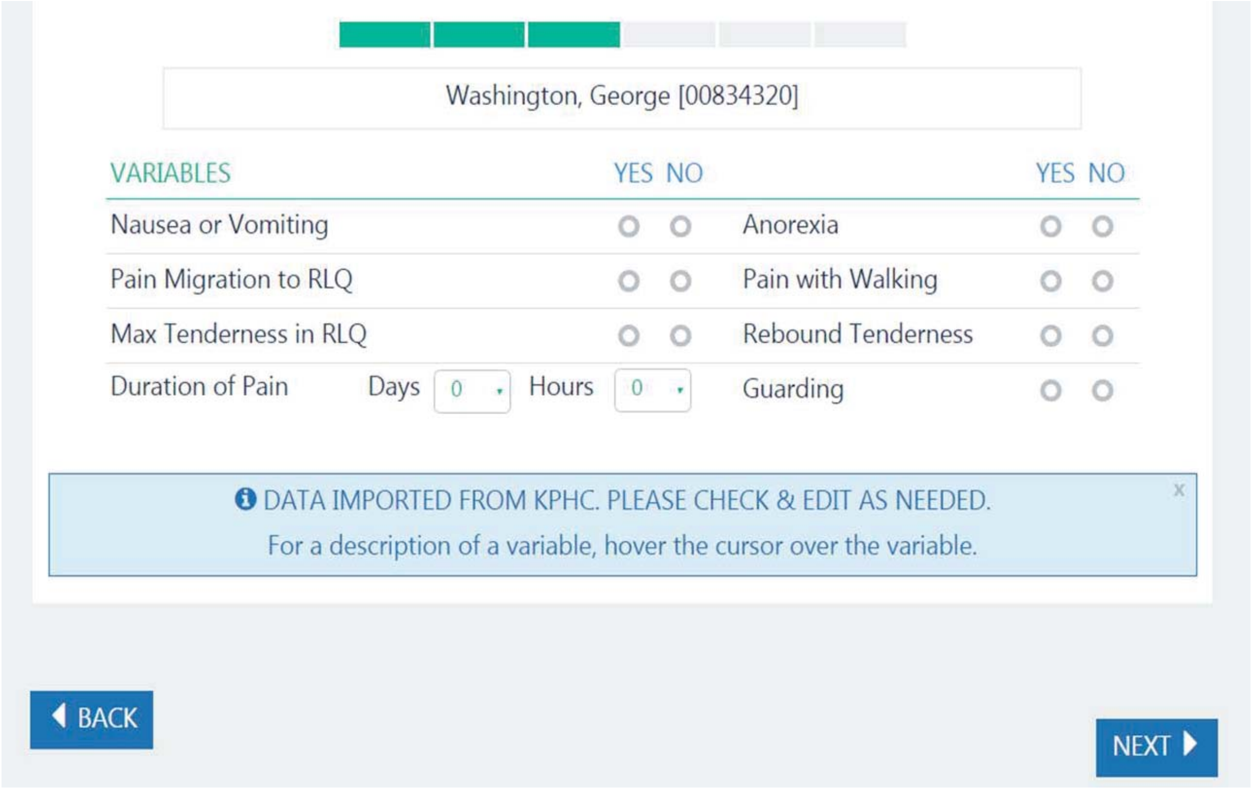

b) Full CDS-KPNC sites

Once eligibility is confirmed for the patient the clinician is asked to verify or complete additional variables about the patient's presentation (see below). The information is auto populated into the form, however required confirmation of accuracy by provider. The Full CDS at KP also involves provider gestalt to assign calculated risk of appendicitis as a pARC score. It provides targeted recommendations regarding management. This element of the CDS is unique to KP and will provide additional knowledge to the overall study goals.

The following diagram illustrates how recommendations are assigned based on the level of provider *Gestalt*. The provider is asked to use their clinical knowledge and expertise to assign *Gestalt.*

RISTRA CDS Provider Gestalt.

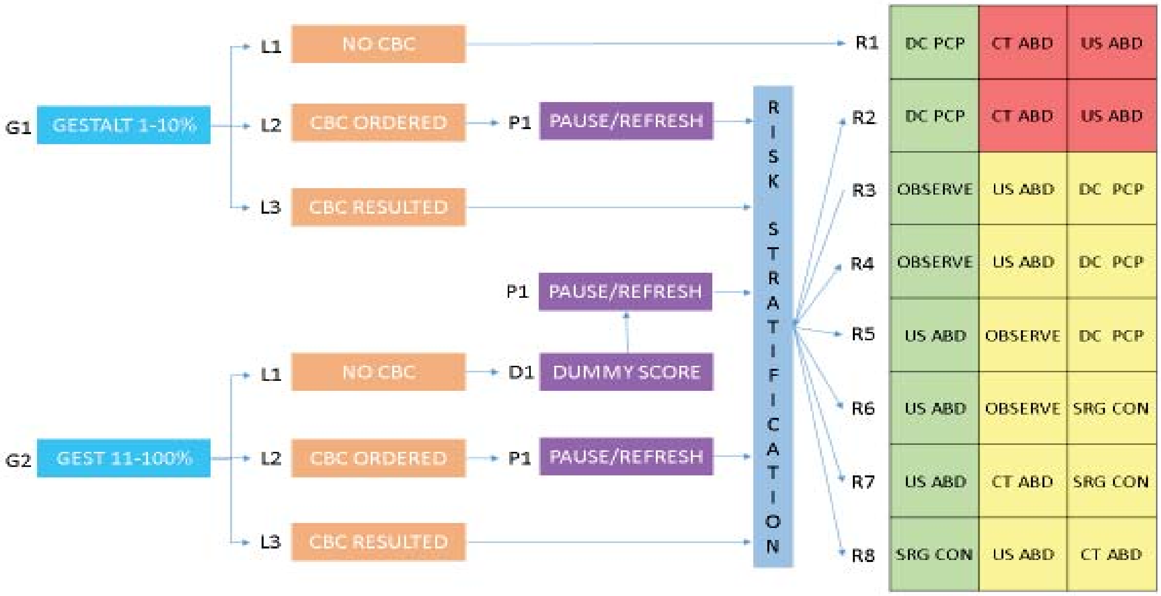

Once the clinician has determined a Gestalt score, they are prompted to use a sliding curser to mark the likelihood of the patient having appendicitis. The likelihood scale is presented as a percent (on a scale of 1-100). Once the clinician clicks calculate risk the CBC is pulled from the EHR.

Page 4- RISTRA CDS Percent Risk for Appendicitis.

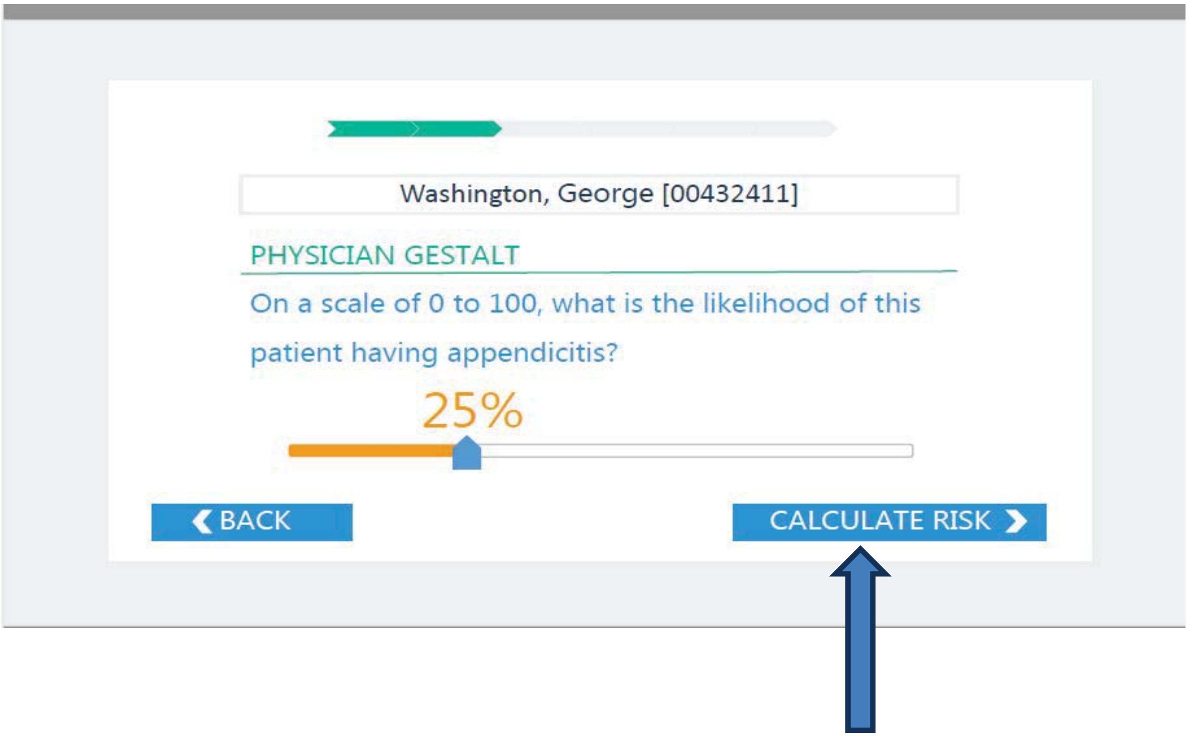

Page 5- RISTRA CDS calculated pARC score and recommendations.

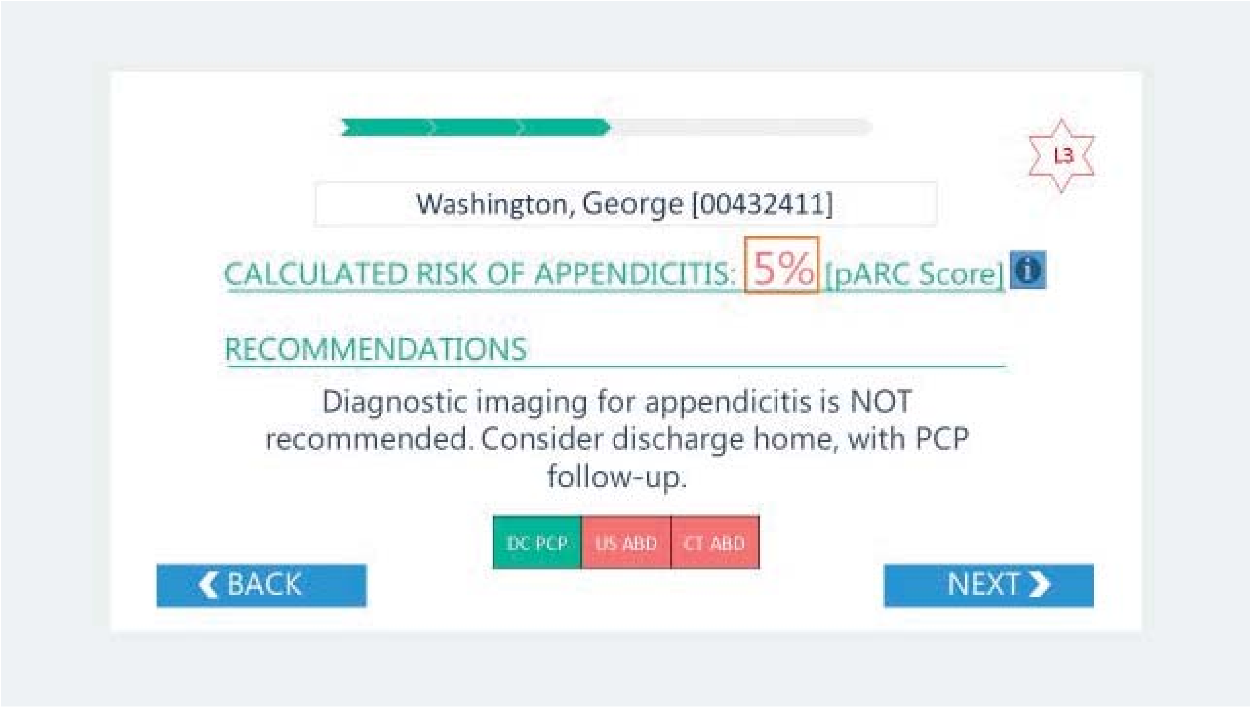

Page 6- RISTRA CDS Patient Summary can be copied and pasted into the EHR.

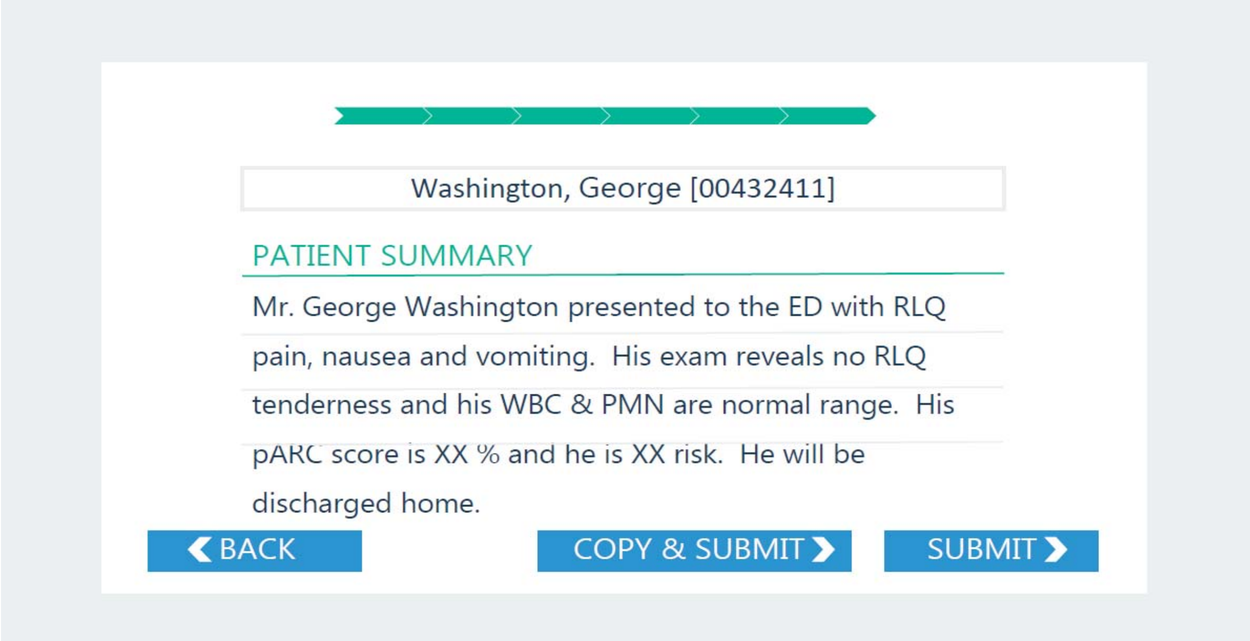

Similar to the design at HealthPartners, RISTRA has mechanisms in place for when the CBC has not been completed and results are not back. In these cases, the provider will see a form that gives them an option to refresh RISTA once the results are indicated in the EHR before they continue the CDS. The clinician also has the option to use a sliding scale to visualize what the pARC score might be using theoretical WBC values (see below). Once the CBC has resulted and the provider refreshes the RISTRA form, the provider can continue with the CDS to get the actual calculated risk of appendicitis.

RISTRA CDS Calculated Risk for Appendicitis with Hypothetical WBC.

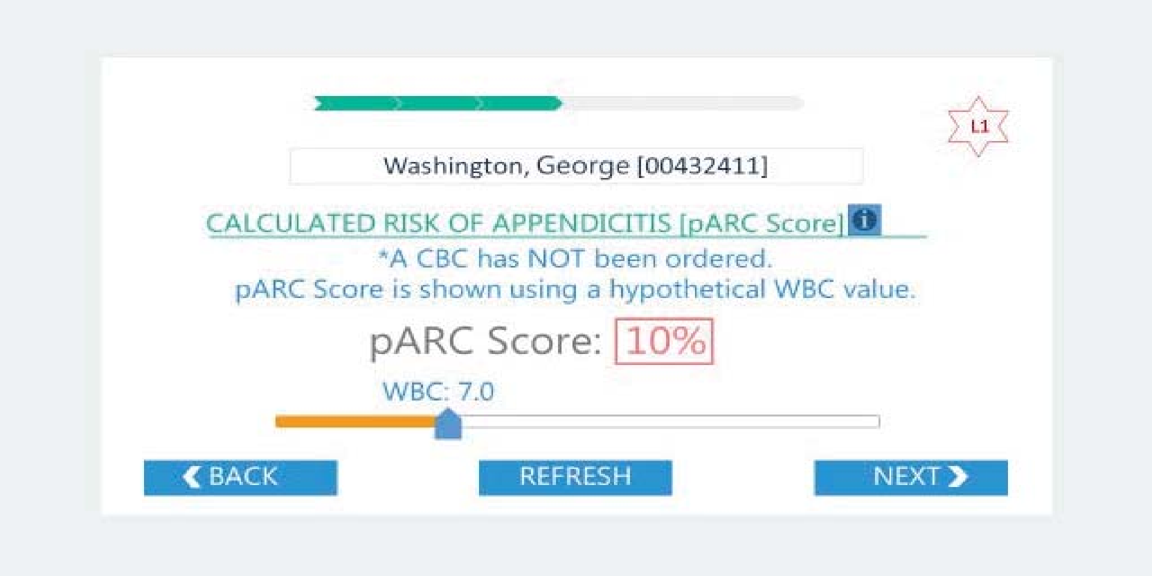

Provider can also gauge gestalt prior to ordering imaging.

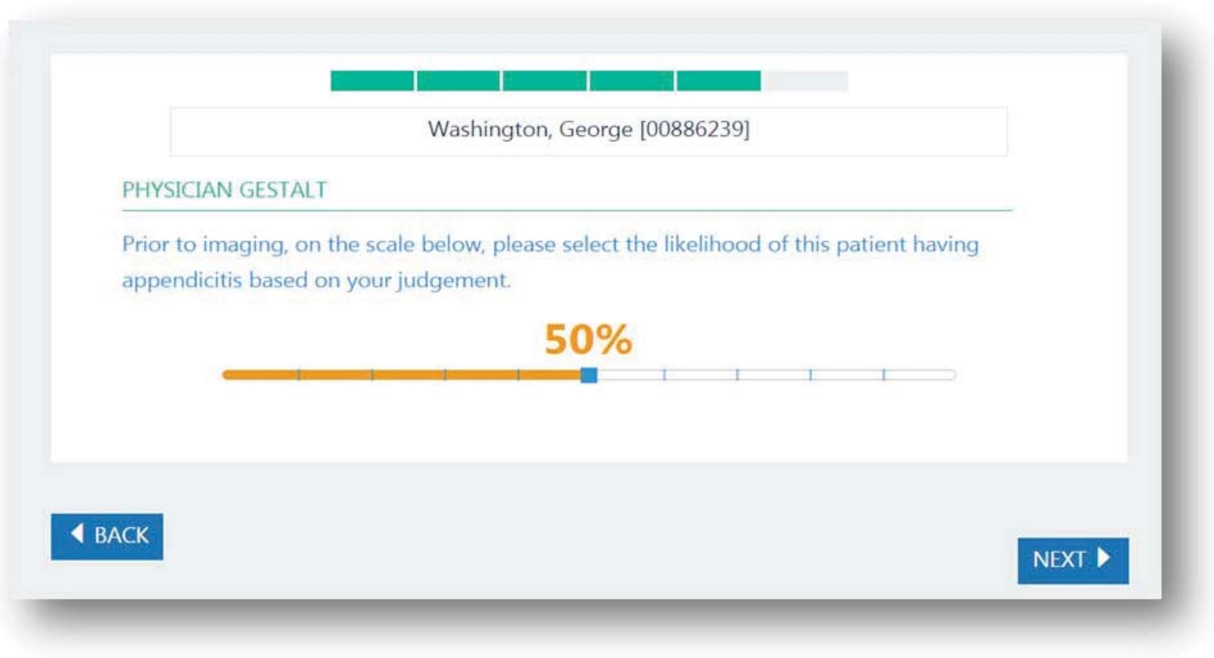

c) Recommendations by Risk Group

The below table describes recommendations used in the KP RISTRA full CDS by risk threshold.

MD Gestalt	Risk Stratification	Risk score	Clinical recommendations
<=10%	None	NA	Outpatient, no diagnostic imaging
>10%	Risk Stratification	<5%	No imaging; outpatient follow-up
		6-15%	Consider Observation; US if pain persists; outpatient follow-up
		16-25%	Pain +<24 hrs: Observation; US if pain persists<br><br>Pain >24 hours: US and/or surgery consult; Admit for observation if US equivocal
		26-50%	Ultrasound and/or surgery consults recommended as first line imaging; observation if US equivocal

		51-75%	US and / or surgery recommended, consider CT if US equivocal
		>75%	Consult surgery, imaging not routinely required

Appendix C: Reporting

a) Example enrollment report

For patients ages 5-<21, who have abdominal pain in any position.

Record ID (record_id)	Entered By (entered_by)	Site (site_name)	MM/DD/YYYY (month_reported)	Patients 5-20 years with abdominal pain as chief complaint (abdominal_pain)	Patient left prior to full evaluation (left_prior_eval)	Exclude an emergency department visit for abdominal pain if there i ... minal pain within prior 7 days (excl_prior_ed_visit)	Excluded due to trauma (excl_trauma)	Excluded due to pregnancy (excl_preg)	BPA did not fire (bpa_not_fired)	Excluded due to chronic illness or medication (see data dictionary for codes) (excl_illness)	Excluded due to prior appendectomy (see data dictionary for codes (excl_appendectomy)	Screening questions not completed (screening_not_compl)	Excluded based on screening questions (no right sided or diffuse pain or pain >5 days) (excl_screening)	ENROLLED (enrolled)
101_16_11	TD (1)	[redacted] (101)	11-01-2016	79	7	3	2	2	1	0	0	37	10	17
101_16_12	TD (1)	[redacted] (101)	12-01-2016	57	5	2	1	4	0	2	0	10	14	19
101_17_01	TD (1)	[redacted] (101)	01-01-2017	68	3	2	3	3	1	1	1	15	21	18
101_17_02	TD (1)	[redacted] (101)	02-01-2017	71	3	6	1	7	0	1	0	23	14	16
101_17_03	TD (1)	[redacted] (101)	03-01-2017	68	4	5	1	11	0	1	1	18	12	15
101_17_04	TD (1)	[redacted] (101)	04-01-2017	74	2	3	2	7	3	1	1	27	12	16

Appendix D: Data Collection Instruments

Appendicitis Chart Review Form for final outcomes- HealthPartners.

Appy CDS Chart Review
Page 1 of 3

HealthPartners

Study Id ______________________

Abstractor ID ______________________

Date of Abstraction ______________________

Imported Information

MRN ______________________

DOB : [dob]

Date of Index Emergency Department visit : [index_er_date]

Date of Index date + 7 days (pre-populated in Excel) : [index_7days]

Gender : [gender]

Site for index ED visit

- ○ Regions
- ○ Lakeview
- ○ Westfield
- ○ Hudson
- ○ Methodist
- ○ Amery

Patient disposition for index ED visit

- ○ Discharged home
- ○ Admitted to index (same) hospital
- ○ Admitted to other hospital (if yes indicate hospital from drop down list)
- ○ Other

if yes indicate hospital from drop down list

- ○ Amery
- ○ Children's Hospitals and Clinics of Minnesota - St. Paul
- ○ Children's Hospitals and Clinics of Minnesota - Minneapolis
- ○ Hudson Hospital
- ○ Lakeview Hospital
- ○ Methodist Hospital (PN)
- ○ Regions Hospital
- ○ Westfield Hospital
- ○ Other (please list)

Other (explain):

1. Between [index_er_date] (index date) and [index_7days] (7 days post index date) was the patient diagnosed with appendicitis?

- ◯ Yes
- ◯ No

2. Between [index_er_date] (index date), and [index_7days] (7 days post index date) did the patient undergo an appendectomy?

- ◯ Yes
- ◯ No

Hospital Course

(For patients going to the operating room or admitted to hospital during index ED visit)

3. Hospital where abdominal surgery occurred:

- ◯ Amery
- ◯ Children's Hospitals and Clinics of Minnesota - St. Paul
- ◯ Children's Hospitals and Clinics of Minnesota - Minneapolis
- ◯ Hudson Hospital
- ◯ Lakeview Hospital
- ◯ Methodist Hospital (PN)
- ◯ Regions Hospital
- ◯ Westfield Hospital
- ◯ Other (please list)

Other (please list): ______________________

4. Date of operation ______________________

5. Time of operation ______________________

6. Operative note findings:

- ◯ Normal (no evidence of appendicitis)
- ◯ Appendicitis (consistent with appendicitis, enlarged/inflamed appendix, dilated non-ruptured appendix, gangrenous appendix or likely appendicitis)
- ◯ Perforated Appendicitis (Ruptured appendix, abscess noted in the right lower quadrant, purulent material in abdomen, likely perforated appendicitis, or "complex appendicitis")
- ◯ Other (detail below)
- ◯ Note unavailable

Note operative findings:

7. Pathology report findings:

- ◯ Normal (No evidence for neutrophil invasion of tissue, lymphoid hyperplasia present with no evidence for appendicitis, no serosal injection present)
- ◯ Appendicitis (includes the following report terms; "consistent with appendicitis", "non-perforated appendicitis", "fecalith present with neutrophil invasion of tissue", "acute peri-appendicitis", "acute appendicitis", "no evidence of perforation noted")
- ◯ Perforated Appendicitis (Micro-perforation, abscess formation noted, frank perforation noted)
- ◯ Other (detail below)
- ◯ Note unavailable

Confidential

Note pathology findings:

Appendicitis Chart Review Form - Children's Minnesota

Confidential

Childrens

Study Id ______________________

Children's Id ______________________

Imported Information

Date of Index Emergency Department visit : [index_er_date]

Date of Index date + 7 days (pre-populated in Excel) : [index_7days]

Gender : [gender]

HealthPartner's date of discharge ______________________ (MDY)

Which HP hospital was patient at?
- ○ Regions
- ○ Lakeview
- ○ Westfield
- ○ Hudson
- ○ Methodist
- ○ Amery

Was patient transferred to Children's?
- ○ Yes
- ○ No

(Came to Children's from HP facility within 1 day)

Date and time of Children's ED visit ______________________ (MDY H:M)

How did the patient arrive at Children's ED?
- ○ Car
- ○ EMS
- ○ Public Transportation
- ○ Other

If other, how did the patient arrive? ______________________

Did the patient have surgery during this visit?
- ○ Yes
- ○ No

What date and time was the operation? ______________________ (MDY H:M)

Did the patient have surgery for appendicitis during this visit?
- ○ Yes
- ○ No

What date and time was the surgery for appendicitis preformed? ______________________ (MDY H:M)

Was this a negative appendectomy?

(from the pathology report)
- ○ Yes
- ○ No

(Key Words for Negative Appendectomy: No evidence for neutrophil invasion of tissue, lymphoid hyperplasia present with no evidence for appendicitis, no serosal injection present)

What does pathology report indicate? ______________________

Final Diagnosis for this visit

- ◯ Appendicitis
- ◯ Perforated Appendicitis (from operative note)
- ◯ Medical Diagnosis
- ◯ Other Surgical

(Perforation Key Words:Ruptured appendix, abscess noted in the right lower quadrant, purulent material in abdomen, likely perforated appendicitis, or "complex appendicitis")

What did the operative note indicate?

____

(Perforation Key Words: Ruptured appendix, abscess noted in the right lower quadrant, purulent material in abdomen, likely perforated appendicitis, or "complex appendicitis")

Which medical diagnosis

- ◯ Non-specific abdominal Pain
- ◯ Constipation
- ◯ Gastritis
- ◯ Gastroenteritis
- ◯ Mesenteric Adenitis
- ◯ PID
- ◯ Renal Stone
- ◯ UTI
- ◯ Other Medical Diagnosis

Did the patient have any of the following surgical emergencies during this visit?

- ☐ Ovarian torsion
- ☐ Testicular torsion
- ☐ Abdominal tumor
- ☐ Intussusception
- ☐ Volvulus
- ☐ Ectopic pregnancy
- ☐ N/A

(Check all that apply)

Did the patient receive imaging at Children's during this visit?

- ◯ Yes
- ◯ No

What imaging studies did the child receive?

- ☐ Xray
- ☐ Ultrasound
- ☐ CT
- ☐ MRI

Did any of the following medical emergencies occur during this visit?

- ☐ Small bowel obstruction
- ☐ Tub-ovarian abscess
- ☐ Pelvic inflammatory disease
- ☐ Pyelonephritis

Diagnosis code 1 __________________________________

Diagnosis Description 1 __________________________________

Diagnosis code 2 __________________________________

Diagnosis Description 2 __________________________________

Diagnosis code 3 __________________________________

Diagnosis Description 3 __________________________________

Diagnosis code 4 __________________________________

Diagnosis Description 4 __________________________________

Patient Disposition for this visit

- ◯ Discharged Home
- ◯ Sent to the OR
- ◯ Admitted to Children's inpatient
- ◯ Transferred to an outside facility
- ◯ Patient death

(Please choose one)

What day and time was the patient discharged from the ED?

____
(MDY H:M)

Notes

____

Return Visit Information

Did the patient return to Children's within 7 days?

- ◯ Yes
- ◯ No

How many times did the patient return to Children's within 7 days? (Do not include a transfer)

- ◯ 1 return visit
- ◯ 2 return visits
- ◯ 3 return visits
- ◯ 4 return visits
- ◯ 5 return visits
- ◯ 6 return visits
- ◯ 7 return visits

(This number of visits does not include a transfer to Children's)

Visit 1

Date and time of Children's return visit

____
(MDY H:M)

Where did visit take place?

- ◯ Clinic
- ◯ ED
- ◯ Inpatient
- ◯ ICU

(Highest Acuity)

How did the patient arrive at Children's?

- ◯ Car
- ◯ EMS
- ◯ Public Transportation
- ◯ Other

If other, how did the patient arrive?

____

Did the patient have surgery during this visit?

- ◯ Yes
- ◯ No

What date and time was the operation?

____
(MDY H:M)

Did the patient have surgery for appendicitis during this visit?

- ◯ Yes
- ◯ No

What date and time was the surgery for appendicitis preformed?

____
(MDY H:M)

Was this a negative appendectomy?<br>(from the pathology report)	◯ Yes<br>◯ No<br>(Key Words for Negative Appendectomy: No evidence for neutrophil invasion of tissue, lymphoid hyperplasia present with no evidence for appendicitis, no serosal injection present)
What does pathology report indicate?	________________________
Final Diagnosis for this visit	◯ Appendicitis<br>◯ Perforated Appendicitis (from operative note)<br>◯ Medical Diagnosis<br>◯ Other Surgical<br>(Perforation Key Words:Ruptured appendix, abscess noted in the right lower quadrant, purulent material in abdomen, likely perforated appendicitis, or "complex appendicitis")
What did the operative note indicate?	________________________<br>(Perforation Key Words: Ruptured appendix, abscess noted in the right lower quadrant, purulent material in abdomen, likely perforated appendicitis, or "complex appendicitis")
Did the patient have any of the following surgical emergencies during this visit?	☐ Ovarian torsion<br>☐ Testicular torsion<br>☐ Abdominal tumor<br>☐ Intussusception<br>☐ Volvulus<br>☐ Ectopic pregnancy<br>☐ N/A<br>(Check all that apply)
Which medical diagnosis	◯ non-specific abdominal Pain<br>◯ Constipation<br>◯ Gastritis<br>◯ Gastroenteritis<br>◯ Mesenteric Adenitis<br>◯ PID<br>◯ Renal Stone<br>◯ UTI<br>◯ Other Medical Diagnosis
Did the patient receive imaging at Children's this visit?	◯ Yes<br>◯ No
What imaging studies did the child receive?	☐ Xray<br>☐ Ultrasound<br>☐ CT<br>☐ MRI
Did one of the following medical emergencies occur?	☐ Small bowel obstruction<br>☐ Tub-ovarian abscess<br>☐ Pelvic inflammatory disease<br>☐ Pyelonephritis
Diagnosis code 1	________________________
Diagnosis Description 1	________________________
Diagnosis code 2	________________________
Diagnosis Description 2	________________________

Diagnosis code 3	________________
Diagnosis Description 3	________________
Diagnosis code 4	________________
Diagnosis Description 4	________________
Patient Disposition	◯ Discharged Home<br>◯ Sent to the OR<br>◯ Admitted to Children's inpatient<br>◯ Transferred to an outside facility<br>◯ Patient death<br>(Please choose one)
What date and time was the patient discharged?	________________<br>(MDY H:M)

Visit 2

Date and time of Children's return visit	________________<br>(MDY H:M)
Where did visit take place?	◯ Clinic<br>◯ ED<br>◯ Inpatient<br>◯ ICU<br>(Highest Acuity)
How did the patient arrive at Children's?	◯ Car<br>◯ EMS<br>◯ Public Transportation<br>◯ Other
If other, how did the patient arrive?	________________
Did the patient have surgery during this visit?	◯ Yes<br>◯ No
What date and time was the operation?	________________<br>(MDY H:M)
Did the patient have surgery for appendicitis during this visit?	◯ Yes<br>◯ No
What date and time was the surgery for appendicitis preformed?	________________<br>(MDY H:M)
Was this a negative appendectomy?<br>(from the pathology report)	◯ Yes<br>◯ No<br>(Key Words for Negative Appendectomy: No evidence for neutrophil invasion of tissue, lymphoid hyperplasia present with no evidence for appendicitis, no serosal injection present)
What does pathology report indicate?	________________

***Children's chart review form repeats for up to 7 visits.**

Appendix E: Data Dictionary

List of variables for Hypothesis 1.

Variable Name	Variable Type and Length (bytes)	Values	Variable description
Study_ID	char(7)	HP00001, HP00002, etc<br>KP00001, KP00002, etc	Study id
Site<br><br>Intervention_arm	num(8)	101=Regions,<br>102=Lakeview,<br>103=Westfield,<br>104=Hudson,<br>105=Methodist,<br>106=Amery<br>201=Sacramento,<br>202=Roseville,<br>203=South San Francisco,<br>204=San Rafael,<br>205=Santa Clara,<br>206=South Sacramento,<br>207=San Leandro,<br>208=Fremont,<br>209=Oakland,<br>210=Richmond,<br>211=San Francisco	Site of Emergency Department
ED_Arrival_DT	num(8)	sas date	ED Arrival Date
ED_Arrival_TM	num(8)	sas time	ED Arrival Time

ED_Arrival_Means	num(8)	1=Car,<br>2=Ambulance,<br>3=Police,<br>4=Helicoptor,<br>5=Other,<br>6=Unknown,<br>7=Newborn,<br>8=Public Transportation,<br>9=Taxi,<br>10=Walk,<br>11=Dropped Off,<br>12=Light Rail,<br>54=Hospital Transport	Means of Arrival to the ED (follow Epic values in ZC_ARRIV_MEANS table)
Age	num(8)	5-20	Age at ED Arrival (in years)
Pat_Enc_CSN_ID	num(8)		Patient Encounter ID that uniquely identifies an ED visit
Prov_ID	char(18)		Provider ID
Prov_Type	num(8)	1=Physician,<br>2=Resident,<br>3=Physician Assistant,<br>4=Nurse Practitioner	Provider Type
Insur_Type	num(8)	1=Commercial,<br>2=Medicaid,<br>3=Other,<br>4=None	Type of medical insurance at time of ED visit
Prior_ED_Visit	num(8)	1=Prior ED visit for abdominal pain in the past 7 days,<br>0=no prior ED visit for reason of interest in past 7 days	Designates if had prior ED visit at one of study sites for reason of interest in past 7 days within the same health care system. No need to further track as patient followed based on index visit.
Pregnant_CDS	num(8)	1=Pregnant based on CDS<br>0=Not pregnant	Designates if patient is pregnant at time of ED visit and will be excluded

Pregnant_Dx	num(8)	1=Pregnant based on diagnoses codes on day of ED visit<br>0=Not pregnant	ICD10: O00-O99, O9A.x
Trauma_CDS	num(8)	1=Trauma based on CDS, (included TTA and no TTA)<br>0=No trauma	Designates if trauma patient at time of ED visit and will be excluded
Trauma_Dx	num(8)	1=Trauma based on diagnoses on day of ED visit, (included TTA and no TTA)<br>0=No trauma	See Trauma_Dxs tab, have one of these dxs on day of ED Visit
Prior_Appendicitis	num(8)	1=Prior appendicitis,<br>0=No prior appendicitis	Designates if patient ever had prior appendicitis based on any available data source (ICD-9 and ICD-10 diagnoses, appendectomy procedure codes, etc) See Appendicitis_Codes tab.
Patient_Roomed	num(8)	1=Patient roomed,<br>0=Patient not roomed	Designates if patient was roomed
ED_Roomed_DT	num(8)	sas date	ED Roomed Date
ED_Roomed_TM	num(8)	sas time	ED Roomed Time
Pain_Scale_Avail	num(8)	1=Pain scale available,<br>0=Pain scale not available	Designates if pain scale available at time of ED visit
Pain_Scale_First	num(8)		First pain scale available for this ED visit
Pain_Scale_First_TM	num(8)		Time of first pain schale available for this ED Visit
AppyCDS_triage_display	num(8)	1=APPY CDS triage alert fired and displayed,<br>2=APPY CDS triage did not fire<br>3=APPY CDS triage fired but did not display	Designates if APPY CDS triage alert fired and/or displayed at time of ED visit
APPYCDS_triage_TM	num(8)	sas time	Time the CDS triage displayed (if displayed)

Right_Side_Pain_triage	num(8)	1=Any right sided abdominal pain reported from triage,<br>0=No right sided abdominal pain reported from triage	Designates if any right sided abdominal pain reported by RN or MD in response to triage alert
Pain_diffuse	num(8)	1=Yes, there is diffuse or generalized abdominal pain,<br>0=No, the pain is not diffuse or generalized	Designates if the pain is diffuse or generalized
Pain_5days_or_less	num(8)	1=Pain duration 5 days or less, reported from triage<br>0=Pain duration more than 5 days, reported from triage	Designates if pain duration is 5 days or less at time of ED visit
Eligible_Triage	num(8)	1=Eligible,<br>0=Not eligible	Designates if patient is eligible from triage at time of ED visit. Derived variable from Right_Side_Pain, Pain_diffuse and PAIN_5days_or_less variables.
Eligible_Meds	num(8)	1=Eligible based on medication criteria,<br>0=Not eligible based on medication criteria	Designates if patient is eligible based on medication criteria
Eligible_Chronic	num(8)	1=Eligible based on chronic condition criteria,<br>0=Not eligible based on chronic condition criteria	Designates if patient is eligible based on chronic condition criteria
MD_acknowledge_BPA	num(8)	43=No right-sided or diffuse abdominal pain<br>5=Chronic abdominal pain<br>8=Previous appendectomy<br>18=Other reasons (add comments)	MD acknowledge that patient not eligible

ED_Disposition	num(8)	1=Admit Inpt, 2=Home,<br>3=Transfer to Other Hospital,<br>4=Expired, 5=AMA,<br>6=Clinic,<br>7=Detox, 8=Jail,<br>9=LWBS/LWBF,<br>10=Observation,<br>11=Shelter, 12=NH/TCU,<br>13=Error,<br>14=OR/SDS/L&D (non-admit),<br>15=LWBR, 16=Current Admit,<br>17=Home,<br>18=Hospital Bed,<br>19=Observation,<br>20=Detox, 21=AMA, 22=Clinic,<br>23=Transfer to Other Hospital,<br>24=Jail,<br>25=LWBS/LWBF, 26=Expired,<br>27=Shelter, 28=NH/TCU,<br>29=Error, 30=Surgery/Dialysis,<br>31=Not Registered (LWBR),<br>32=Current Admit,<br>33=Cancel Expected Patient,<br>34=LWBS Before Triage,<br>35=Left Without Being Finished,<br>36=L&D, 40=LWBS After Triage	Disposition of the patient when discharged from the ED
ED_Discharge_DT	num(8)	sas date	ED Discharge Date
ED_Discharge_TM	num(8)	sas time	ED Discharge Time
CT_order	num(8)	1=Yes<br>0=No	CT of abdomen ordered for this ED visit
US_order	num(8)	1=Yes<br>0=No	Ultrasound of abdomen ordered for this ED visit

Appendicitis_Dx	num(8)	1=Yes<br>0=No	Appendicitis diagnosis associated with this ED visit
Perforation_Dx	num(8)	1=Yes<br>0=No	Perforation diagnosis associated with this ED visit

List of variables for Hypothesis 2 analysis.

Variable Name	Variable Type and Length (bytes)	Values	Variable description
Study_ID	char(7)	HP00001, HP00002, etc<br>KP00001, KP00002, etc	Study id
Site	num(8)	101=Regions,<br>102=Lakeview,<br>103=Westfield,<br>104=Hudson,<br>105=Methodist,<br>106=Amery<br>201=Sacramento,<br>202=Roseville,<br>203=South San Francisco,<br>204=San Rafael,<br>205=Santa Clara,<br>206=South Sacramento,<br>207=San Leandro,<br>208=Fremont,<br>209=Oakland,<br>210=Richmond,<br>211=San Francisco	Site of Emergency Department
Intervention_arm			

ED_Arrival_DT	num(8)	sas date	ED Arrival Date
ED_Arrival_TM	num(8)	sas time	ED Arrival Time
ED_Arrival_Means	num(8)	1=Car,<br>2=Ambulance,<br>3=Police,<br>4=Helicoptor,<br>5=Other,<br>6=Unknown,<br>7=Newborn,<br>8=Public Transportation,<br>9=Taxi,<br>10=Walk,<br>11=Dropped Off,<br>12=Light Rail,<br>54=Hospital Transport	Means of Arrival to the ED (follow Epic values in ZC_ARRIV_MEANS table)
Age	num(8)	5-20	Age at ED Arrival (in years)
Pat_Enc_CSN_ID	num(8)		Patient Encounter ID that uniquely identifies an ED visit
Provider_ID	char(18)		Provider ID
Provider_Type	num(8)	1=Physician,<br>2=Resident,<br>3=Physician Assistant,<br>4=Nurse Practitioner	Provider Type
Heart_Rate	num(8)	beats per minute	First measurement of heart rate at ED visit
SBP	num(8)	mm HG	First measurement of systolic blood pressure at ED visit
Respiratory_Rate	num(8)	breaths per minute	First measurement of respiratory rate available at ED visit
Temperature	num(8)	degrees celsius	First measurement of temperature available at ED visit

Height	num(8)	inches	First measurement of height available at ED visit. If not available look back for most recent height in past year.
Weight	num(8)	pounds	First measurement of weight available at ED visit
Right_Side_Pain_triage	num(8)	1=Any right sided abdominal pain reported from triage,<br>0=No right sided abdominal pain reported from triage	Designates if any right sided abdominal pain reported by RN or MD in response to triage alert.
Right_Side_Pain_CDS	num(8)	1=Any right sided abdominal pain reported from triage,<br>0=No right sided abdominal pain reported from triage	Designates if any right sided abdominal pain reported by RN or MD in response to triage alert. Confirmed by MD.
Pain_diffuse_triage	num(8)	1=Yes, there is diffuse or generalized abdominal pain,<br>0=No, the pain is not diffuse or generalized	Designates if the pain is diffuse or generalized
Pain_diffuse_CDS	num(8)	1=Yes, there is diffuse or generalized abdominal pain,<br>0=No, the pain is not diffuse or generalized	Designates if the pain is diffuse or generalized. Confirmed by MD.
Pain_5days_or_less_triage	num(8)	1=Pain duration 5 days or less, reported from triage<br>0=Pain duration more than 5 days, reported from triage	Designates if pain duration is 5 days or less at time of ED visit
Pain_Walking_CDS	num(8)	1=yes,<br>0=no	Abdominal pain with walking, jumping, or coughing by history

Pain_duration_CDS	num(8)	1= <12,<br>2=12-23 (<1day),<br>3=24-47 (<2days),<br>4=48-71 (<3days),<br>5=72-120 (<5days),<br>6=120+ (5 days or more)	Pain duration in hours at time of exam
Migration_RLQ_CDS	num(8)	1=yes,<br>0=no	Migration of pain to right lower quadrant by history
Rt_Sided_Tenderness_CDS	num(8)	1=yes,<br>0=no	Does the patient have right sided abdominal tenderness on exam
Max_tenderness_RLQ_CDS	num(8)	1=yes,<br>0=no	Is the abdominal tenderness maximal in the right lower quadrant on exam
Guarding_CDS	num(8)	1=yes,<br>0=no	Is there abdominal guarding on exam
WBC			
Imaging (usage)			

ED_Disposition	num(8)	1=Admit Inpt, 2=Home,<br>3=Transfer to Other Hospital,<br>4=Expired, 5=AMA,<br>6=Clinic,<br>7=Detox, 8=Jail,<br>9=LWBS/LWBF,<br>10=Observation,<br>11=Shelter, 12=NH/TCU,<br>13=Error,<br>14=OR/SDS/L&D (non-admit),<br>15=LWBR, 16=Current Admit,<br>17=Home,<br>18=Hospital Bed,<br>19=Observation,<br>20=Detox, 21=AMA, 22=Clinic,<br>23=Transfer to Other Hospital,<br>24=Jail,<br>25=LWBS/LWBF, 26=Expired,<br>27=Shelter, 28=NH/TCU,<br>29=Error, 30=Surgery/Dialysis,<br>31=Not Registered (LWBR),<br>32=Current Admit,<br>33=Cancel Expected Patient,<br>34=LWBS Before Triage,<br>35=Left Without Being Finished,<br>36=L&D, 40=LWBS After Triage	Disposition of the patient when discharged from the ED
ED_Discharge_DT	num(8)	sas date	ED Discharge Date
ED_Discharge_TM	num(8)	sas time	ED Discharge Time
Definite Discharge diagnosis		Non-perforated appendicitis<br>Perforated appendicitis<br>Other surgical problem	

List of variables for Hypothesis 4.

Variable Name	Variable Type and Length (bytes)	Values	Variable description
Study_ID	char(7)	HP00001, HP00002, etc<br>KP00001, KP00002, etc	Study id
Pat_Enc_CSN_ID	num(8)		Patient Encounter ID that uniquely identifies an ED visit
Adate	num(8)	sas date/time	Date of diagnosis. This table will include all encounters 30 days after index visit
Ddate	num(8)	sas date/time	
ENC_TYPE	num(8)	1=Hospital/ED<br>2=ICU<br>3=Clinic/Other	Designates if the diagnosis was made while in the hospital/ED, ICU. All diagnoses from index visit will have Dx_Hosp_ED='1'. Other diagnoses within 30 days of index visit could have Dx_Hosp_ED= '1' or '2'.
PROC_CODE	char(8)	00100-99607; 0001F-7025F	CPT-4 code
ENC_Source	num(8)	1=Clarity,<br>2=Claims	Source of utilization data.
