# Supplementary material for: Effect of Clinical Decision Support on Diagnostic Imaging for Pediatric Appendicitis: A Cluster Randomized Trial
Source: JAMA Netw Open. 2021 Feb 9;4(2):e2036344. doi: 10.1001/jamanetworkopen.2020.36344 (PMC7873779; doi:10.1001/jamanetworkopen.2020.36344)
Supplement: Supplement 2. — eTable 1. Exclusions Applied in Real Time and Post Hoc and Their Data Sources eTable 2. Recommendations by Pediatric Appendicitis Risk Calculator (pARC) and by Site eTable 3. Secondary Outcomes by Study Group and Phase eTable 4. Outpatient Costs of Care, Excluding Index Emergency Department Visit, by Study Group and Time From Index Visit During Intervention Phase eFigure. Calendar Month and Year for Start and End of Pilot, Preintervention, Washout, and Intervention Phases by Site [file jamanetwopen-e2036344-s002.pdf]

Supplemental Online Content

Kharbanda AB, Vazquez-Benitez G, Ballard DW, et al; Clinical Research on Emergency Services and Treatment Network (CREST); Critical Care Research Center, HealthPartners Institute. Effect of clinical decision support on diagnostic imaging for pediatric appendicitis: a cluster randomized trial. *JAMA Netw Open*. 2021;4(2):e2036344. doi:10.1001/jamanetworkopen.2020.36344

eTable 1. Exclusions Applied in Real Time and Post Hoc and Their Data Sources

eTable 2. Recommendations by Pediatric Appendicitis Risk Calculator (pARC) and by Site

eTable 3. Secondary Outcomes by Study Group and Phase

eTable 4. Outpatient Costs of Care, Excluding Index Emergency Department Visit, by Study Group and Time From Index Visit During Intervention Phase

eFigure. Calendar Month and Year for Start and End of Pilot, Preintervention, Washout, and Intervention Phases by Site

This supplemental material has been provided by the authors to give readers additional information about their work.

© 2021 Kharbanda AB et al. *JAMA Network Open.*

eTable 1. Exclusions Applied in Real Time and Post Hoc and Their Data Sources

**Exclusions applied in real-time, at the point of care**	**Data source(s)**
Pregnancy	EHR flowsheet, EHR diagnoses, problem lists, data entered in AppyCDS
Trauma	EHR flowsheet, EHR diagnoses, data entered in AppyCDS
Prior appendectomy or other abdominal surgery	EHR diagnoses, EHR problem lists
Abdominal pain >5 days duration	Data entered in AppyCDS
Abdominal pain not right-sided or diffuse	Data entered in AppyCDS
Comorbidities[a]	Diagnosis codes, medications ordered, Data entered in AppyCDS (KPNC only)
**Exclusions applied post-hoc, during analyses**	**Data source(s)**
ED visit for abdominal pain in prior 7 days	EHR, claims
Comorbidities	EHR, claims
Index ED visit during washout period	Data entered in AppyCDS

EHR = electronic health record; KPNC = Kaiser Permanente, Northern California; ED = emergency department

[a]Includes: bone marrow transplant, cancer, cystic fibrosis, Down syndrome, diverticulitis, end stage renal disease / dialysis, Henoch Schonlein Purpura, Hirschsprung's disease, HIV, inflammatory bowel disease, intestinal atresia or stenosis, juvenile rheumatoid arthritis, mental retardation, pancreatitis, sickle cell disease, solid organ transplant, systemic lupus erythematous, volvulus

© 2021 Kharbanda AB et al. *JAMA Network Open.*

eTable 2. Recommendations by Pediatric Appendicitis Risk Calculator (pARC) and by Site

pARC	KPNC – Intervention recommendations	HP – Intervention recommendations
<5%[a]	Outpatient follow-up, no imaging	Outpatient follow-up, no imaging
6-15%	Outpatient follow-up, no imaging; Consider observation in ED	Outpatient follow-up, no imaging; Consider observation in the ED
16-25%	If pain <24 hours, observation in ED, US if pain persists; If pain ≥24 hours, order US and/or surgery consult; admit if US equivocal	If pain <24 hours, observation in ED, US and repeat CBC if pain persists; If pain ≥24 hours, order US
26-50%	US as first line imaging, and/or surgery consult;	US as first line imaging; admit if US equivocal
51-75%	US as first line imaging, and/or surgery consult; CT if US equivocal	US as first line imaging; CT if US equivocal
76-90%	Obtain US and Consult surgery	Consult surgery, consider imaging
>90%	Consult surgery	Consult surgery, imaging not routinely required

pARC = pediatric appendicitis risk calculator; KPNC = Kaiser Permanente Northern California; HP = HealthPartners; ED = emergency department; US = ultrasound; CT = computed tomography; CBC = complete blood count

[a]Or physician gestalt ≤10% at KPNC

© 2021 Kharbanda AB et al. *JAMA Network Open.*

eTable 3. Secondary Outcomes by Study Group and Phase

	Intervention Arm		Usual Care Arm	
	Pre-Intervention phase n = 1,011	Intervention phase n = 2,150	Pre-Intervention phase n = 788	Intervention phase n = 1,991
Mean ED length of stay, 95% CI, hours	3.4 (3.3-3.7)	3.5 (3.4- 3.7)	3.7 (3.5-3.9)	3.7 (3.5-3.8)
Disposition, n (%)				
Discharge home	845 (83.6)	1782 (82.9)	657 (83.4)	1660 (83.4)
Inpatient admission	110 (10.9)	261 (12.1)	94 (11.9)	257 (12.9)
ED or hospital transfer	56 (5.5)	107 (5.0)	37 (4.7)	74 (3.7)
ED visit within 7 days of index encounter, n (%)	72 (7.1)	150 (7.0)	58 (7.4)	166 (8.3)
ICU admission within 7 days of index encounter, n (%)	0 (0.0)	6 (0.3)	1 (0.1)	5 (0.3)
Severe medical diagnoses[a], n (%)	12 (1.2)	17 (0.8)	4 (0.5)	19 (1.0)
Other surgical diagnoses[b], n (%)	4 (0.4)	6 (0.3)	1 (0.1)	10 (0.5)

ED = emergency department; ICU = intensive care unit

[a]Severe medical diagnoses include small bowel obstruction, tubo-ovarian abscess, pelvic inflammatory disease, pyelonephritis

[b]Other surgical diagnoses include ovarian torsion, testicular torsion, abdominal tumor, intussusception, volvulus, ectopic pregnancy

© 2021 Kharbanda AB et al. *JAMA Network Open.*

eTable 4. Outpatient Costs of Care, Excluding Index Emergency Department Visit, by Study Group and Time From Index Visit During Intervention Phase

Cost category	Intervention Arm n=1,372	Usual Care Arm n=1,290	Difference (95% CI)	p-value
All outpatient costs (excluding index ED visit)	$360	$410	-$50 (-$110 to $9)	0.098
Within 3 days of index visit	$284	$302	-$18 (-$68 to $33)	0.486
Clinic	$69	$77	-$8 (-$26 to $10)	0.390
ED	$59	$52	$7 (-$19 to $33)	0.599
Same-day surgery	$103	$102	$1 (-$46 to $48)	0.954
Other	$48	$71	-$23 (-$53 to $7)	0.130
Days 4-14 from index visit	$78	$108	-$31 (-$47 to -$15)	<0.001
Clinic	$36	$39	-$2 (-$11 to $7)	0.636
ED	$16	$28	-$13 (-$24 to -$1)	0.030
Same-day surgery	$3	$16	-$12 (-$21 to -$3)	0.007
Other	$22	$32	-$10 (-$16 to -$4)	0.002

ED = emergency department; CI = confidence interval; CT = computed tomography. All costs are measured in 2018 U.S. dollars over 14 days following the index ED visit for patients in each study arm during the intervention phase. Other includes costs for urgent care, hospital ambulatory, and rehab services. Subgroup totals will not necessarily sum to grand totals due to independently modeling costs within each group while accounting for cluster randomization across two sites.

© 2021 Kharbanda AB et al. *JAMA Network Open.*

eFigure. Calendar Month and Year for Start and End of Pilot, Preintervention, Washout, and Intervention Phases by Site

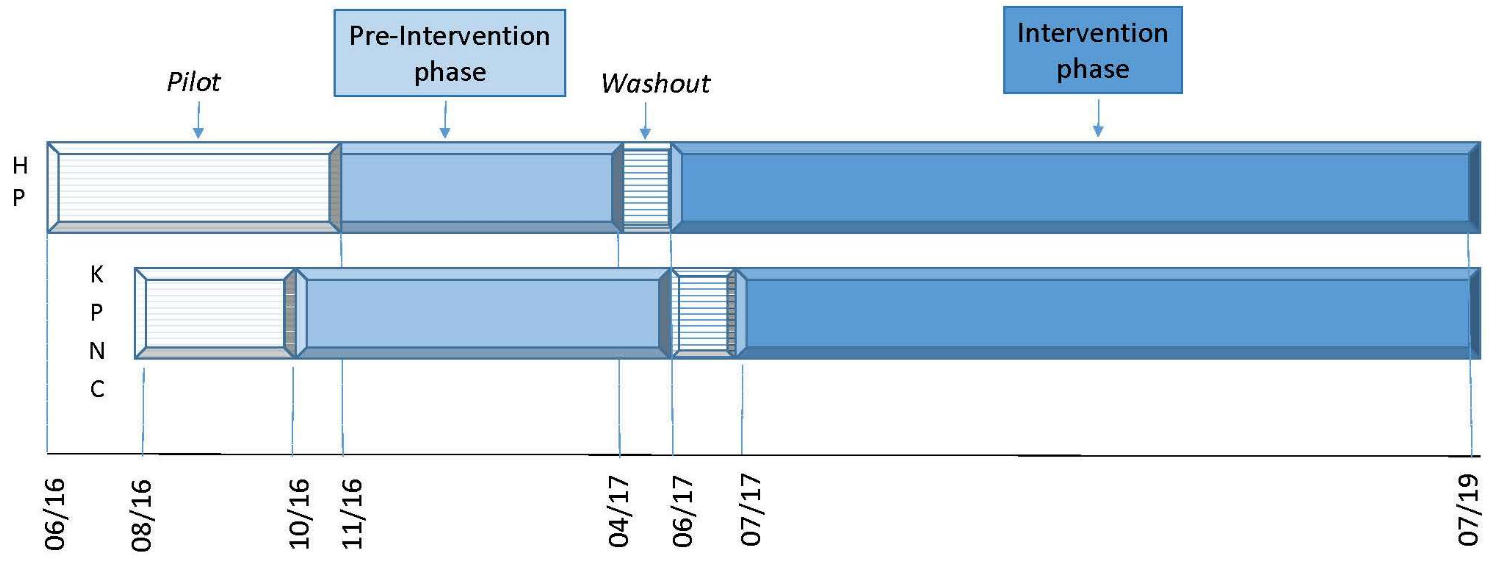

© 2021 Kharbanda AB et al. *JAMA Network Open.*
