# Supplementary material for: Effect of Clinical Decision Support on Diagnostic Imaging for Pediatric Appendicitis: A Cluster Randomized Trial
Source: JAMA Netw Open. 2021 Feb 9;4(2):e2036344. doi: 10.1001/jamanetworkopen.2020.36344 (PMC7873779; doi:10.1001/jamanetworkopen.2020.36344)
Supplement: Supplement 3. — Data Sharing Statement [file jamanetwopen-e2036344-s003.pdf]

Data Sharing Statement

Kharbanda. Effect of Clinical Decision Support on Diagnostic Imaging for Pediatric Appendicitis. *JAMA Netw Open*. Published February 09, 2021. doi:10.1001/jamanetworkopen.2020.36344

Data
Data available: Yes
Data types: Data dictionary
How to access data: Heidi.L.Ekstrom@healthpartners.com
When available: With publication

Supporting Documents
Document types: Statistical/analytic code
How to access documents: Heidi.L.Ekstrom@healthpartners.com
When available: With publication

Additional Information
Who can access the data: Researchers whose proposed use of the data has been approved.
Types of analyses: Sub-group analyses, Validation.
Mechanisms of data availability: With signed Data access agreement, after review of study protocol.
